# Precision Livestock Farming Research: A Global Scientometric Review

**DOI:** 10.3390/ani13132096

**Published:** 2023-06-24

**Authors:** Bing Jiang, Wenjie Tang, Lihang Cui, Xiaoshang Deng

**Affiliations:** 1 College of Economics and Management, Northeast Agricultural University, Harbin 150030, China; s220801029@neau.edu.cn (W.T.); b210801002@neau.edu.cn (L.C.); a08200873@neau.edu.cn (X.D.); 2 Development Research Center of Modern Agriculture, Northeast Agricultural University, Harbin 150030, China

**Keywords:** precision livestock farming, animal welfare, bibliometrics, CiteSpace

## Abstract

**Simple Summary:**

In recent years, there has been a significant increase in research on precision livestock farming. The aim of this paper is to provide a comprehensive review of the current state of research on precision livestock farming. Using the visualization tool CiteSpace, this study creates knowledge maps to display data on research countries, institutions, author collaborations, and keyword networks. Through these analyses, this study objectively reveals the dynamics, development process, and evolutionary trends of precision livestock farming research while identifying the frontiers and hotspots in the field.

**Abstract:**

Precision livestock farming (PLF) utilises information technology to continuously monitor and manage livestock in real-time, which can improve individual animal health, welfare, productivity and the environmental impact of animal husbandry, contributing to the economic, social and environmental sustainability of livestock farming. PLF has emerged as a pivotal area of multidisciplinary interest. In order to clarify the knowledge evolution and hotspot replacement of PLF research, based on the relevant data from the Web of Science database from 1973 to 2023, this study analyzed the main characteristics, research cores and hot topics of PLF research via CiteSpace. The results point to a significant increase in studies on PLF, with countries having advanced livestock farming systems in Europe and America publishing frequently and collaborating closely across borders. Universities in various countries have been leading the research, with Daniel Berckmans serving as the academic leader. Research primarily focuses on animal science, veterinary science, computer science, agricultural engineering, and environmental science. Current research hotspots center around precision dairy and cattle technology, intelligent systems, and animal behavior, with deep learning, accelerometer, automatic milking systems, lameness, estrus detection, and electronic identification being the main research directions, and deep learning and machine learning represent the forefront of current research. Research hot topics mainly include social science in PLF, the environmental impact of PLF, information technology in PLF, and animal welfare in PLF. Future research in PLF should prioritize inter-institutional and inter-scholar communication and cooperation, integration of multidisciplinary and multimethod research approaches, and utilization of deep learning and machine learning. Furthermore, social science issues should be given due attention in PLF, and the integration of intelligent technologies in animal management should be strengthened, with a focus on animal welfare and the environmental impact of animal husbandry, to promote its sustainable development.

## 1. Introduction

The livestock industry serves as the foundation of the agricultural economy and constitutes the primary source of animal-derived product consumption. The growing demand for these products has led to a significant expansion in the scale of livestock breeding. However, traditional management practices that rely on farmers’ observations, judgment, and experience alone may not fulfill the requirements of modern large-scale livestock farming. Therefore, precision farming, with the support of information technology, has become an unavoidable trend in advancing modern livestock farming.

Since the 1970s, PLF technology has evolved considerably, beginning with individual electronic milk meters for cows and expanding to include behavior-based estrus detection, rumination activity monitoring and other related studies. Scholars have conducted extensive research on intelligent perception and analysis of individual animal information and behavior [1]. In 2003, the European Conference on Precision Livestock Farming (EC-PLF) was launched and held every two years to highlight the latest high-tech research advances in livestock farming. In 2012, The European Union launched the European Precision Livestock Farming Project (EU-PLF), with a mission to translate PLF technology into industrial practice. Participants in the project included influential universities in the field of PLF, such as Wageningen University and Research, Katholieke Universiteit Leuven and the University of Milan, as well as companies dedicated to developing PLF technology, including Facom, Soundtalks NV and GEA Farm Technologies. The project quantified key PLF indicators and guided the use of PLF systems such as video monitoring, sound monitoring, and environmental monitoring on farms, playing a vital role in promoting the research and application of PLF technology [2].

Due to the urgent requirement for integrating information technology, data science, artificial intelligence, and innovative animal husbandry development, China’s animal husbandry industry is rapidly advancing into a new era of integrated fusion innovation [3]. In 2012, the State Council issued the Opinions on Accelerating Agricultural Science and Technology Innovation to Continuously Enhance the Ability to Guarantee the Supply of Agricultural Products, in which it was proposed that a major breakthrough should be made in precision agriculture technology; in 2020, the Opinions on Promoting the High-Quality Development of the Livestock Farming Industry issued by the General Office of the State Council clearly proposed that the application of technologies such as big data, artificial intelligence, cloud computing, Internet of Things and mobile Internet in the livestock industry should be strengthened, and the intelligence level of environmental control of enclosures, precise feeding and animal disease monitoring should be improved. Driven by the policy, the development and application of precision technology in the livestock industry has received widespread attention. In recent years, the research on PLF in China has made great progress in animal respiration frequency detection, individual identification and behavioral analysis [4,5,6]. A lot of improvements and innovations have been made in the automated detection of animal body size and weight [7,8], and equipment and algorithms for environmental monitoring and prediction in livestock housing have been refined [9,10].

PLF is a multidisciplinary concept integrating information technology, data science, and innovative animal husbandry. Five primary research perspectives drive existing PLF research: animal science, veterinary medicine, computer science, agricultural engineering, and environmental science. The animal science perspective aims to optimize animal feeding and management by leveraging sensors and intelligent monitoring systems to real-time monitor and analyze animal behavior, physiological states, and environmental factors [11,12]. The veterinary medicine perspective focuses on intelligent prevention and diagnosis of animal diseases [13,14]. The computer science perspective focuses on the application of sensor networks, artificial intelligence, computer vision and other technologies in animal data monitoring, mining and collection [15,16]. The agricultural engineering perspective focuses on the mechanization of livestock farming and its automation technology to help farmers better manage their farms [17,18]. The environmental science perspective evaluates the environmental impact of using PLF technology as a mitigation strategy for livestock production [19,20]. The existing research on PLF has primarily focused on its technical aspects from a natural science perspective. However, there has been limited discussion on the evolution of basic knowledge and the emergence of new research focal points. In order to address this gap, this study examines PLF-related literature in the Web of Science database from 1973 to 2023. By utilizing the visualization tool CiteSpace, this study creates knowledge graphs to display data such as the research countries, institutions, author collaborations, and keyword networks. Through this analysis, this study objectively reveals the dynamics, developmental processes, and evolution trends of PLF research, while identifying frontiers and hotspots within the field. Ultimately, the aim of this study is to provide a comprehensive overview of PLF research status and scientific references for future research.

## 2. Concept of PLF

PLF integrates precision agriculture concepts to help farmers manage large-scale livestock farming through the use of sensors and actuators, representing the application of PA in livestock systems [21]. Daniel Berckmans first coined the term PLF and argued that continuous, direct, real-time monitoring or observation of animal status through PLF would enable farmers to rapidly identify and control problems related to animal health and welfare [22]. As scholars have explored PLF, a more unified perception of its concept has gradually emerged. PLF is a method for fine-grained management of modern livestock farming using process engineering principles and techniques [2,23,24], animal science [25] and information technology [2,25,26], and is a set of technologies used to monitor and control animal health, welfare, production, reproduction and environmental impacts in real-time [27,28,29], aiming to provide stakeholders with information as a basis for management decisions [24,30] to improve the management of large-scale livestock and poultry [21,31] to achieve economically, socially and environmentally sustainable farming [19,21,23]. Combining the above concepts, this study concludes that PLF is a series of fine management methods supported by information technology, based on real-time data collection and analysis, with the intelligent sensing and analysis of individual animal information and behavior as the core, aiming to improve animal productivity and animal welfare.

The widespread use of digital technologies has given birth to smart livestock farming (SLF) and digital livestock farming (DLF), whose concepts have some overlap and crossover with PLF. In order to clarify the connotation of PLF, it is necessary to further explore the concepts of SLF and DLF. In much of the literature, SLF is often attributed to PLF [31,32], but recent research proposes that SLF should be considered more of a successor to PLF [33]. PLF focuses on the digital processing of specific information to support stakeholder decision-making, while SLF is a knowledge-based concept that leverages information and communication technology (ICT) to manage cyber-physical livestock farms [33,34]. DLF incorporates the concepts of precision and smart farming that use modern technological tools, advanced equipment and comprehensive data management; it provides important insights, modeling approaches and actionable analytics and automation techniques to provide efficient, accurate and intelligent solutions for livestock farming [33]. The focus of DLF development is no longer on mere accuracy, but on integrating precise data into digital systems, achieving a transcendence of PLF [33]. Both SLF and DLF can be seen as PLF as a natural development based on PLF, reflecting the increasing integration of digital technologies in animal husbandry. The promotion and application of these new models will further improve the productivity and quality of animal husbandry, safeguard animal welfare, and promote the sustainable development of animal husbandry.

## 3. Materials and Methods

### 3.1. Data Collection

Literature data collection is essential for review papers because its quantity and quality directly determine the effectiveness of article visualization [35]. In order to acquire high-quality literature pertaining to PLF research, we initially narrowed our search to prestigious academic journals, such as SCI and SSCI. Furthermore, to ensure that the collected literature was academically novel, we exclusively considered “Article” and “Review” document types. Finally, while meeting the above criteria, we aimed to collect as much literature data as possible, and thus chose the Web of Science database as our source of literature data. The Web of Science database is a major citation index database that has 10 sub-data sets, covering different time spans, over 21,000 high-quality academic journals in various fields, and 1.7 billion citations [36]. To ensure the comprehensiveness of the literature search, multiple search terms were set. The search terms used were: TS = (“accelerometer” or “sensor” or “GPS” or “automate” or “machine learning” or “big data” or “robot” or “computer vision” or “deep learning”) AND TS = (“cattle” or “cow” or “sheep” or “poultry” or “broiler” or “hen” or “pig”); TS = (“ruminal boluses”) AND TS = (“cattle” or “cow” or “sheep”); TS = (“PLF”), the retrieval period was “all years (1950–2023)”, the citation index was limited to “SCI-EXPANDED” and “SSCI”, and the document type was “Article” and “Review”. The preliminary search yielded 14,762 papers. These obtained papers were then re-evaluated by reading the titles and abstracts, and after removing duplicates and irrelevant content, 3658 papers were finally obtained that were highly relevant to the PLF topic.

### 3.2. Research Methodology

Bibliometrics employs mathematical and statistical methods to analyze literature quantitatively. It is widely used in various fields, including literature statistics, assessing the impact of journals or research institutions, tracking academic hotspots, and predicting research trends [37]. As such, bibliometrics offers a more objective view of research progress in specific fields of knowledge.

CiteSpace, a bibliometric analysis tool developed using Java language by Professor Chaomei Chen from Drexel University, applies co-citation analysis theory, pathfinding network algorithm, and minimum spanning tree algorithm to quantitatively analyze specific literature data [38]. It generates a series of visual graphs to detect the frontier of discipline development and provide an analysis of the potential dynamic mechanism of discipline evolution [39]. In CiteSpace networks, nodes represent different elements such as countries, institutions, and authors, where the size of a node indicates its frequency of occurrence, and connecting lines between nodes demonstrate cooperative links [40]. To quantify the significance of each node in the network, a centrality indicator known as betweenness centrality is used. A node with centrality > 0.1 is highlighted with a purple ring to emphasize the key points in the network [41]. The formula for calculating the betweenness centrality of each node is provided as follows:

(1) $$Centrality\left({node}_{i}\right)=\sum_{i\neq j\neq k}\frac{\rho_{jk}(i)}{\rho_{jk}}$$

In Equation (1), $\rho_{jk}$ refers to the number of shortest paths between node *j* and node *k*, with $\rho_{jk}(i)$ representing the number of those paths passing through node *i* [42].

Price Law is commonly used to measure the distribution of core authors across disciplines and data analysis, which is generally applicable to most research fields with a long statistical time and a large author collection, and the method often appears in bibliometric-related articles [37,38,43]. The formula is as follows:

(2) $$TP_{n}\approx 0.749\sqrt{N_{max}}$$

In Equation (2), $TP_{n}$ represents the threshold value of the number of papers published by core authors, and $N_{max}$ represents the number of papers published by the most productive authors. When the number of papers published by an author is $TP_{n}$ and above, he or she is identified as a core author in this research field. According to Price Law, a core group of authors is formed when the number of papers published by core authors reaches half of the total number of research papers in a field [43].

Bradford’s law is an empirical law describing the law of literary dispersion, which is applied to measure the degree of connection between disciplines and describe the distribution of the number of relevant papers in published journals [43]. Bradford’s law posits that when journals are arranged in decreasing order based on the number of papers published on a subject, they can be categorized into core, relevant, and irrelevant regions, with approximately equal numbers of papers published in each region. Moreover, the number of journals in the three regions has a 1: a: a^2^ relationship (a ≈ 5) [37]. Journals in core areas are considered to represent the latest level of research in a specific field [43].

In this study, the scientific knowledge graphs and empirical laws in bibliometrics were selected, with the help of the visual tool CiteSpace (6.2. R2), the research on PLF was visually analyzed. The parameters were set as follows: (1) time slicing from 1973 to 2023 at 1 year per slice; (2) the selection uses a modified g-index in each slice: k = 25, which means that data were extracted on the top 25 results for each time slice; (3) the node type was set as country/institution/author/keyword. The remaining parameters were the default settings. By drawing relevant knowledge graphs and charts, the research status, hotspots and dynamic frontiers of PLF were combed and summarized.

## 4. Results and Discussion

### 4.1. Analysis of Main Features

#### 4.1.1. Annual Scientific Production

**Figure 1:**
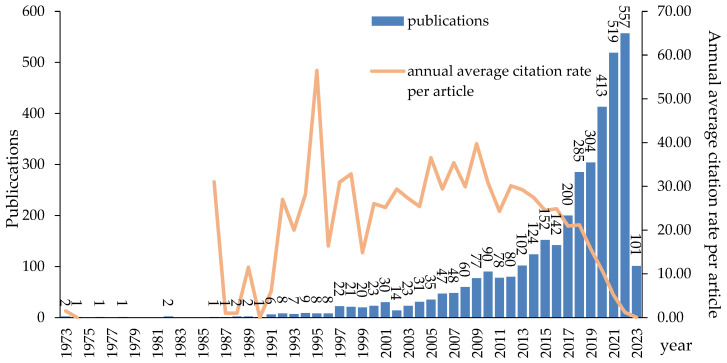
Number of publications and annual average citation rate per article as determined using Web of Science data.

The annual number of PLF publications is a critical indicator of the discipline’s developmental level and research achievements, and evaluating the academic influence of research papers can be aided by the calculation of the annual average citation rate per article [44]. Figure 1 displays number of publications and annual average citation rate per article associated with PLF research. The citation frequency of recent publications has been relatively low due to the time it takes for important articles to be recognized and become widely cited, resulting in a fluctuating downward trend in the annual average citation rate of PLF research papers. The paper “Segmentation and Tracking of Piglets in Images” published by N.J.B. McFarlane and C.P. Schofield in 1995 has been widely cited. The two scholars developed an algorithm for segmenting and tracking piglets, providing valuable experience for subsequent improvement of tracking feature extraction and related algorithms [45]. The upward trend in the number of PLF publications between 1973 and 2023 suggests a growing interest in this research field. This trend may be divided into three phases: the germination stage (1973–1996), the exploration stage (1997–2016), and the rapid development stage (2017–2023). In the germination stage, limited by the level of animal husbandry development and information technology understanding, research content was relatively scant. Automatic milking and estrus detection were common topics [46,47]. The exploration stage focused on animal health and welfare due to concerns over disease transmission from animals to humans [22]. Scholars also focused on animal behavior monitoring and analysis, in addition to research on machine learning systems for livestock classification, pattern recognition, optimization, and prospective prediction [48]. As big data and cloud computing technologies matured and production demands grew, managing animals manually became more challenging [29], leading to a rapid increase in PLF literature. From 2017 to 2023, there were 2379 articles, accounting for 65.04% of the total literature sample. Researchers showed increased interest in artificial intelligence applications in livestock farming, such as deep learning and machine learning, leading to a rapid stage of PLF development.

#### 4.1.2. Countries

**Figure 2:**
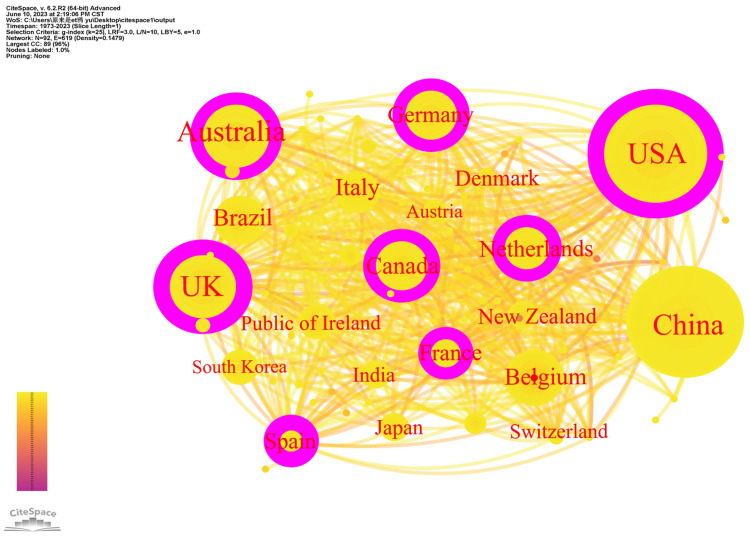
Countries co-occurrence network.

The analysis of the countries involved in research can uncover their collaborative relationship and provide a new perspective for evaluating countries’ academic influence [49]. Literature data were imported into CiteSpace software, with the node type of the functional selection area set to COUNTRY for analyzing all countries engaged in PLF research. A threshold value of 70 was set to highlight the countries with 70 or more papers, resulting in the creation of the co-occurrence network of countries (Figure 2).

The distribution of connections between countries shows a cluster-like pattern with relatively tight links. Figure 2 displays 92 nodes and 619 connections, and the density of the national cooperation network is 0.1479, which is an indication of strong international collaboration in this field. The top five countries in the number of publications are the USA, China, UK, Australia, and Germany, all of which are major livestock production nations. PLF research is primarily concentrated in developed countries. The USA, as the country with the highest degree of modernization in livestock development, leads PLF research, accounting for 19.08% of the total literature [50]. As the world’s largest livestock producer, China’s livestock industry is transitioning towards scale and modernization, demanding information technology and intelligent farming technology. China launched various research and development programs since 2016, such as key technology and equipment for intelligent sensing of livestock and poultry breeding, and relevant enterprises and research institutes have carried out multi-level research and development and practice to promote smart livestock farming and unmanned pastures. Scholars focus on PLF research, with publications accounting for 11.45% of the total literature. UK, Australia and Germany are leaders in well-developed facility animal husbandry. They emphasize promoting the industrialization of livestock farming, intelligent sensing technology, and education and training to cultivate highly qualified farmers and herdsmen [51]. These countries have extensive applications of PLF technology.

Quantifying the importance of nodes in a network can be accomplished by measuring node centrality [49]. Combining the centrality to analyze the influence of the research countries (Table 1), five countries stand out as having key influence in the field of PLF: USA, Spain, UK, Netherlands, and France. The international influence of the USA, Spain, and UK is particularly evident with centrality measures above 0.2. Although China has a high number of publications, it does not have significant centrality advantages compared to other countries. In contrast, Netherlands, France, and Spain, while not as numerous as China, have relatively high centrality levels and concentrate more on the influence of international cooperation. Developed countries have an early start in PLF research, more experience in related technology development and application, and closer communication and collaboration among countries while developing countries need to further strengthen cross-country cooperation in PLF.

#### 4.1.3. Institutions

**Figure 3:**
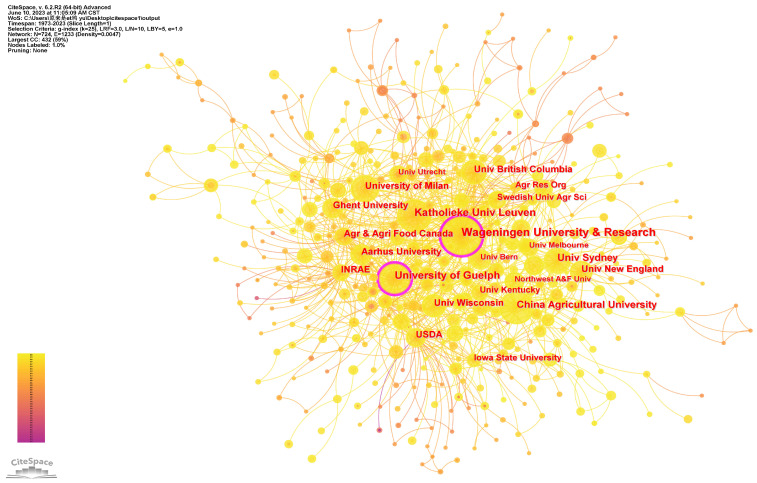
Institution co-occurrence network.

Analyzing the structural characteristics of research institutions is essential to identify influential institutions in the field and understanding inter-institutional cooperation [35,52]. To analyze the institutions involved in PLF research, we designated the node type as INSTITUTION in the CiteSpace function selection area. We then set the threshold value to 30 to highlight institutions with 30 or more papers, resulting in an institution co-occurrence network (Figure 3). The top 20 institutions for the period 2019–2023 can be found in Supplementary Material Table S1.

The distribution of research institutions in PLF is shown in Figure 3. Wageningen University and Research, Katholieke Universiteit Leuven, University of Guelph, China Agricultural University and University of Sydney have a high number of publications and are representative institutions for PLF research. Wageningen University and Research is recognized as a world leader in agricultural and environmental sciences, with top-ranked disciplines in agricultural science, plant and animal science, and environmental science. The Katholieke Universiteit Leuven is highly ranked for mechanical engineering and has made significant contributions to the development and utilization of livestock technologies. The University of Guelph ranks in the top 10 to 50 globally for agronomy, veterinary medicine and environmental science and spends over half its budget on academic research. The University of Sydney is one of Australia’s top universities and has a prominent position in the field of agricultural sciences, artificial intelligence, and veterinary medicine. As the leading agricultural institution in China, China Agricultural University has a primary objective of promoting national innovation in agricultural science and technology and advancing modern agriculture. Its agricultural engineering discipline is of national key first-class and world-class standard, with several key laboratories focusing on research into the integration of intelligent agriculture systems and acquisition of agricultural information technology, among others, which have made invaluable contributions to PLF research. National research institutions such as INRAE and government departments such as Agriculture and Agri-Food Canada are also important in promoting the modernization of livestock farming and play a significant role in PLF research. From the attributes of the research institutions (Table 2), the top 20 research institutions are mainly concentrated in universities in various countries, indicating that academic institutions attach great importance to PLF research and have made great contributions, being the main force in this field. From the geographical viewpoint of the research institutions, the main research institutions are unevenly distributed globally, with a concentration in Europe and North America.

In terms of research institutions’ collaboration, the density of the PLF cooperative network is 0.0047, indicating that the cooperative network is relatively sparse. While the centrality of Wageningen University and Research and the University of Guelph is significantly higher than that of other institutions and has an important connecting role in the cooperative network, the level of connection and collaboration between other universities and research institutes remains low. The relative independence of research led by each institution has led to a clear pattern of network segmentation. The deepening of connections between each representative institution is necessary to address this issue, as an academic community for PLF is yet to be formed.

#### 4.1.4. Authors

**Figure 4:**
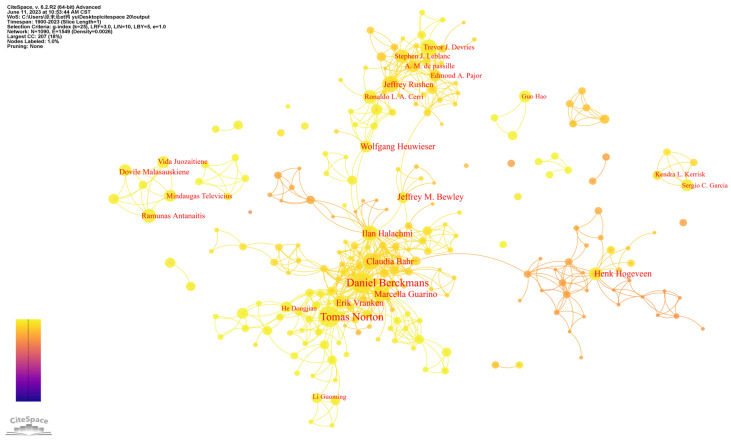
Author co-occurrence network.

Analyzing the structural characteristics of published authors helps to identify core authors in the research field, which further reflects the collaborative relationships among them [40]. Employing Price’s Law, we calculated that the number of papers published by core authors in PLF research exceeded six, resulting in 52 authors classified as core authors. The number of publications by these core authors totaled 713, which accounts for 19.49% of the total literature. A stable cooperative network should ideally reach 50%, indicating that a stable core author group has not yet been formed in this research field [53]. By setting the node type to AUTHOR in CiteSpace’s function selection area, we analyzed all authors involved in PLF research. We set the threshold to 10 to highlight the most prolific authors and obtained an author co-occurrence network (Figure 4). Table 3 presents the top 10 most prolific authors.

The authors’ cooperative network consists of 1090 nodes and 1549 connections, with a density of 0.0026, indicating that while there are many scholars in PLF research, their cooperative relationship is not strong, resulting in “small concentration and large dispersion” characteristics. The concentration of authors is high, and most scholars have formed a relatively fixed cooperative group, which has initially formed a research group led by the core scholars Daniel Berckmans. However, there is still a need for stronger academic connections between different academic teams and authors, and the related research is scattered.

The authors’ research directions span a wide range of topics. Daniel Berckmans is a renowned PLF developer who focuses on creating real-time algorithms to monitor and improve the lives of individual humans and animals. Tomas Norton collaborates with Berckmans frequently, and his current research primarily involves the development of PLF technologies for animal health and welfare monitoring and management. Ilan Halachmi specializes in animal science, artificial intelligence, lameness, and computer vision. Claudia Bahr concentrates on utilizing automated animal monitoring techniques, including disease detection and lameness detection. Marcella Guarino’s research encompasses animal behavior, disease, production and welfare monitoring, and SLF. Jeffrey Rushen mainly deals with animal science, especially dairy cattle, lameness, animal-assisted therapy, and welfare and conducts research on cow lameness automatic detection and cow gait assessment. Trevor J. Devries’ primary areas of study are animal science, dairy cattle, milking and feeding behavior, including research on automatic milking systems and monitoring systems. Henk Hogeveen emphasizes animal health management and has achieved many research advancements in mastitis detection on dairy farms. Jeffrey M. Bewley’s focus is on precision dairy farming, the modernization of dairy facilities, and cow comfort and health. Sergio C. Garcia’s main interest is cow management and performance improvement through automatic milking systems.

#### 4.1.5. Journals

**Figure 5:**
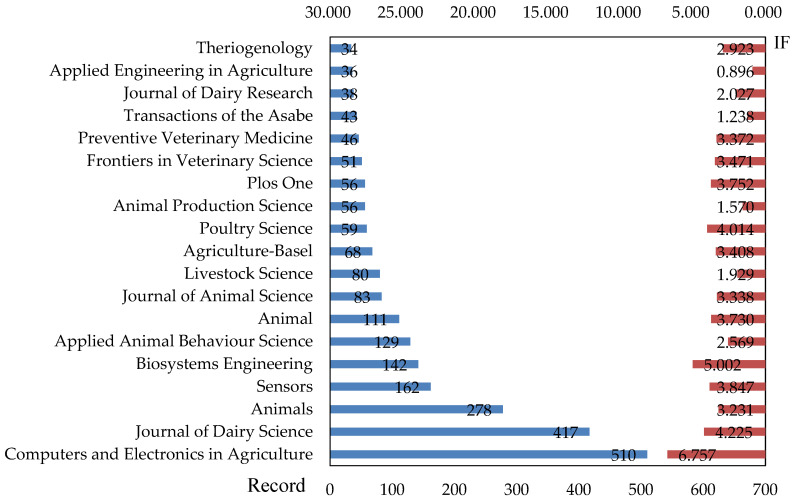
Journals in the core zone.

Examining the structural characteristics of relevant journals can provide direction for literature collection and prior knowledge accumulation in the field, while also reflecting the theoretical and practical value of research within the area [54]. The 3658 sample papers on PLF research were published across 598 journals, with 19 core journals identified in accordance with Bradford’s law, as depicted in Figure 5.

Computers and Electronics in Agriculture, Journal of Dairy Science, Animals, Sensors, and Biosystems Engineering are the top five journals in terms of publication volume. These five journals account for approximately 41.25% of the analyzed publications. According to JCR 2021, their average impact factor is 4.612, which indicates the development of influential perspectives within the field of PLF. Therefore, researchers engaged in relevant studies should keep track of the progress of these journals for high-quality PLF-related research. Based on impact factor, Computers and Electronics in Agriculture were ranked fourth and 23rd in the agriculture, multidisciplinary category and the computer science, interdisciplinary applications category, respectively, with Q1 ratings in both categories. Journal of Dairy Science was ranked sixth and 48th in the agriculture, dairy and animal science category and the food science and technology category, respectively, with Q1 and Q2 ratings in each of these categories. Animals were ranked 13th and 16th in the agriculture, dairy and animal science category and the veterinary sciences category, respectively, with Q1 ratings in both categories. Sensors was ranked 29th, 95th, and 19th in the chemistry, analytical category, engineering, electrical and electronic category, and the instruments and instrumentation category, respectively, with Q2 ratings for all three categories. Finally, Biosystems Engineering was ranked fourth and eighth in the agricultural engineering category and the agriculture, multidisciplinary category, respectively, with Q2 and Q1 ratings in these categories. These 19 journal subject categories relate mainly to animal science, veterinary science, computer science, agricultural engineering, and environmental science, highlighting the multidisciplinary research nature of PLF. Among these categories, animal science had the largest number of journals, and relevant journal publications accounted for approximately 34.20% of the analyzed publication, indicating that PLF is a hot topic of interest in animal science research.

### 4.2. Analysis of Research Cores

#### 4.2.1. Research Hotspots

Keywords constitute the core and essence of a paper as they offer a high-level summary of its content [49]. Analyzing keywords visually can facilitate the identification of hot topics and emerging trends in the target field [35].

**Figure 6:**
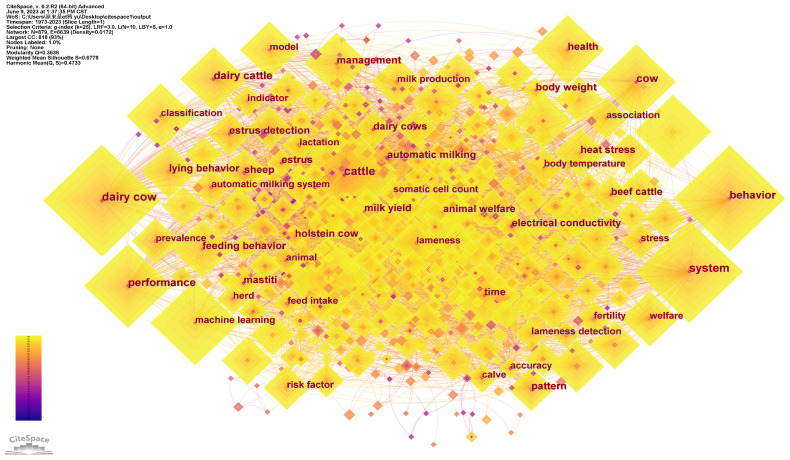
Keyword co-occurrence network.

The higher the frequency of keywords, the stronger their research popularity in a certain field. To obtain the keyword co-occurrence network (Figure 6), we set the node type to KEYWORD and the threshold value to 55. Combined with Table 4, “dairy cow” is the most frequently co-occurring keyword, followed by “cattle”, “behavior”, “system”, and others. Due to their high economic value and close relationship with human nutrition and health [25], “dairy cow” and “cattle” have become the focus of PLF research. The keyword “behavior” ranks third in frequency, and its related research includes animal behavior monitoring [55], behavior recognition [56], and behavior analysis [57]. Systems belong to PLF technology, and relevant research includes intelligent decision support systems [58], machine learning systems [48], electronic nose systems [59], and precision pig breeding systems [57], among others.

The top 20 high-frequency keywords can be categorized into three dimensions: (1) PLF technology, mainly including “system”, “machine learning”, “deep learning”, “model”, and “computer vision”. The technical methods used in PLF rely on systematic data collection and analysis and combine with machine learning, deep learning, and other technologies to achieve dynamic monitoring and management of livestock and poultry. This improves the efficiency of livestock and poultry breeding and the quality of livestock products. (2) The application objects of PLF technology, mainly including “dairy cow”, “cattle”, “cow” and “dairy cattle”. Most applications of PLF technology are based on monitoring devices attached to the animals’ necks, legs, and ears, with large animals providing more space for these devices; moreover, large animals have higher economic value [1]. Therefore, PLF technology is currently mainly applied to large animals. (3) The uses of PLF technology, mainly including “health”, “management”, “classification”, “animal welfare”, and “milk yield”. PLF technology can realize intelligent sensing, warning, and analysis of the livestock production environment. It provides accurate breeding, visual management, and intelligent decision-making for livestock production and effectively improves animal living conditions and welfare.

#### 4.2.2. Research Directions

**Figure 7:**
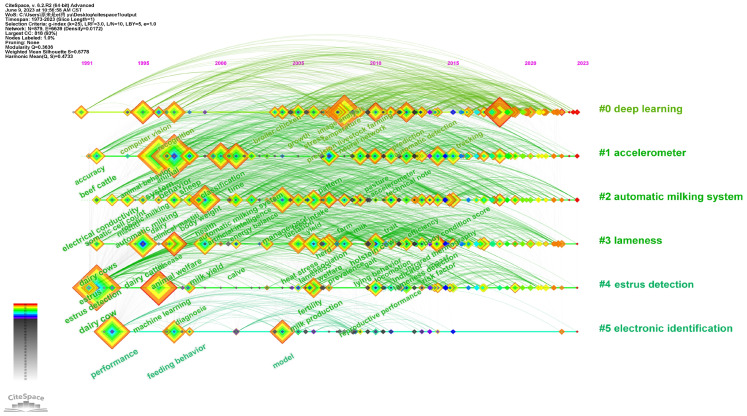
Timeline view of keyword network.

Keyword clustering analysis highlights the relationship between research directions and keywords, and a timeline analysis of keywords is to reveal the relationship between clusters further and the historical span of keyword sets [40,60]. Using CiteSpace software, we obtained the timeline network (Figure 7) and clustering information table (Table 5) for the PLF research’s keyword network. The horizontal axis indicates the year, and the vertical axis indicates the cluster. The higher the rank of the cluster label and the larger its size, the more keywords it includes. We selected the top six clusters with the largest size for analysis.

Cluster #0 is deep learning, which incorporates 168 articles from 1991 to the present, encompassing hot words such as deep learning, computer vision, image processing, precision livestock farming, and object detection. Deep learning is a subset of machine learning that utilizes neural networks with three or more layers. These networks are designed to mimic the human brain’s behavior and learn from vast amounts of data. In recent years, deep learning has fueled the rise of many artificial intelligence applications and services that increase automation [61,62]. Within the field of PLF, deep learning is primarily utilized for animal behavior analysis on farms, tracking poultry and livestock’s health status, behavior habits, and production performance in real-time, and generating personalized management recommendations through data analysis. A convolutional neural network architecture was proposed by Pu et al. for recognizing chicken behavior within poultry farms, and this method achieves an accuracy rate of 99.17%, successfully resolving herd behavior image classification problems [63]. Wu et al. proposed a classification method for identifying lame cattle based on the YOLOv3 deep learning algorithm and relative step feature vector, which can intelligently detect lameness and improve dairy cow welfare [64]. Zhang et al. proposed EFMYOLOv3, a deep learning network based on bilateral filtering enhancement of thermal images, which automatically detects the eyes and breasts of dairy cows, enabling automatic recognition of dairy mastitis [65].

Cluster #1 is an accelerometer, which incorporates 145 articles from 1992 to the present, encompassing hot words such as accelerometer, GPS, feeding behaviour, grazing, and sheep. Accelerometers, as sensors that utilize the interaction between mass and spring, are capable of sensing acceleration and translating it into a functional output signal. Accelerometers have been prevalent in multiple research and industrial testing domains, such as controlling aircraft attitudes and detecting vehicle collisions. Within the field of PLF, accelerometers are among the most applied technologies and are primarily utilized for observing animal behavior [66]. Accelerometers, as a method for monitoring individual animal welfare, overcome manual challenges related to time, resources, and discrete sampling [67]. They can collect a wealth of behavioral information from animals, which can be used in combination with machine learning algorithms to classify and identify their behavior [68,69]. Yang et al. utilized two machine learning models, K-Nearest Neighbor (KNN) and Support Vector Machine (SVM), to analyze the data collected from accelerometers on broilers, achieving the classification of specific broiler behaviors [68]. Mei et al. validated the usefulness of utilizing 3D accelerometers and machine learning models for the identification of aflatoxicosis in broiler chickens [70]. Williams and Zhan found that the data from tail-mounted accelerometers showed high classification performance for standing and lying postures of dairy cows but performed poorly in classifying excretory events [71].

Cluster #2 is an automatic milking system, which incorporates 122 articles from 1992 to the present, encompassing hot words such as automatic milking system, mastitis, robotic milking, somatic cell count, and automatic milking. The automatic milking system can more accurately monitor the physiological and behavioral changes of dairy cows, quickly identifying and addressing health issues to ensure milk quality [72,73]. One important responsibility of automatic milking systems is to detect mastitis. Bausewein et al. assessed the accuracy rate of automatic milking systems in identifying clinical mastitis in Bavarian dairy herds in southern Germany [74]. Aerts et al. studied the effect of specific factors on milking efficiency using the decision tree technique and suggested that the year of automatic milking system operation, number of lactations, calving season, age at first calving and days in milk were related to milking efficiency [75]. As cows may be stressed during a transition to automated milking systems, which could alter their health and production performance, Morales-Pineyrua et al. investigated the connection between cow temperament, behavior, and production parameters, concluding that cow temperament affects behavior and production parameters [76].

Cluster #3 is lameness, which incorporates 109 articles from 1992 to the present, encompassing hot words such as lameness, locomotion score, heat stress, animal welfare, and locomotion. Lameness is a highly expensive disease for farm animals, particularly dairy cows, resulting in significant welfare and economic stress on the livestock industry [77,78]. Kang et al. proposed a spatiotemporal network comprising video downscaling and deep learning algorithms to detect cow lameness effectively, enhancing the accuracy of lameness detection in dairy cow walking videos [79]. Jiang et al. proposed a method for early detection of cow lameness combining machine vision techniques and deep learning algorithms, which can correctly detect lameness in cows and provide an innovative means of detecting lameness in cows [80]. Zheng et al. proposed a fused attention mechanism with a conjoined attention model to automatically track and monitor cattle legs in large-scale farms, providing an effective method for accurate tracking and lameness detection of cattle legs [81].

Cluster #4 is estrus detection, which incorporates 105 articles from 1992 to the present, encompassing hot words such as estrus detection, estrus, machine learning, reproductive performance, and ovulation. By monitoring and analyzing behavior patterns, such as calls and movements, we can identify whether livestock are in estrus and determine the best breeding time to enhance reproductive efficiency [82,83]. Higaki et al. employed sensors attached to the tail to collect surface temperature data of the ventral tail base of cattle during the estrous cycle and constructed an estrus detection model using machine learning technology for training data, which was tested for estrus detection ability [84]. Wang et al. developed a lightweight sow estrus detection approach based on acoustic data and a deep convolution neural network algorithm, which analyzed short- and long-frequency sow estrus sounds, providing an effective and accurate estrus monitoring and early warning system for pig farms [85]. Yu et al. proposed an ewe estrus recognition method based on a multi-target detection layer neural network, which can accurately and promptly recognize ewe estrus behavior in large-scale mutton sheep breeding, avoiding the stress caused by contact sensor detection [86].

Cluster #5 is electronic identification, which incorporates 53 articles from 1993 to the present, encompassing hot words such as electronic identification, feeding behavior, transponder, traceability, and performance. An electronic identification (EID) system is a crucial element in the environment of PLF farms and the only technology currently mandatory under EU laws [21]. EID primarily involves the use of radio frequency identification tags (RFID) and can be primarily classified into three types: ear tags, boluses and injectable glass tags [21,66]. Ear tags offer simplicity, and low cost, and have the widest range of applications. Ruminal boluses are slightly more expensive, but are highly durable with a low loss rate, making them a popular choice in commercial farming operations. Injectable glass tags boast high reliability and safety but are limited in their use on farms due to the difficulty of removing them at the slaughterhouse. It has been demonstrated by Garcia et al. that the use of transponders is feasible for the EID of water buffaloes, and they recommended that electronic transponders be implanted in calves that are up to two months of age, as this reduces the physical rate of transponder loss and the loss of functionality [87]. Kandemir et al. employed electronic leg tags (ELT) and electronic ear tags (EET) to identify goats and concluded that the traceability of ear-tagged animals was inferior [88]. Non-invasive biometric identification could greatly enhance animal welfare and management in livestock farming. Shojaeipour et al. proposed the two-stage YOLOv3-ResNet50 algorithm, which demonstrated outstanding performance in detecting the mouth and nose area of cattle, rendering it appropriate for automated cattle biometric identification systems [89].

#### 4.2.3. Research Frontiers

**Figure 8:**
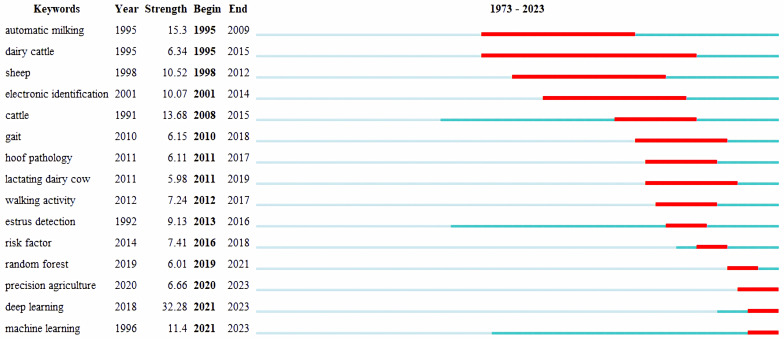
Top 15 keywords with the strongest citation bursts.

A keyword burst denotes a sudden surge in the frequency of keywords in a short duration, and analyzing keyword bursts can reveal the shift in research hotspots over different periods, and identify potential development trends and cutting-edge research [90]. Figure 8 presents the top 15 keywords of citation bursts, where “Year” indicates the year of the initial occurrence of the keyword, “Strength” implies the intensity of the burst for the keyword, and “Begin” and “End” designate the start and end of the keyword bursts, respectively. The dark blue section represents the timespan of the keyword’s appearance, while the red part represents the span of the keyword’s burst.

The burst of the keyword in PLF research mainly occurred after 1995. As for the impact cycle, “dairy cattle” had the longest duration of burst. Given the high economic value of dairy cattle and their important role in human nutrition and health, coupled with their susceptibility to diseases such as mastitis resulting in significant economic losses and animal welfare issues, the research of PLF technology in the dairy cattle industry has always been a hot topic [25,91]. Regarding the strength of keyword bursts, “deep learning” ranks first. In PLF, a large amount of monitoring data requires processing, and deep learning models can handle massive data, quickly and accurately detecting animal behaviors and anomalies, automatically diagnosing animal diseases, predicting potential diseases, and more [92,93]. The next term is “automatic milking”, the automatic milking system provides a detailed description of the milking process for each cow, recording many parameters of the milking process, which can help improve milk yield and quality [94]. The most recent bursts of keywords suggest the future direction of research. “Precision agriculture”, “deep learning” and “machine learning” are currently burst keywords. Precision agriculture (PA) is a management strategy that collects, processes, and analyzes relevant data to support management decisions in order to improve the sustainability of agricultural production [95]. PA can be divided into precision crop farming and PLF [96]. Most PA research has focused on intensive planting systems, and a study has shown that the utilization of PA in livestock management is limited in certain regions, indicating the restricted application of PLF [97]. Future research should prioritize the need for farmers to acquire more knowledge regarding PLF. To utilize vast amounts of data, machine learning has become an indispensable part of modern animal husbandry [98]. Machine learning primarily serves to monitor animal behavior and estimate economic balances for producers accurately [99,100]. Commonly used machine learning methods, such as artificial neural networks (ANN), random forests (RF), and support vector machines (SVM), are mostly used to extract or label livestock features (body weight, estrus events, growth performance) and to determine or predict relevant features [62,101,102]. Deep learning is a subdivision of machine learning that employs elaborate algorithms to detect high-level features from data facilitating better performance in image processing and classification problems, surpassing traditional machine learning [62,103]. Recently, the PLF field has shown extensive interest in deep learning-based livestock identification and localization [62,104,105]. Future research should further improve the models and networks for both machine learning and deep learning, modify the set of technologies to adapt to these two techniques, and develop automated systems for livestock tracking and health monitoring [100,103].

### 4.3. Analysis of Hot Topics

Highly cited articles are defined as those with excellent contributions to a specific research area and widely accepted research conclusions [106]. Analyzing these articles can help explore the knowledge base of PLF research and identify the current hot topics based on existing foundations [107]. To better display and enlighten readers of further research endeavors, we have collected and analyzed the top 10 highly cited articles in the field of PLF from the current quinquennial period that spans from 2019 to 2023 (Table 6). It can be seen that the main themes of PLF research are social science in PLF, the environmental impact of PLF, information technology in PLF, and animal welfare in PLF. A list of PLF article titles for the period 2019–2023 can be found in the Supplementary Material Table S2.

#### 4.3.1. Social Science in PLF

Despite the abundance of research exploring PLF from a natural science perspective, social science literature investigating the social, economic, and institutional approaches to PLF has steadily increased as well [108]. These studies can be divided into five topics: (1) Adoption of PLF on farms, which covers factors influencing technology adoption [109,110], farmers’ attitudes towards technology [111,112,113], public perceptions of technology [114,115], experiences with PLF on farms [116,117] and the roles of different players in supporting PLF [118,119]. Comprehensive consideration of various factors can promote the implementation of PLF technology on farms. (2) Effects of PLF on farmer identity, farmer skills and farm work. PLF replaces regular physical work but introduces new tasks such as equipment maintenance, monitoring, and data interpretation, resulting in an increased mental workload [31]. How PLF impacts animal management, demanding different knowledge and skills among farmers, and better farmer advisory services are needed to facilitate optimal farm system adaptation [108,120]. Farmers, livestock, and PLF technologies should work together to achieve the coevolution of all elements [121]. (3) Ethical concerns in PLF. The use of precision technologies may objectify animals into mere data points, ignoring their complex emotional and social needs, and lead to a loss of connection between farmers and animals, which is detrimental to animal well-being [33,122]. Animal welfare issues could harm production and result in lower profitability, which raises economic sustainability issues [123]. (4) Economics and management in PLF, with studies exploring the costs and benefits of PLF technology [124,125] and investment decisions for PLF technology [126,127]. (5) Transformation of PLF. Recently, scholars tend to emphasize the element of real-time monitoring and control in their definitions of PLF [20,66]. This reflects the digital transformation of PLF, as the digitization of animal husbandry can achieve real-time monitoring and control over the entire production process through intelligent equipment and sensors [33]. The transformation of PLF can be divided into two stages: the first stage focuses on digitalization, while the second stage further distinguishes between DLF and SLF.

#### 4.3.2. The Environmental Impact of PLF

The expansion of livestock farming has had numerous adverse effects on the environment, despite its contribution to increased livestock product supply. These impacts primarily manifest in two aspects. Firstly, water quality is compromised due to the excessive nutrients present in animal diets that are not fully absorbed, resulting in high levels of compounds in animal manure. Consequently, these substances gradually infiltrate the soil, leading to water pollution. Secondly, air pollution occurs through the processes of animal gastrointestinal fermentation and manure disposal, resulting in significant greenhouse gas emissions. Relevant studies indicate that approximately 15% of global emissions are attributed to animal husbandry, exerting a severe impact on air quality [128]. Therefore, the livestock industry must explore effective strategies to mitigate environmental risks while maintaining high production levels [27].

PLF aims to continuously monitor animal health and welfare in real-time. Reducing environmental pollution from animal husbandry is not its primary objective; however, it provides a valuable tool for mitigating the impact of animal husbandry on the environment [20]. Rather than relying on specialized technologies to directly reduce environmental pollution, PLF employs information technology to optimize management and minimize adverse environmental effects. Precision feeding systems, for instance, can optimize feed ratios to accurately meet the nutritional requirements of each animal, thus reducing the excretion of nutrients resulting from poor absorption and improving environmental quality [129]. Animals experiencing health and stress issues may lead to unnecessary gas emissions, but the timely identification of conditions such as lameness and mastitis through PLF technologies can help mitigate environmental pollution from animal husbandry. Furthermore, achieving high levels of livestock fertility can reduce greenhouse gas emissions by over 20% [130]. Some scholars have developed sensors, algorithms, and smart devices capable of detecting the optimal time for fertilization in animals, thereby enhancing conception rates and mitigating the greenhouse effect [129,131].

#### 4.3.3. Information Technology in FLF

Utilizing modern information technology to address deficiencies and issues in traditional animal husbandry, exploring advanced models of animal husbandry development, and stimulating internal growth potential are crucial for promoting stable and sustainable animal husbandry development [132]. Recently, supported by “Internet+”, the innovation of the new generation of information technology represented by the Internet of things, blockchain, and machine learning has been promoted in animal husbandry.

The Internet of things (IoT) is an information carrier based on the Internet, traditional telecommunications networks, etc. IoT allows for communication between farm sensors, devices, and equipment [17]. For example, with the assistance of IoT, sensors and wireless communication technology can be embedded in wearable devices for collecting massive animal data [132]. IoT enables real-time capture, collection, and transmission of livestock data, allowing for tracking, intelligent identification, and efficient monitoring and management [55,133,134]. Additionally, the application of IoT involves a substantial amount of animal and environmental data, thus raising concerns about data privacy and security [135]. Ensuring secure storage, transmission, and usage of data as well as protecting user privacy are critical challenges that IoT needs to address.

Blockchain is a distributed ledger technology that boasts decentralization, tamper-resistance, and transparency. It can provide a secure and reliable platform for data storage and exchange. Blockchain has become an important technology in many applications of the PA discipline [135]. In livestock systems, blockchain is being used to enhance the traceability of livestock products, supply chain monitoring and tracking, as well as data security and assurance [136,137,138]. For example, AppliFarm is a leading blockchain platform that can track livestock data in the animal production sector. This platform can be used to provide digital proof of animal welfare and livestock grazing [135].

Machine learning (ML) is a branch of artificial intelligence, which uses data and algorithms to imitate human learning methods, so as to gradually improve the accuracy in application [98]. ML has become an indispensable technology in modern livestock farming and has been widely used in improving animal welfare and raising animal productivity [98,99,139]. On the one hand, animal welfare involves the health and well-being of animals and is closely related to product quality, and its evaluation indicators include physiological stress indicators and behavioral indicators. ML is mainly used to monitor animal behavior in order to monitor their health status and facilitate the detection of diseases at an early stage [97,140,141]. On the other hand, there are many problems with livestock production systems and ML methods are mainly applied to accurately predict and estimate relevant parameters to improve the economic efficiency of production systems [99,142,143].

#### 4.3.4. Animal Welfare in PLF

Animal welfare means the physical and mental state of an animal in relation to the conditions in which it lives and dies [144]. An animal in a good state of welfare should be consistent with being healthy, comfortable, safe, well-fed, able to express normal patterns of behaviour, and free of unpleasant states such as pain, fear, and stress [144]. Animal welfare deals with the physical and mental state of animals and is a broad term that covers all aspects of coping with the environment and takes into account a wider range of feelings than those affecting health, and therefore, animal health is included in animal welfare as a significant part of animal welfare [145].

In PLF, the use of sensors, coupled with algorithms that combine images, sounds, movements and vital signs of animals, enables non-invasive monitoring of animals, which can improve animal welfare by detecting diseases at an early stage [146,147,148]. Animal sounds contain vital information on animal health and behavior. To address the scarcity of multi-species vocal classification algorithms, Bishop et al. proposed a multifunctional animal vocal algorithm using specific audio feature extraction techniques and machine learning models, which laid the foundation for the development of subsequent automatic animal vocal detection systems [149]. Mao et al. developed a chicken call signal recognition device based on a convolutional neural network model, which effectively avoided the problem of inefficient reliance on manual recognition [150]. Changes in animal behavior are powerful indicators of health and welfare problems, and automatic identification of animal behavior can provide a powerful tool for improving farm management and ensuring animal welfare [151,152]. Lameness is a common problem in dairy farms, and since continuous monitoring of cow lameness is too time-consuming, Warner et al. used a machine learning approach based on decision tree induction to detect the level of lameness risk in dairy herds [140].

## 5. Conclusions and Insights

### 5.1. Conclusions

This study analyzed 3658 articles on PLF research in the Web of Science database from 1973 to 2023 quantitatively using CiteSpace software. By examining the main characteristics, research cores and hot topics of PLF-related research, the following conclusions were drawn:

(1) From the perspective of the characteristics of publishing, the number of research papers on topics related to PLF generally shows an increasing trend. The international cooperation of this research is strong, and the developed countries of livestock farming in Europe and America have a large number of papers and close cooperation among countries; the research institutions of this study are mainly universities, involving agriculture-related institutions in individual countries, and the Inter-institutional cooperation network is relatively loose but the group characteristics are obvious; Daniel Berckmans and his team have published the most articles, and the overall cooperation among scholars is characterized by “small concentration and large dispersion”, and the cooperation among scholars is weak; the research belongs to a multidisciplinary cross-fertilization research field, mainly including animal science, veterinary science, computer science, agricultural engineering and environmental science.
(2) Research hotspots in PLF include precision dairy technology, precision cattle technology, intelligent systems, and animal behavior research. The hot words can be categorized into PLF technology, technology application objects, and technology use, with research directions focused on deep learning, accelerometer, automatic milking systems, lameness, estrus detection, and electronic identification, and the specific research contents intersecting. Scholars have paid more attention to deep learning and machine learning since 2021.
(3) From the perspective of hot topics, the research on PLF mainly includes four hot topics: social science, environmental impact, information technology, and animal welfare. The literature on PLF from a social science perspective can be divided into five categories: adoption of PLF on farms, effects of PLF on farmer identity, farmer work and farm work, ethical concerns in PLF, economics and management of PLF, and transformation of PLF. PLF provides a valuable tool for mitigating the impact of animal husbandry on the environment by optimizing livestock management. The new generation of information technology represented by the Internet of Things, blockchain, and machine learning plays an important role in promoting the stable and sustainable development of animal husbandry. The combination of sensors and algorithms can effectively extract and analyze the images, sounds, movements and vital signs of animals, facilitating early detection of diseases and improving animal welfare.

### 5.2. Insights

PLF establishes a scientific management system and decision support system through information acquisition, processing, understanding and application, which can promote the low-cost, high-efficiency and safe development of increasingly large-scale livestock farming and realize the modernization of livestock farming. Future research on PLF can be deepened from the following three aspects:

(1) Strengthen the exchange and cooperation of PLF research. Universities with outstanding contributions in the field of PLF, such as Wageningen University and Research, University of Guelph and Katholieke Universiteit Leuven, should actively hold academic exchange conferences or exchange programs, and other institutions should actively establish friendly exchange relations with these universities and carry out related project cooperation. Scholars should actively participate in international exchange meetings and forums on PLF, discuss and learn the research experience and latest achievements of PLF with scholars from all over the world, and strengthen extensive exchanges and cooperation among scholars.
(2) Pay attention to the combination of multi-disciplines and multi-methods. The research content needs the intervention of many disciplines and fields such as animal science, veterinary science, computer science, agricultural engineering and environmental science; in terms of research methods, it is necessary to organically integrate various methods such as big data analysis and model analysis, and strengthen the innovative integration of information technology in animal husbandry, in order to promote the research and development of PLF.
(3) Strengthen the application of deep learning, machine learning, and other technologies. Develop integrated intelligent detection channels that integrate lameness recognition, body condition scoring, weight estimation, respiratory heart rate sign measurement and other multi-functional features; give full play to the role of data decision support, and realize refinement management to improve animal welfare. In addition, it is necessary to consider the relationship between PLF and humans and animals from the perspective of social science and to guide farmers to change the concept of responsibility to deeply explore and give full play to the value of PLF.
(4) Strengthen the focus and exploration of DLF. PLF is gradually transforming into DLF, and farmers, scientists and consumers as well as other stakeholders should consciously participate in and pay attention to following this process in order to accelerate the innovative integration of digital technology and animal husbandry and promote the development of animal husbandry.

There are also some shortcomings in this study: Firstly, we only included the relevant papers contained in the Web of Science database as the research object and directly used parameters such as citation frequency provided by Web of Science as our analysis indicators, which cannot fully represent the research results in the field of PLF. Further research in the later period can increase the related data of other academic retrieval databases; secondly, this study primarily considered the commonly used information technology in PLF when setting the search type, and to ensure simplicity, certain search terms with minimal results were removed. Inevitably some literature was overlooked. However, the research results of this study still have important representative significance and can continue to improve the setting of search types in the future.

## Figures and Tables

**Table 1:** Top 20 countries according to the number of publications.

No	Country	Year	Papers	Centrality
1	USA	1973	698	0.24
2	China	2000	419	0.09
3	UK	1992	325	0.22
4	Australia	2000	301	0.13
5	Germany	1990	300	0.11
6	Canada	1997	270	0.10
7	Netherlands	1992	237	0.18
8	Italy	1998	202	0.07
9	Belgium	2001	196	0.04
10	Brazil	2003	193	0.03
11	France	1986	140	0.18
12	Spain	1999	131	0.23
13	Denmark	1996	110	0.01
14	Switzerland	2003	102	0.02
15	Japan	2005	95	0.02
16	New Zealand	1996	89	0.03
17	Republic of Ireland	2001	80	0.01
18	South Korea	2009	79	0.06
19	India	1998	76	0.03
20	Austria	2004	72	0.01

**Table 2:** Top 20 institutions according to the number of publications.

No	Institution	Affiliated Country	Year	Papers	Centrality
1	Wageningen University and Research	Netherlands	1996	143	0.17
2	Katholieke Univ Leuven	Belgium	2004	117	0.07
3	University Guelph	Canada	2007	99	0.12
4	China Agricultural University	China	2013	87	0.07
5	Univ Sydney	Australia	2012	65	0.03
6	USDA	USA	1973	64	0.08
7	University of Milan	Italy	2008	64	0.04
8	Univ British Columbia	Canada	2009	63	0.02
9	Agr and Agri Food Canada	Canada	1998	60	0.04
10	Univ Wisconsin	USA	2010	59	0.03
11	Aarhus University	Denmark	2008	58	0.08
12	INRAE	France	1986	53	0.09
13	Univ New England	Australia	2000	51	0.06
14	Ghent University	Belgium	2001	48	0.02
15	Iowa State University	USA	1999	45	0.03
16	Agr Res Org	Israel	1997	40	0.03
17	Univ Kentucky	USA	2000	40	0.02
18	Swedish Univ Agr Sci	Sweden	2001	34	0.04
19	Northwest A&F Univ	China	2018	32	0.01
20	Univ Bern	Switzerland	2016	31	0.01

**Table 3:** Top 10 prolific authors according to the number of publications.

No	Author	Affiliated Institutions	Year	Records	Centrality
1	Daniel Berckmans	Katholieke Univ Leuven	2004	67	0.02
2	Tomas Norton	Katholieke Univ Leuven	2017	36	0.01
3	Claudia Bahr	Katholieke Univ Leuven	2010	33	0.01
4	Ilan Halachmi	Agr Res Org	2000	30	0.03
5	Marcella Guarino	University of Milan	2008	28	0.02
6	Jeffrey Rushen	Univ British Columbia	2009	27	0.01
7	Trevor J. Devries	Univ Guelph	2016	26	0.00
8	Henk Hogeveen	Wageningen Univ and Res	1994	22	0.01
9	Jeffrey M. Bewley	Univ Kentucky	2001	20	0.01
10	Sergio C. Garcia	Univ Sydney	2014	19	0.00

**Table 4:** Top 20 keywords according to the number of publications.

No	Keywords	Year	Papers	Centrality
1	dairy cow	1992	530	0.10
2	cattle	1991	526	0.13
3	behavior	1997	491	0.08
4	system	1996	457	0.15
5	performance	1993	291	0.07
6	cow	1992	279	0.08
7	machine learning	1996	279	0.03
8	precision livestock farming	2008	271	0.01
9	dairy cattle	1995	216	0.07
10	health	1999	198	0.03
11	time	2001	194	0.02
12	deep learning	2018	184	0.01
13	management	2004	179	0.03
14	classification	2000	176	0.03
15	animal welfare	1997	170	0.06
16	feeding behavior	1997	153	0.04
17	model	2004	136	0.03
18	computer vision	1995	131	0.01
19	milk yield	1999	125	0.02
20	welfare	2007	125	0.02

**Table 5:** Keywords clustering information table.

Cluster Serial Number	Cluster Name	Document Number	Keywords (Logarithmic Likelihood Ratio, *p*-Value)
#0	deep learning	168	deep learning (174.71, 1.0 × 10^−4^); computer vision (118.87, 1.0 × 10^−4^); image processing (68.37, 1.0 × 10^−4^); precision livestock farming (58.38, 1.0 × 10^−4^); object detection (53.56, 1.0 × 10^−4^)
#1	accelerometer	145	accelerometer (36.26, 1.0 × 10^−4^); gps (35.94, 1.0 × 10^−4^); feeding behaviour (30.08, 1.0 × 10^−4^); grazing (29.56, 1.0 × 10^−4^); sheep (28.9, 1.0 × 10^−4^)
#2	automatic milking system	122	automatic milking system (57.92, 1.0 × 10^−4^); mastitis (47.72, 1.0 × 10^−4^); robotic milking (46.23, 1.0 × 10^−4^); somatic cell count (40.29, 1.0 × 10^−4^); automatic milking (46.98, 1.0 × 10^−4^)
#3	lameness	109	lameness (126.76, 1.0 × 10^−4^); locomotion score (40.18, 1.0 × 10^−4^); heat stress (39.21, 1.0 × 10^−4^); animal welfare (37.95, 1.0 × 10^−4^); locomotion (33.11, 1.0 × 10^−4^)
#4	estrus detection	105	estrus detection (56.1, 1.0 × 10^−4^); estrus (55.48, 1.0 × 10^−4^); machine learning (32.73, 1.0 × 10^−4^); reproductive performance (31.18, 1.0 × 10^−4^); ovulation (28.8, 1.0 × 10^−4^)
#5	electronic identification	53	electronic identification (42.82, 1.0 × 10^−4^); feeding behavior (36.53, 1.0 × 10^−4^); transponder (30.98, 1.0 × 10^−4^); traceability (25.81, 1.0 × 10^−4^); performance (15.7, 1.0 × 10^−4^)

**Table 6:** Top 10 Highly cited articles from 2019 to 2023 as determined using Web of Science data.

First Author	Title	Journal	Year	Cited Frequency
Laurens Klerkx	A review of social science on digital agriculture, smart farming and agriculture 4.0: New contributions and a future research agenda	NJAS-Wageningen Journal of Life Sciences	2019	344
Emanuela Tullo	Review: Environmental impact of livestock farming and Precision Livestock Farming as a mitigation strategy	Science of the Total Environment	2019	137
Mohamed Torky	Integrating blockchain and the internet of things in precision agriculture: Analysis, opportunities, and challenges	Computers and Electronics in Agriculture	2020	98
Lefteris Benos	Machine Learning in Agriculture: A Comprehensive Updated Review	Sensors	2021	95
Ricardo S. Alonso	An intelligent Edge-IoT platform for monitoring livestock and crops in a dairy farming scenario	Ad Hoc Networks	2020	91
Ilan Halachmi	Smart Animal Agriculture: Application of Real-Time Sensors to Improve Animal Well-Being and Production	Annual Review of Animal Biosciences	2019	91
Abhinav Sharma	Machine Learning Applications for Precision Agriculture: A Comprehensive Review	Sensors	2020	86
Qiao Yongliang	Cattle segmentation and contour extraction based on Mask R-CNN for precision livestock farming	Computers and Electronics in Agriculture	2019	80
Callum Eastwood	Making sense in the cloud: Farm advisory services in a smart farming future	NJAS-Wageningen Journal of Life Sciences	2019	70
Madonna Benjamin	Precision Livestock Farming in Swine Welfare: A Review for Swine Practitioners	Animals	2019	67

## Data Availability

All data generated or analyzed during this study are included in this published article.

## Supplementary Materials

The following supporting information can be downloaded at: https://www.mdpi.com/article/10.3390/ani13132096/s1, Table S1: Top 20 institutions according to the number of publications during 2019–2023; Table S2. Summary of PLF article titles for the period 2019–2023.
