# Supplementary material for: Precision Livestock Farming Research: A Global Scientometric Review
Source: Animals (Basel). 2023 Jun 24;13(13):2096. doi: 10.3390/ani13132096 (PMC10340063; doi:10.3390/ani13132096)
Supplement: Supplementary file 1 [file animals-13-02096-s001.zip › animals-2433179-supplementary.pdf]

Table S1. Top 20 institutions according to the number of publications during 2019-2023.

No	Institution	Afliated Country	Papers	Centrality
1	Wageningen University & Research	Netherlands	79	0.25
2	China Agricultural University	China	77	0.24
3	Univ Sydney	Australia	42	0.11
4	Univ Guelph	Canada	38	0.16
5	Katholieke Univ Leuven	Belgium	38	0.17
6	Univ Wisconsin	USA	37	0.09
7	Aarhus University	Denmark	32	0.08
8	Northwest A&F Univ	China	29	0.04
9	Univ New England	Australia	26	0.09
10	Minist Agr & Rural Affairs	China	26	0.06
11	Univ Milan	Italy	26	0.01
12	Massey Univ	New Zealand	22	0.1
13	Univ Ghent	Belgium	22	0.05
14	Univ Bern	Switzerland	22	0.03
15	Univ Florida	USA	21	0.09
16	Swedish Univ Agr Sci	Sweden	21	0.08
17	Univ Melbourne	Australia	20	0.07
18	Univ Alberta	Canada	20	0.07
19	Univ British Columbia	Canada	19	0.01
20	Univ Nottingham	UK	19	0.03

Table S2. Summary of PLF article titles for the period 2019-2023.

A 4 mu W Low-Power Audio Processor System for Real-Time Jaw Movements Recognition in Grazing Cattle
A bilateral symmetry based pose normalization framework applied to livestock body measurement in point clouds
A biological integrity framework for describing animal welfare and wellbeing
A Cascaded Model Based on EfficientDet and YOLACT plus plus for Instance Segmentation of Cow Collar ID Tag in an Image
A Case Study Using Accelerometers to Identify Illness in Ewes following Unintentional Exposure to Mold-Contaminated Feed
A CNN-based methodology for cow heat analysis from endoscopic images
A Combined Offline and Online Algorithm for Real-Time and Long-Term Classification of Sheep Behaviour: Novel Approach for Precision Livestock Farming
A Comparative Study of Machine Learning Methods for Predicting Live Weight of Duroc, Landrace, and Yorkshire Pigs
A Comparison between the Egg Yolk Flavor of Indigenous 2 Breeds and Commercial Laying Hens Based on Sensory Evaluation, Artificial Sensors, and GC-MS
A comparison of indoor and outdoor calf housing systems using automated and manual feeding methods and their effect on calf health, behavior, growth, and labor

A comparison of logistic regression and classification tree to assess brucellosis associated risk factors in dairy cattle
A comparison of machine learning and logistic regression in modelling the association of body condition score and submission rate
A comparison of the value of two machine learning predictive models to support bovine tuberculosis disease control in England
A computer vision approach based on deep learning for the detection of dairy cows in free stall barn
A computer vision approach for recognition of the engagement of pigs with different enrichment objects
A computer vision approach to improving cattle digestive health by the monitoring of faecal samples
A computer vision-based method for spatial-temporal action recognition of tail-biting behaviour in group-housed pigs
A Conceptual Approach towards Surveying German Dairy Farmers' Acceptance of Digitization in Dairy Production
A cost-effective on-site milk analyzer based on multispectral sensor
A Data Augmentation Method for Cow Behavior Estimation Systems Using 3-Axis Acceleration Data and Neural Network Technology
A data-driven approach to increasing the lifetime of IoT sensor nodes
A Data-driven Horizon Scan of Bacterial Pathogens at the Wildlife-livestock Interface
A Data-Driven Prediction Method for an Early Warning of Coccidiosis in Intensive Livestock Systems: A Preliminary Study
A data-driven prediction of lifetime resilience of dairy cows using commercial sensor data collected during first lactation
A Dead Broiler Inspection System for Large-Scale Breeding Farms Based on Deep Learning
A decision-support tool for investment analysis of automated oestrus detection technologies in a seasonal dairy production system
A deep learning algorithm predicts milk yield and production stage of dairy cows utilizing ultrasound echotexture analysis of the mammary gland
A Deep Learning Model for Detecting Cage-Free Hens on the Litter Floor
A deep learning-based approach for feeding behavior recognition of weanling pigs
A deep learning-based cow behavior recognition scheme for improving cattle behavior modeling in smart farming
A defencing algorithm based on deep learning improves the detection accuracy of caged chickens
A field study using different technologies to detect calving at a large-scale hungarian dairy farm
A framework for modelling, control and supervision of poultry farming
A Framework of an Integrated Livestock Vehicle Trajectory Database Using Digital Tachograph Data
A gene prioritization method based on a swine multi-omics knowledgebase and a deep

learning model
A generalised additive model to characterise dairy cows' responses to heat stress
A genome-wide association study for eumelanin pigmentation in chicken plumage using a computer vision approach
A Global Review of Monitoring, Modeling, and Analyses of Water Demand in Dairy Farming
A Heuristic and Data Mining Model for Predicting Broiler House Environment Suitability
A High-Performance Day-Age Classification and Detection Model for Chick Based on Attention Encoder and Convolutional Neural Network
A High-Precision Method for 100-Day-Old Classification of Chickens in Edge Computing Scenarios Based on Federated Computing
A Hybrid Model for Temperature Prediction in a Sheep House
A Life Cycle Framework of Green IoT-Based Agriculture and Its Finance, Operation, and Management Issues
A light-weight and accurate pig detection method based on complex scenes
A lightweight CNN-based model for early warning in sow oestrus sound monitoring
A lightweight deep learning model for cattle face recognition
A lightweight learning-based decoding algorithm for intraneural vagus nerve activity classification in pigs
A Lightweight Neural Network-Based Method for Detecting Estrus Behavior in Ewes
A Low-Cost IoT-Based System to Monitor the Location of a Whole Herd
A low-cost, automated parasite diagnostic system via a portable, robotic microscope and deep learning
A machine learning approach for modelling the occurrence of Galba truncatula as the major intermediate host for Fasciola hepatica in Switzerland
A machine learning approach for the identification of population-informative markers from high-throughput genotyping data: application to several pig breeds
A machine learning based decision aid for lameness in dairy herds using farm-based records
A Machine Learning Framework Based on Extreme Gradient Boosting to Predict the Occurrence and Development of Infectious Diseases in Laying Hen Farms, Taking H9N2 as an Example
A machine learning framework to predict the next month's daily milk yield, milk composition and milking frequency for cows in a robotic dairy farm
A machine vision system for early detection and prediction of sick birds: A broiler chicken model
A machine vision system to detect and count laying hens in battery cages
A machine vision system to predict individual cow feed intake of different feeds in a cowshed
A Machine Vision-Based Method for Monitoring Broiler Chicken Floor Distribution
A Machine Vision-Based Method for Monitoring Scene-Interactive Behaviors of Dairy Calf

A Machine Vision-Based Method Optimized for Restoring Broiler Chicken Images Occluded by Feeding and Drinking Equipment
A near-zero energy system based on a kinetic energy harvester for smart ranch
A Non-Contact Cow Estrus Monitoring Method Based on the Thermal Infrared Images of Cows
A Non-Invasive Millimetre-Wave Radar Sensor for Automated Behavioural Tracking in Precision Farming-Application to Sheep Husbandry
A novel accelerometry approach combining information on classified behaviors and quantified physical activity for assessing health status of cattle: a preliminary study
A novel automated system to acquire biometric and morphological measurements and predict body weight of pigs via 3D computer vision
A Novel Combined Model for Predicting Humidity in Sheep Housing Facilities
A novel in vivo 433 MHz radio channel indoor study targeting on power saving for ruminal health monitoring boluses
A novel method to detect bovine sex pheromones using L-tyrosine-capped silver nanoparticles: Special reference to nanosensor based estrus detection
A novel modelling approach to quantify the response of dairy goats to a high-concentrate diet
A novel multimodal framework for automatic recognition of individual cattle based on hybrid features using sparse stacked denoising autoencoder and group sparse representation techniques
A novel PIG and an intelligent pigging scheme based on Deep-learning technology?
A novel protocol to measure startle magnitude in sheep
A novel regularized approach for functional data clustering: an application to milking kinetics in dairy goats
A parsimonious software sensor for estimating the individual dynamic pattern of methane emissions from cattle
A PCA-based frame selection method for applying CNN and LSTM to classify postural behaviour in sows
A pilot study on assessment of locomotor behavior using a video tracking system in minipigs
A Pilot Study Using Accelerometers to Characterise the Licking Behaviour of Penned Cattle at a Mineral Block Supplement
A Portable Smartphone-Based System for the Detection of Blood Calcium Using Ratiometric Fluorescent Probes
A prediction method for transport stress in meat sheep based on GA-BPNN
A preliminary study of the physiological and behavioral response of beef cattle to unmanned aerial vehicles (UAVs)
A probabilistic framework for behavioral identification from animal-borne accelerometers
A Probabilistic Structural Equation Model to Evaluate Links between Gut Microbiota and Body Weights of Chicken Fed or Not Fed Insect Larvae
A promising approach towards precise animal weight monitoring using convolutional

neural networks
A Range-Based Algorithm for Autonomous Navigation of an Aerial Drone to Approach and Follow a Herd of Cattle
A Real-Time Automated System for Monitoring Individual Feed Intake and Body Weight of Group-Housed Young Chickens
A Recognition Method of Ewe Estrus Crawling Behavior Based on Multi-Target Detection Layer Neural Network
A Retrospective Case Study into the Effect of Hoof Lesions on the Lying Behaviour of Holstein-Friesian in a Loose-Housed System
A Review in the Automatic Detection of Pigs Behavior with Sensors
A review of deep learning algorithms for computer vision systems in livestock
A review of measuring, assessing and mitigating heat stress in dairy cattle
A REVIEW OF PASSIVE RADIO FREQUENCY IDENTIFICATION SYSTEMS FOR ANIMAL MONITORING IN LIVESTOCK FACILITIES
A review of social science on digital agriculture, smart farming and
A review of traditional and machine learning methods applied to animal breeding
A review of video-based pig behavior recognition
A Review of Welfare Indicators of Indoor-Housed Dairy Cow as a Basis for Integrated Automatic Welfare Assessment Systems
A Review on Computer Vision Technology for Monitoring Poultry Farm-Application, Hardware, and Software
A review on dairy cattle farming: Is precision livestock farming the compromise for an environmental, economic and social sustainable production
A review on the woody breast condition, detection methods, and product utilization in the contemporary poultry industry
A Review: Development of Computer Vision-Based Lameness Detection for Dairy Cows and Discussion of the Practical Applications
A robust classification system for Southern Yellow cow behavior using 3-DoF accelerometers
A sensor-based solution to monitor grazing cattle drinking behaviour and water intake
A sensor-fusion-system for tracking sheep location and behaviour
A Simultaneous Pipe-Attribute and PIG-Pose Estimation (SPPE) Using 3-D Point Cloud in Compressible Gas Pipelines
A Smart Sensing System of Water Quality and Intake Monitoring for Livestock and Wild Animals
A Smartphone-Based Sensor With an Uncooled Infrared Thermal Camera for Accurate Temperature Measurement of Pig Groups
A spatialised information system to support decisions regarding grazing management in mountainous and Mediterranean rangelands
A Spatiotemporal Convolutional Network for Multi-Behavior Recognition of Pigs
A Stacking Ensemble Learning Framework for Genomic Prediction
A Study on the Detection of Cattle in UAV Images Using Deep Learning
A supervised machine learning method to detect anomalous real-time broiler breeder

body weight data recorded by a precision feeding system
A Survey of Dairy Cattle Behavior in Different Barns in Northern Italy
A Survey of Italian Dairy Farmers' Propensity for Precision Livestock Farming Tools
A survey of machine learning approaches in animal behaviour
A survey on sensor systems used in Italian dairy farms and comparison between performances of similar herds equipped or not equipped with sensors
A systematic literature review on deep learning applications for precision cattle farming
A Systematic Literature Review on the Use of Deep Learning in Precision Livestock Detection and Localization Using Unmanned Aerial Vehicles
A systematic literature review on the use of machine learning in precision livestock farming
A systematic review o f the potential uses of on-animal sensors to monitor the welfare of sheep evaluated using the Five Domains Model as a framework
A Systematic Review of Automatic Health Monitoring in Calves: Glimpsing the Future From Current Practice
A Systematic Review of Precision Livestock Farming in the Poultry Sector: Is Technology Focussed on Improving Bird Welfare?
A Systematic Review on Commercially Available and Validated Sensor Technologies for Welfare Assessment of Dairy Cattle
A Systematic Review on Validated Precision Livestock Farming Technologies for Pig Production and Its Potential to Assess Animal Welfare
A Technological Approach to Support Extensive Livestock Management in the Portuguese Montado Ecosystem
A Touchscreen Device for Behavioral Testing in Pigs
A Transdisciplinary Approach Supporting the Implementation of a Big Data Project in Livestock Production: An Example From the Swiss Pig Production Industry
A tristimulus-formant model for automatic recognition of call types of laying hens
A Vector Representation of Lactation Curves for Dairy Cows
A Vision for Development and Utilization of High-Throughput Phenotyping and Big Data Analytics in Livestock
A vision-based instrument for measuring milk somatic cell count
A wearable microwave instrument can detect and monitor traumatic abdominal injuries in a porcine model
Accelerometer derived rumination monitoring detects changes in behaviour around parturition
Accelerometer systems as tools for health and welfare assessment in cattle and pigs - A review
Acceptability of Artificial Intelligence in Poultry Processing and Classification Efficiencies of Different Classification Models in the Categorisation of Breast Fillet Myopathies
Accessibility to Reproductive Technologies by Low-Income Beef Farmers in South Africa
Accuracy to Predict the Onset of Calving in Dairy Farms by Using Different Precision

Livestock Farming Devices
Accurate body measurement of live cattle using three depth cameras and non-rigid 3-D shape recovery
Accurate detection of lameness in dairy cattle with computer vision: A new and individualized detection strategy based on the analysis of the supporting phase
Accurate prediction and genome-wide association analysis of digital intramuscular fat content in longissimus muscle of pigs
Acoustic features of vocalization signal in poultry health monitoring
Acoustic sensor determination of repeatable cow urinations traits in winter and spring
Activity and rumination changes as predictors of calving in primiparous and multiparous Holstein cows
Activity detection of suckling piglets based on motion area analysis using frame differences in combination with convolution neural network
Adoption of digital technologies in agriculture-an inventory in a european small-scale farming region
Adoption of Precision Technologies by Brazilian Dairy Farms: The Farmer's Perception
ADOPTION SCORES FOR BUFFALO-BASED TECHNOLOGIES IN THE PHILIPPINES AS INFLUENCED BY SOCIO-ECONOMIC, TECHNOLOGICAL, COMMUNICATION, AND INSTITUTIONAL FACTORS
Advancements in sensor technology and decision support intelligent tools to assist smart livestock farming
Advances of Computational Fluid Dynamics (CFD) applications in agricultural building modelling: Research, applications and challenges
Affective State Recognition in Livestock-Artificial Intelligence Approaches
Aggregate Farming in the Cloud: The AFarCloud ECSEL project
Agricultural robotics research applicable to poultry production: A review
Agro-Livestock Farming System Sustainability during the COVID-19 Era: A Cross-Sectional Study on the Role of Information and Communication Technologies
AI Based Digital Twin Model for Cattle Caring
AIoT-Cloud-Integrated Smart Livestock Surveillance via Assembling Deep Networks with Considering Robustness and Semantics Availability
Air temperature, carbon dioxide, and ammonia assessment inside a commercial cage layer barn with manure-drying tunnels
Algorithm development for individualized precision feeding of supplemental top dresses to influence feed efficiency of dairy cattle
Alterations in sick dairy cows' daily behavioural patterns
Alterations in vaginal temperature during the estrous cycle in dairy cows detected by a new intravaginal device-a pilot study
Alternative additives associated in the feeding of laying hens: performance, biometrics, bone traits, and economic evaluation-an unsupervised machine learning approach
Alternative measurement systems for recording cardiac activity in animals: a pilot study
Ammonia Generation System for Poultry Health Research Using Arduino
An "orientation sphere" visualization for examining animal head movements

An Absorbing Markov Chain Model to Predict Dairy Cow Calving Time
An acoustic sensor technology to detect urine excretion
An adaptive pig face recognition approach using Convolutional Neural Networks
An Advanced Chicken Face Detection Network Based on GAN and MAE
An application for the classification of egg quality and haugh unit based on characteristic egg features using machine learning models
An application of least square support vector machine model with parameters optimization for predicting body weight of Harnai sheep breed
An Approach for Autonomous Feeding Robot Path Planning in Poultry Smart Farm
An approach for goose egg recognition for robot picking based on deep learning
An Approach towards IoT-Based Predictive Service for Early Detection of Diseases in Poultry Chickens
An Approach towards Motion-Tolerant PPG-Based Algorithm for Real-Time Heart Rate Monitoring of Moving Pigs
An attempt at estrus detection in cattle by continuous measurements of ventral tail base surface temperature with supervised machine learning
An attempt of using public ambient temperature data in swine genetic evaluation for litter-size traits at birth in japan
An Attention Mechanism-Improved YOLOv7 Object Detection Algorithm for Hemp Duck Count Estimation
An automated method to quantify the composition of live pigs based on computed tomography segmentation using deep neural networks
An ear-attached accelerometer as an on-farm device to predict the onset of calving in dairy cows
An efficient anchor-free method for pig detection
An Efficient Porcine Acoustic Signal Denoising Technique Based on EEMD-ICA-WTD
An Estimate of the Effects from Precision Livestock Farming on a Productivity Index at Farm Level. Some Evidences from a Dairy Farms' Sample of Lombardy
An ethogram of biter and bitten pigs during an ear biting event: first step in the development of a Precision Livestock Farming tool
An evaluation of a novel device for measuring eating, rumination, and inactive behaviors in lactating Holstein dairy cattle
An evaluation of machine learning for genomic prediction of hairy syndrome in dairy cattle
An experimental study of stunned state detection for broiler chickens using an improved convolution neural network algorithm
An Imperative Role of Digitalization in Monitoring Cattle Health for Sustainability
An Improved Approach to Automated Measurement of Body Condition Score in Dairy Cows Using a Three-Dimensional Camera System
An Improved Chicken Swarm Optimization Algorithm and its Application in Robot Path Planning
An Improved Intelligent Control System for Temperature and Humidity in a Pig House
An Improved Single Shot Multibox Detector Method Applied in Body Condition Score

for Dairy Cows
An inexpensive and open-source method to study large terrestrial animal diet and behaviour using time-lapse video and GPS
An Information-Theoretic Approach to Detect the Associations of GPS-Tracked Heifers in Pasture
An Initial Study on the Use of Machine Learning and Radio Frequency Identification Data for Predicting Health Outcomes in Free-Range Laying Hens
An Innovative Concept for a Multivariate Plausibility Assessment of Simultaneously Recorded Data
An integrated device for rapid analysis of indoor air quality in farms: The cases of milking parlors and greenhouses for baby leaf cultivation
An intelligent Edge-IoT platform for monitoring livestock and crops in a dairy farming scenario
An intelligent method for dairy goat tracking based on Siamese network
An intelligent model for predicting the dressed weight of pigs using morphometric measurements
An intelligent monitoring system for a pig breeding environment based on a wireless sensor network
An Intelligent Pig Weights Estimate Method Based on Deep Learning in Sow Stall Environments
An Internet of Things Platform Based on Microservices and Cloud Paradigms for Livestock
An IoT Architecture for Continuous Livestock Monitoring Using LoRa LPWAN
An IoT Platform towards the Enhancement of Poultry Production Chains
An IoT-Based Design Using Accelerometers in Animal Behavior Recognition Systems
An IoT-Based Solution for Intelligent Farming
An online method for estimating grazing and rumination bouts using acoustic signals in grazing cattle
An on-site, highly specific immunosensor for Escherichia coli detection in field milk samples from mastitis-affected dairy cattle
An overview of the current trends in precision pig farming technologies
An Overview on the Use of Near Infrared Spectroscopy (NIRS) on Farms for the Management of Dairy Cows
AN RFID-BASED AUTOMATED INDIVIDUAL PERCHING MONITORING SYSTEM FOR GROUP-HOUSED POULTRY
An ultra-high frequency radio frequency identification system for studying individual feeding and drinking behaviors of group-housed broilers
An updated model of hydrocephalus in sheep to evaluate the performance of a device for ambulatory wireless monitoring of cerebral pressure through shunts
Analysing the space-usage-pattern of a cow herd using video surveillance and automated motion detection
Analysis of Accelerometer and GPS Data for Cattle Behaviour Identification and Anomalous Events Detection

Analysis of Cattle Social Transitional Behaviour: Attraction and Repulsion
Analysis of Growth Performance in Swine Based on Machine Learning
Analysis of the Factors Influencing Body Weight Variation in Hanwoo Steers Using an Automated Weighing System
Analytics in sustainable precision animal nutrition
Animal Welfare Implications of Digital Tools for Monitoring and Management of Cattle and Sheep on Pasture
Animal Welfare Management in a Digital World
Animal-related, non-invasive indicators for determining heat stress in dairy cows
Anogenital distance is associated with postpartum estrous activity, intensity of estrous expression, ovulation, and progesterone concentrations in lactating Holstein cows
Anomaly Detection of Operating Equipment in Livestock Farms Using Deep Learning Techniques
Antimicrobial use and farmers' attitude toward mastitis treatment on dairy farms with automatic or conventional milking systems
Applicability of Machine-Learned Regression Models to Estimate Internal Air Temperature and CO2 Concentration of a Pig House
Applicability of statistical and machine learning-based regression algorithms in modeling of carbon dioxide emission in experimental pig barns
Application and research progress of infrared thermography in temperature measurement of livestock and poultry animals: A review
Application note: Validation of BovHEAT-An open-source analysis tool to process data from automated activity monitoring systems in dairy cattle for estrus detection
APPLICATION OF A NOVEL GREY MODEL FOR FORECASTING INDOOR AIR TEMPERATURE IN POULTRY HOUSES: CONTROL STRATEGY
APPLICATION OF A NOVEL GREY MODEL FOR FORECASTING INDOOR AIR TEMPERATURE IN POULTRY HOUSES: MODEL DEVELOPMENT
Application of accelerometers to record drinking behaviour of beef cattle
Application of Deep Learning in Remote Sensing Monitoring of Large Herbivores- A Case Study in Qinghai Tibet Plateau
Application of deep learning in sheep behaviors recognition and influence analysis of training data characteristics on the recognition effect
Application of depth sensor to estimate body mass and morphometric assessment in Nellore heifers
Application of ensemble learning to genomic selection in chinese simmental beef cattle
Application of In-Paddock Technologies to Monitor Individual Self-Fed Supplement Intake and Liveweight in Beef Cattle
Application of Machine Learning Algorithms to Predict Body Condition Score from Liveweight Records of Mature Romney Ewes
Application of machine learning to improve dairy farm management: A systematic literature review
Application of machine-learned metadata-driven model for dairy barn ventilation simulation

Application of Methods to Assess Animal Welfare and Suffering Caused by Infectious Diseases in Cattle and Swine Populations
Application of microchip and infrared thermography for monitoring body temperature of beef cattle kept on pasture
Application of Microwave Moisture Sensor for DOC and Animal Feed
Application of MODIS NDVI for Monitoring Kenyan Rangelands Through a Web Based Decision Support Tool
Application of random forest classification to predict daily oviposition events in broiler breeders fed by precision feeding system
Applications of computer vision systems for meat safety assurance in abattoirs: A systematic review
Applications of Smart Technology as a Sustainable Strategy in Modern Swine Farming
Applying machine learning techniques on feeding behavior data for early estrus detection in dairy heifers
Are automated sensors a reliable tool to estimate behavioural activities in grazing beef cattle?
Artificial insemination for milk production in India: A statistical insight
Artificial intelligence and animal health
Artificial intelligence and IoT-based biomedical sensors for intelligent cattle husbandry systems
Artificial Intelligence Applied to a Robotic Dairy Farm to Model Milk Productivity and Quality based on Cow Data and Daily Environmental Parameters
Artificial Intelligence Best Practices in Smart Agriculture
Artificial Intelligence for Lameness Detection in Horses-A Preliminary Study
Artificial Intelligence for the Prediction of the Thermal Performance of Evaporative Cooling Systems
Artificial intelligence in animal farming: A systematic literature review
Artificial intelligence, sensors, robots, and transportation systems drive an innovative future for poultry broiler and breeder management
ARTIFICIAL NEURAL NETWORKS FOR PREDICTION OF PHYSIOLOGICAL AND PRODUCTIVE VARIABLES OF BROILERS
Artificial Neural Networks on Eggs Production Data Management
Artificial Neural Networks to Predict Egg-Production Traits in Commercial Laying Breeder Hens
ASAS-NANP SYMPOSIUM: Applications of machine learning for livestock body weight prediction from digital images
ASAS-NANP symposium: mathematical modeling in animal nutrition: limitations and potential next steps for modeling and modelers in the animal sciences
ASAS-NANP Symposium: Mathematical Modeling in Animal Nutrition: Opportunities and challenges of confined and extensive precision livestock production
ASAS-NANP SYMPOSIUM: prospects for interactive and dynamic graphics in the era of data-rich animal science
ASN-ASAS SYMPOSIUM: FUTURE OF DATA ANALYTICS IN NUTRITION:

Mathematical modeling in ruminant nutrition: approaches and paradigms, extant models, and thoughts for upcoming predictive analytics
Aspects of grazing behavior in dairy cows on a farm with an automated milking system and selection gates
Assessing animal welfare at the f arm level: do we care sufficiently about the individual?
Assessing environmental control strategies in cage-free aviary housing systems: Egg production analysis and Random Forest modeling
Assessing machine learning techniques in forecasting lumpy skin disease occurrence based on meteorological and geospatial features
Assessing optimal frequency for image acquisition in computer vision systems developed to monitor feeding behavior of group-housed Holstein heifers
Assessing sheep behavior through low-power microcontrollers in smart agriculture scenarios
Assessing the accuracy of inline milk fat-to-protein ratio data as an indicator of hyperketonemia in dairy cows in herds with automated milking systems
Assessing the Accuracy of Leg Mounted Sensors for Recording Dairy Cow Behavioural Activity at Pasture, in Cubicle Housing and a Straw Yard
Assessing the Activity of Individual Group-Housed Broilers Throughout Life Using a Passive Radio Frequency Identification System-A Validation Study
Assessing the effect of barns structures and environmental conditions in dairy cattle farms monitored in Northern Italy
Assessing the Homogeneity of Forage Mixtures Using an RGB Camera as Exemplified by Cattle Rations
Assessing the pasturelands and livestock dynamics in Brazil, from 1985 to 2017: A novel approach based on high spatial resolution imagery and Google Earth Engine cloud computing
Assessing the potential of photogrammetry to monitor feed intake of dairy cows
Assessment of a non-invasive acoustic sensor for detecting cattle urination events
Assessment of associations between transition diseases and reproductive performance of dairy cows using survival analysis and decision tree algorithms
Assessment of Cumulative Energy Needs for Chosen Technologies of Cattle Feeding in Barns with Conventional (CFS) and Automated Feeding Systems (AFS)
Assessment of dairy cow heat stress by monitoring drinking behaviour using an embedded imaging system
Assessment of digital technology adoption and access barriers among crop, dairy and livestock producers in Wisconsin
Assessment of feeding, ruminating and locomotion behaviors in dairy cows around calving - a retrospective clinical study to early detect spontaneous disease appearance
Assessment of Laying Hens' Thermal Comfort Using Sound Technology
Assessment of RumiWatch noseband sensors for the quantification of ingestive behaviors of dairy cows at grazing or fed in stalls
Assessment of Sensitivity and Profitability of an Intravaginal Sensor for Remote Calving Prediction in Dairy Cattle

Assessment of the value of information of precision livestock farming: A conceptual framework
Assessment of two wireless reticulo-rumen pH sensors for dairy cows
Assessment of Ventral Tail Base Surface Temperature for the Early Detection of Japanese Black Calves with Fever
Association between body condition score fluctuations and pregnancy loss in Holstein cows
Association between breeding bulls' reactivity to humans or handling and their daily behaviour and growth
Association between Rumination Times Detected by an Ear Tag-Based Accelerometer System and Rumen Physiology in Dairy Cows
Association between Udder and Quarter Level Indicators and Milk Somatic Cell Count in Automatic Milking Systems
Association networks and social temporal dynamics in ewes and lambs
Association of days in close up, gestation length, and rumination around time of calving with disease and pregnancy outcomes in multiparous dairy cows
Association of estrous expression detected by an automated activity monitoring system within 40 days in milk and reproductive performance of lactating Holstein cows
Association of housing and management practices with milk yield, milk composition, and fatty acid profile, predicted using Fourier transform mid-infrared spectroscopy, in farms with automated milking systems
Association of rumination with milk yield of early, mid and late lactation dairy cows
Associations among body energy status, feeding duration and activity with respect to diet energy and protein content in housed dairy cows
Associations between body condition score, locomotion score, and sensor-based time budgets of dairy cattle during the dry period and early lactation
Associations between Feeding Behaviors Collected from an Automated Milk Feeder and Neonatal Calf Diarrhea in Group Housed Dairy Calves: A Case-Control Study
Associations between Gastrointestinal Nematode Infection Burden and Lying Behaviour as Measured by Accelerometers in Periparturient Ewes
Associations between genomic merit for daughter pregnancy rate of Holstein cows and metabolites postpartum and estrus characteristics
Associations between lying behavior and activity and hypocalcemia in grazing dairy cows during the transition period
Associations between peripartum lying and activity behaviour and blood non-esterified fatty acids and b-hydroxybutyrate in grazing dairy cows
Associations between precision sensor data with productivity, health and welfare indicator traits in native black and white dual-purpose cattle under grazing conditions
Associations of Automatically Recorded Body Condition Scores with Measures of Production, Health, and Reproduction
Attribute selection and model evaluation for the maternal and paternal imprinted genes in bovine (Bos Taurus) using supervised machine learning algorithms
Automated aerial animal detection when spatial resolution conditions are varied

Automated Behavior Recognition and Tracking of Group-Housed Pigs with an Improved DeepSORT Method
Automated behavioural monitoring allows assessment of the relationships between cow and calf behaviour and calves' survivability and performance
Automated bioacoustics: methods in ecology and conservation and their potential for animal welfare monitoring
Automated body condition scoring of dairy cows using 3-dimensional feature extraction from multiple body regions
Automated calculation of heart girth measurement in pigs using body surface point clouds
Automated cattle counting using Mask R-CNN in quadcopter vision system
Automated Chicken Counting in Surveillance Camera Environments Based on the Point Supervision Algorithm: LC-DenseFCN
Automated Classification for Visual-Only Postmortem Inspection of Porcine Pathology
Automated Collection and Analysis of Infrared Thermograms for Measuring Eye and Cheek Temperatures in Calves
Automated computer vision system to predict body weight and average daily gain in beef cattle during growing and finishing phases
Automated detection and analysis of piglet suckling behaviour using high-accuracy amodal instance segmentation
Automated detection and analysis of social behaviors among preweaning piglets using key point-based spatial and temporal features
Automated detection and quantification of contact behaviour in pigs using deep learning
Automated Detection of Animals in Low-Resolution Airborne Thermal Imagery
Automated detection of estrous behavior in tie-stall housing using a barometer and accelerometer
Automated detection of health disorders in lactating dairy cattle on pasture: a preliminary study
Automated detection of lameness in sheep using machine learning approaches: novel insights into behavioural differences among lame and non-lame sheep
Automated estrous detection using multiple commercial precision dairy monitoring technologies in synchronized dairy cows
Automated feeding of sheep. 1. Changes in feeding behaviour in response to restricted and ad libitum feeding
Automated feeding of sheep. 2. Feeding behaviour influences the methane emissions of sheep offered restricted diets
Automated Health Monitoring System for Dairy Cows - RumiWatch
Automated identification of chicken distress vocalizations using deep learning models
Automated Individual Pig Localisation, Tracking and Behaviour Metric Extraction Using Deep Learning
Automated measurement of broiler stretching behaviors under four stocking densities via faster region-based convolutional neural network

Automated measurement of dairy cows body size via 3D point cloud data analysis
Automated Measurement of Heart Girth for Pigs Using Two Kinect Depth Sensors
Automated measurement of livestock body based on pose normalisation using statistical shape model
Automated Microclimate Regulation in Agricultural Facilities Using the Air Curtain System
Automated Monitoring of Panting for Feedlot Cattle: Sensor System Accuracy and Individual Variability
Automated monitoring of seasonal and diurnal variation of rumination behaviour: Insights into thermotolerance management of Holstein cows
Automated Muzzle Detection and Biometric Identification via Few-Shot Deep Transfer Learning of Mixed Breed Cattle
Automated pig counting using deep learning
Automated piglet tracking using a single convolutional neural network
Automated prediction of mastitis infection patterns in dairy herds using machine learning
Automated Processing and Phenotype Extraction of Ovine Medical Images Using a Combined Generative Adversarial Network and Computer Vision Pipeline
Automated recognition and discrimination of human-animal interactions using Fisher vector and hidden Markov model
Automated recognition of postures and drinking behaviour for the detection of compromised health in pigs
Automated sheep facial expression classification using deep transfer learning
Automated system for characterizing short-term feeding behavior and real-time forestomach motility in cattle
Automated Tracking Systems for the Assessment of Farmed Poultry
Automated Video Behavior Recognition of Pigs Using Two-Stream Convolutional Networks
Automated video sensing - real world correlations
Automated Virtual Fencing Can Effectively Contain Sheep: Field Trials and Prospects
Automatic activity tracking of goats using drone camera
Automatic Assessment of Keel Bone Damage in Laying Hens at the Slaughter Line
Automatic behavior recognition of group-housed goats using deep learning
Automatic body condition scoring system for dairy cows based on depth-image analysis
Automatic broiler temperature measuring by thermal camera
Automatic counting and positioning of slaughter pigs within the pen using a convolutional neural network and video images
Automatic Detection Method of Dairy Cow Feeding Behaviour Based on YOLO Improved Model and Edge Computing
Automatic detection of feeding- and drinking-related agonistic behavior and dominance in dairy cows
Automatic detection of parturition in pregnant ewes using a three-axis accelerometer
Automatic estimation of dairy cattle body condition score from depth image using

ensemble model
Automatic estimation of dairy cow body condition score based on attention-guided 3D point cloud feature extraction
Automatic identification of individual yaks in in-the-wild images using part-based convolutional networks with self-supervised learning
Automatic Individual Pig Detection and Tracking in Pig Farms
Automatic lameness detection in cattle
Automatic livestock body measurement based on keypoint detection with multiple depth cameras
Automatic Milk Quantity Recording System for Small-Scale Dairy Farms Based on Internet of Things
Automatic monitoring and detection of tail-biting behavior in groups of pigs using video-based deep learning methods
AUTOMATIC MONITORING OF CHICKEN MOVEMENT AND DRINKING TIME USING CONVOLUTIONAL NEURAL NETWORKS
Automatic monitoring of lactation frequency of sows and movement quantification of newborn piglets in farrowing houses using convolutional neural networks
Automatic monitoring system for individual dairy cows based on a deep learning framework that provides identification via body parts and estimation of body condition score
Automatic multi-weigh-station for assessing sheep liveweight in small flocks
Automatic Newcastle disease detection using sound technology and deep learning method
Automatic Position Detection and Posture Recognition of Grouped Pigs Based on Deep Learning
Automatic posture change analysis of lactating sows by action localisation and tube optimisation from untrimmed depth videos
Automatic prediction of stress in piglets (Sus Scrofa) using infrared skin temperature
Automatic recognition method of cow ruminating behaviour based on edge computing
Automatic recognition of dairy cow mastitis from thermal images by a deep learning detector
Automatic recognition of feeding and foraging behaviour in pigs using deep learning
Automatic recognition of lactating sow postures by refined two-stream RGB-D faster R-CNN
Automatic recording of individual oestrus vocalisation in group-housed dairy cattle: development of a cattle call monitor
Automatic scoring of postures in grouped pigs using depth image and CNN-SVM
Automatic Scoring System for Monitoring Foot Pad Dermatitis in Broilers
Automatic segmentation of cattle rib-eye area in ultrasound images using the UNet plus plus deep neural network
Automatic teat detection for rotary milking system based on deep learning algorithms
Automatic tracking of the dairy goat in the surveillance video
Automatic weight measurement of pigs based on 3D images and regression network

Automatic Weight Prediction System for Korean Cattle Using Bayesian Ridge Algorithm on RGB-D Image
Automatically Detected Pecking Activity in Group-Housed Turkeys
Automatically detecting pig position and posture by 2D camera imaging and deep learning
Autonomous System of TMR (Total Mixed Ration) Feed Feeding Robot for Smart Cattle Farm
Autotransporter-based surface expression and complementation of split TreA fragments utilized for the detection of antibodies against bovine leukemia virus
Bag-of-Visual-Words for Cattle Identification from Muzzle Print Images
Barriers to computer vision applications in pig production facilities
Basic motion behavior recognition of single dairy cow based on improved Rexnet 3D network
Bedding system influences lying behaviour in dairy cows
Beef cattle methane emissions measured with tracer-ratio and inverse dispersion modelling techniques
Beef Tenderness Prediction by a Combination of Statistical Methods: Chemometrics and Supervised Learning to Manage Integrative Farm-To-Meat Continuum Data
Behavior assessment and applications for BRD diagnosis: beef
Behavior Classification and Analysis of Grazing Sheep on Pasture with Different Sward Surface Heights Using Machine Learning
Behavior classification of goats using 9-axis mulsensors: The effect of imbalanced datasets on classification performance
Behavior of dairy cows managed outdoors in winter: Effects of weather and paddock soil conditions
Behavior Recognition of Group-ranched Cattle from Video Sequences using Deep Learning
Behavior, health, and productivity of early-lactation dairy cows supplemented with molasses in automated milking systems
Behavioral fractal method associated with GPS tracking to spatial activity sequences of grazing cattle
Behavioral Monitoring Tool for Pig Farmers: Ear Tag Sensors, Machine Intelligence, and Technology Adoption Roadmap
Behaviour classification of extensively grazed sheep using machine learning
Behaviour recognition of pigs and cattle: Journey from computer vision to deep learning
Behavioural Classification of Cattle Using Neck-Mounted Accelerometer-Equipped Collars
Benchmarking of farms with automated milking systems in Canada and associations with milk production and quality
Between- and within-herd variation in blood and milk biomarkers in Holstein cows in early lactation
Beyond pastures, look at plastic: Using Sentinel-2 imagery to map silage bags to improve understanding of cattle intensity

Beyond resistance: Geographies of divergent more-than-human conduct in robotic milking
Big (pig) data and the internet of the swine things: a new paradigm in the industry
Big data in agriculture: Between opportunity and solution
Big Data' in animal health research - opportunities and challenges
Big data-based risk assessment of poultry farms during the 2020/2021 highly pathogenic avian influenza epidemic in Korea
BIGRU-ATTENTION BASED COW BEHAVIOR CLASSIFICATION USING VIDEO DATA FOR PRECISION LIVESTOCK FARMING
Bioimpedance-Measurement-Based Non-Invasive Method for In Ovo Chicken Egg Sexing
Biologically inspired herding of animal groups by robots
Biomarkers from automatic milking system as an indicator of subclinical acidosis and subclinical ketosis in fresh dairy cows
Biomarkers from automatic milking system for cows reproduction success
Biometric facial identification using attention module optimized YOLOv4 for sheep
Biometric identification of sheep via a machine-vision system
Birth conditions affect the longevity of Holstein offspring
Bite-o-Mat: A device to assess the individual manipulative behaviour of group housed pigs
Body Condition Score Change throughout Lactation Utilizing an Automated BCS System: A Descriptive Study
Body Condition Scoring in Dairy Cows - A Conceptual and Systematic Review
Body Dimension Measurements of Qinchuan Cattle with Transfer Learning from LiDAR Sensing
Body size measurement and live body weight estimation for pigs based on back surface point clouds
Bovine Respiratory Disease Diagnosis What Progress Has Been Made in Clinical Diagnosis?
Breathing Pattern Analysis in Cattle Using Infrared Thermography and Computer Vision
Bridging the gap between models and users: A lightweight mobile interface for optimized farming decisions in interactive modeling sessions
Broiler FCR Optimization Using Norm Optimal Terminal Iterative Learning Control
Broiler growth and efficiency in response to relaxed maternal feed restriction
Broiler stunned state detection based on an improved fast region-based convolutional neural network algorithm
Business analysis of IRT, Visual observation, and Ovsynch as breeding strategies in Alberta dairies
C3D-ConvLSTM based cow behaviour classification using video data for precision livestock farming
Calf Birth Weight Predicted Remotely Using Automated in-Paddock Weighing Technology

Calf Posture Recognition Using Convolutional Neural Network
Calving and estrus detection in dairy cattle using a combination of indoor localization and accelerometer sensors
Calving location preference and changes in lying and exploratory behavior of preparturient dairy cattle with access to pasture
Calving time identified by the automatic detection of tail movements and rumination time, and observation of cow behavioural changes
Camshift tracking method based on correlation probability graph for model pig
Can accelerometer ear tags identify behavioural changes in sheep associated with parturition?
Can access to an automated grooming brush and/or a mirror reduce stress of dairy cows kept in social isolation?
Can cattle geolocation data yield behavior-based criteria to inform precision grazing systems on rangeland?
Can machine learning algorithms perform better than multiple linear regression in predicting nitrogen excretion from lactating dairy cows
Can measurements of foraging behaviour predict variation in weight gains of free-ranging cattle?
Can metrics of acceleration provide accurate estimates of energy costs of locomotion on uneven terrain? Using domestic sheep (Ovis aries) as an example
Can Milk Flow Traits Act as Biomarkers of Lameness in Dairy Cows?
Can the use of digital technology improve the cow milk productivity in large dairy herds? Evidence from China's Shandong Province
Can unsupervised learning methods applied to milk recording big data provide new insights into dairy cow health?
Can we detect patterns in behavioral time series of cows using cluster analysis?
Carcass and Primal Composition Predictions Using Camera Vision Systems (CVS) and Dual-Energy X-ray Absorptiometry (DXA) Technologies on Mature Cows
Care in dairy farming with automatic milking systems, identified using an Activity Theory lens
Case Study on Recording Pigs' Daily Activity Patterns with a UHF-RFID System
Case Study: Improving the Quality of Dairy Cow Reconstruction with a Deep Learning-Based Framework
Catering for unique tastes: Targeting grey-sheep users recommender systems through one-class machine learning
Cattle behavior recognition based on feature fusion under a dual attention mechanism
Cattle body detection based on YOLOv5-ASFF for precision livestock farming
Cattle counting in the wild with geolocated aerial images in large pasture areas
Cattle Detection Using Oblique UAV Images
Cattle identification system: a comparative analysis of SIFT, SURF and ORB feature descriptors
Cattle identification with muzzle pattern using computer vision technology: a critical review and prospective

Cattle Recognition: A New Frontier in Visual Animal Biometrics Research
Cattle segmentation and contour extraction based on Mask R-CNN for precision livestock farming
Cattle weight estimation using active contour models and regression trees Bagging
CattleFaceNet: A cattle face identification approach based on RetinaFace and ArcFace loss
Center clustering network improves piglet counting under occlusion
Certain economic aspects of the installation of a sensor-based automatic animal monitoring system in an intensive dairy herd
Challenges and Tendencies of Automatic Milking Systems (AMS): A 20-Years Systematic Review of Literature and Patents
Challenges to Use Machine Learning in Agricultural Big Data: A Systematic Literature Review
Change in Rumination Behavior Parameters around Calving in Cows with Subclinical Ketosis Diagnosed during 30 Days after Calving
Change of Ruminoreticular Temperature and Body Activity before and after Parturition in Hanwoo (Bos taurus coreanae) Cows
Changes in activity and object manipulation before tail damage in finisher pigs as an early detector of tail biting
Changes in electrical conductivity, milk production rate and milk flow rate prior to clinical mastitis confirmation
Changes in the behaviour before normal calving to predict its onset in Mediterranean buffaloes heifers
Changes in the suckling behaviour of beef calves at 1 month and 4 months of age and effect on cow production variables
Changes to steps, lying, and eating behavior during lactation in Jersey and Holstein cows and the relationship to feed intake, yield, and weight
Characterising Free-Range Layer Flocks Using Unsupervised Cluster Analysis
Characteristics of thermal images of the mammary gland and of performance in sows differing in health status and parity
Characterization of swine behavior and production using measurements collected via indoor positioning system
Chick Embryo Growth Modeling Using Near-Infrared Sensor and Non-Linear Least Square Fitting of Egg Opacity Values
Chicken Image Segmentation via Multi-Scale Attention-Based Deep Convolutional Neural Network
ChickenNet-an end-to-end approach for plumage condition assessment of laying hens in commercial farms using computer vision
ChickTrack-A quantitative tracking tool for measuring chicken activity
Classification and Analysis of Multiple Cattle Unitary Behaviors and Movements Based on Machine Learning Methods
Classification and Feature Extraction Using Supervised and Unsupervised Machine Learning Approach for Broiler Woody Breast Myopathy Detection

Classification of broiler behaviours using triaxial accelerometer and machine learning
Classification of Cattle Behaviours Using Neck-Mounted Accelerometer-Equipped Collars and Convolutional Neural Networks
Classification of dairy cow excretory events using a tail-mounted accelerometer
Classification of drinking and drinker-playing in pigs by a video-based deep learning method
Classification of environmental factors potentially motivating for dairy cows to access shade
Classification of ingestive-related cow behaviours using RumiWatch halter and neck-mounted accelerometers
Classification of multiple cattle behavior patterns using a recurrent neural network with long short-term memory and inertial measurement units
Classification Performance of Machine Learning Methods for Identifying Resistance, Resilience, and Susceptibility to Haemonchus contortus Infections in Sheep
Classifying animal behavior from accelerometry data via recurrent neural networks
Classifying Ingestive Behavior of Dairy Cows via Automatic Sound Recognition
Classifying season long livestock grazing behavior with the use of a low-cost GPS and accelerometer
Classifying the posture and activity of ewes and lambs using accelerometers and machine learning on a commercial flock
Claw block application improves locomotion and weight-bearing characteristics in cattle with foot diseases
Cleaning procedures and cleanliness assessments of bucket milkers and suckling buckets on Japanese dairy farms
Cleaning quality and animal behaviour with the use of a dung-removal robot
Cleansing data from an electronic feeding station to improve estimation of feed efficiency
Climate Change Mitigation Tool Implemented through an Integrated and Resilient System to Measure and Monitor Operating Variables, Applied to Natural Wastewater Treatment Systems (NTSW) in Livestock Farms
Clinical examination of cattle. Part 2: calves, technology and ancillary testing
Closed circuit xenon delivery for 72h in neonatal piglets following hypoxic insult using an ambient pressure automated control system: Development, technical evaluation and pulmonary effects
Cloud services integration for farm animals' behavior studies based on smartphones as activity sensors
Cloud/edge computing for compliance in the Brazilian livestock supply chain
Cluster index for estimating thermal poultry stress (gallus gallus domesticus)
Color Machine Vision Design Methodology of a Part-Presentation Algorithm for Automated Poultry Handling
Combination of Sensor Data and Health Monitoring for Early Detection of Subclinical Ketosis in Dairy Cows
Combined spectral and speech features for pig speech recognition

Combining computer vision score and conventional meat quality traits to estimate the intramuscular fat content using machine learning in pigs
Combining expert knowledge and machine-learning to classify herd types in livestock systems
Combining multivariate cumulative sum control charts with principal component analysis and partial least squares model to detect sickness behaviour in dairy cattle
Comfort and health evaluation of live mutton sheep during the transportation based on wearable multi-sensor system
Comparative Analysis of Data-Driven Techniques to Predict Heating and Cooling Energy Requirements of Poultry Buildings
Comparative analysis of machine learning algorithms for predicting live weight of Hereford cows
COMPARATIVE EVALUATION OF POULTRY-HUMAN AND POULTRY-ROBOT AVOIDANCE DISTANCES
Comparative Evaluation of the Dynamics of Animal Husbandry Air Pollutant Emissions Using an IoT Platform for Farms
Comparative study on poultry target tracking algorithms based on a deep regression network
Comparing gradual debonding strategies after prolonged cow-calf contact: Stress responses, performance, and health of dairy cow and calf
Comparing regression, naive Bayes, and random forest methods in the prediction of individual survival to second lactation in Holstein cattle
Comparing State-of-the-Art Deep Learning Algorithms for the Automated Detection and Tracking of Black Cattle
Comparing steam-flaked and pelleted barley grain in a feed-first guided-flow automated milking system for Holstein cows
Comparing the Predictive Ability of Machine Learning Methods in Predicting the Live Body Weight of Beetal Goats of Pakistan
Comparison of behavioral patterns of dairy cows with natural estrus and induced ovulation detected by an ear-tag based accelerometer
Comparison of data analytics strategies in computer vision systems to predict pig body composition traits from 3D images
Comparison of forecast models of production of dairy cows combining animal and diet parameters
Comparison of machine learning methods to predict udder health status based on somatic cell counts in dairy cows
Comparison of machine learning models for bluetongue risk prediction: a seroprevalence study on small ruminants
Comparison of methods for predicting cow composite somatic cell counts
Comparison of methods to predict feed intake and residual feed intake using behavioral and metabolite data in addition to classical performance variables
Comparison of neuro-fuzzy and neural networks techniques for estimating ammonia concentration in poultry farms

Comparison of Sensory Qualities in Eggs from Three Breeds Based on Electronic Sensory Evaluations
Comparison of the automated monitoring of the sow activity in farrowing pens using video and accelerometer data
Comparison of the conventional and robotic milking system for the parameters of composition and quality of milk, in grazing system
Comparison of the feeding behaviour of primiparous and multiparous Jersey and Holstein cows kept under equal conditions throughout lactation
Comparison of virtually fencing and electrically fencing sheep for pasture management
Competition Strategies of Metritic and Healthy Transition Cows
Comprehensive analysis of machine learning models for prediction of sub-clinical mastitis: Deep Learning and Gradient-Boosted Trees outperform other models
Computer vision and weigh scale-based prediction of milk yield and udder traits for individual cows
COMPUTER VISION FOR MORPHOMETRIC EVALUATION OF BROILER CHICKEN BONES
Computer vision system and near-infrared spectroscopy for identification and classification of chicken with wooden breast, and physicochemical and technological characterization
Computer vision system for measuring individual cow feed intake using RGB-D camera and deep learning algorithms
Computer vision system for superpixel classification and segmentation of sheep
Computer Vision-Based Approach for Automatic Detection of Dairy Cow Breed
Computer vision-based weight estimation of livestock: a systematic literature review
Computer-Assisted Automatic Egg Fertility Control
Computer-Vision-Based Indexes for Analyzing Broiler Response to Rearing Environment: A Proof of Concept
Connected cows and cyber chickens? Stocktaking and case studies of digital livestock tools in Kenya and India
Connecting farmer mental health with cow health and welfare on dairy farms using robotic milking systems
Consumer Perceptions of Precision Livestock Farming-A Qualitative Study in Three European Countries
Continuous Monitoring of Pigs in Fattening Using a Multi-Sensor System: Behavior Patterns
Contour Extraction of Individual Cattle From an Image Using Enhanced Mask R-CNN Instance Segmentation Method
Controlled Intestinal Microbiota Colonisation in Broilers under the Industrial Production System
Core and peripheral site measurement of body temperature in short wool sheep
CORF3D contour maps with application to Holstein cattle recognition from RGB and thermal images
Correlations among Ultrasonographic, Physicochemical and Sensory Characteristics of

Pectoralis Major Muscles in Turkeys Reared in a Sustainable Farming System
Correlations, variations, and modelling of indoor environment in a mechanically-ventilated pig building
Counting Cattle in UAV Images-Dealing with Clustered Animals and Animal/Background Contrast Changes
Cow- and herd-level risk factors for lameness in partly housed pasture-based dairy cows
Cow identification based on fusion of deep parts features
Cow identification in free-stall barns based on an improved Mask R-CNN and an SVM
Cow individual activity response to the accumulation of heat load duration
Cows' legs tracking and lameness detection in dairy cattle using video analysis and Siamese neural networks
Cows with paratuberculosis (Johne's disease) alter their lying behavior around peak lactation
Crowd monitoring in dairy cattle-real-time VOC profiling by direct mass spectrometry
CRUMBLER: A tool for the prediction of ancestry in cattle
Curve Skeleton Extraction from Incomplete Point Clouds of Livestock and Its Application in Posture Evaluation
Cutting of sheep carcass using 3D point cloud with dual-robot system
CYBELE - Fostering precision agriculture & livestock farming through secure access to large-scale HPC enabled virtual industrial experimentation environments fostering scalable big data analytics
Cytometric fingerprinting and machine learning (CFML): A novel label-free, objective method for routine mastitis screening
Daily behavioral measures recorded by precision technology devices may indicate bovine respiratory disease status in preweaned dairy calves
Daily rumination time of lactating dairy cows under heat stress conditions
Dairy 4.0: Intelligent Communication Ecosystem for the Cattle Animal Welfare with Blockchain and IoT Enabled Technologies
Dairy cattle behavior classifications based on decision tree learning using 3-axis neck-mounted accelerometers
Dairy Cattle Rumen Bolus Developments with Special Regard to the Applicable Artificial Intelligence (AI) Methods
Dairy Cow Behavior Is Affected by Period, Time of Day and Housing
Dairy cow lameness detection using a back curvature feature
Dairy Cows Activity under Heat Stress: A Case Study in Spain
Dairy cows' contact networks derived from videos of eight cameras
Dairy cows' localisation and feeding behaviour monitoring using a combination of IMU and RFID network
Dairy Cows' Temperament and Milking Performance during the Adaptation to an Automatic Milking System
DairyCare 'blueprint for action': husbandry for wellbeing
Data considerations for developing deep learning models for dairy applications: A simulation study on mastitis detection

Data synchronization for gas emission measurements from dairy cattle: A matched filter approach
Data-driven decision making in pig farming: A review of the literature
Data-driven decision support in livestock farming for improved animal health, welfare and greenhouse gas emissions: Overview and challenges
Decentralised and Predictive System for Efficient Agri-Transactions Through Blockchain Technology
Decision support system to classify the vulnerability of broiler production system to heat stress based on fuzzy logic
Decision tree machine learning applied to bovine tuberculosis risk factors to aid disease control decision making
Deep cascaded convolutional models for cattle pose estimation
Deep Learning and Machine Vision Approaches for Posture Detection of Individual Pigs
Deep Learning Based Real-Time Body Condition Score Classification System
Deep Learning for Laying Hen Activity Recognition Using Wearable Sensors
Deep learning for population size history inference: Design, comparison and combination with approximate Bayesian computation
Deep learning image recognition of cow behavior and an open data set acquired near an automatic milking robot
Deep learning to map concentrated animal feeding operations
Deep learning-based automatic dairy cow ocular surface temperature detection from thermal images
Deep learning-based cattle behaviour classification using joint time-frequency data representation
Deep Learning-Based Cattle Vocal Classification Model and Real-Time Livestock Monitoring System with Noise Filtering
Deep Learning-Based Cow Tail Detection and Tracking for Precision Livestock Farming
Deep learning-based hierarchical cattle behavior recognition with spatio- temporal information
Deep learning-based model classifies thermal conditions in dairy cows using infrared thermography
Deep transfer learning in sheep activity recognition using accelerometer data
Deep-Learning-Based Automatic Monitoring of Pigs' Physico-Temporal Activities at Different Greenhouse Gas Concentrations
Defect detection in eggshell using a vision system to ensure the incubation in poultry production
Deployment of Wireless Sensor Network and IoT Platform to Implement an Intelligent Animal Monitoring System
Depth Density Achieves a Better Result for Semantic Segmentation with the Kinect System
Description of Behavioral Patterns Displayed by a Recently Weaned Cohort of Healthy Dairy Calves
DESIGN AND DEVELOPMENT OF A BROILER MORTALITY REMOVAL ROBOT

DESIGN AND FIELD TEST OF A LOW-COST DEVICE FOR REAL-TIME LIVESTOCK TRACKING USING GPS/LORA COMMUNICATION
Design and Implementation of a Malfunction Detection System for Livestock Ventilation Devices in Smart Poultry Farms
Design and Implementation of Poultry Farming Information Management System Based on Cloud Database
Design mechanism and performance evaluation of photonic crystal fiber (PCF)-based sensor in the THz regime for sensing noxious chemical substrates of poultry feed
Design of a Highly Sensitive Self-Reference Tamm-Plasmon-Polariton Sensor Employing Ti(3)C(2)Tx MXene
Design of Scalable IoT Architecture Based on AWS for Smart Livestock
Designing and Simulating the Control Process of a Feed Pusher Robot Used on a Dairy Farm
Designing Automated Milking Dairy Facilities to Maximize Labor Efficiency
Detecting Animal Contacts-A Deep Learning-Based Pig Detection and Tracking Approach for the Quantification of Social Contacts
Detecting Dairy Cow Behavior Using Vision Technology
Detecting Heat Stress in Dairy Cattle Using Neck-Mounted Activity Collars
Detecting sheep in UAV images
Detecting the Bitterness of Milk-Protein-Derived Peptides Using an Electronic Tongue
Detection and Analysis of Sow Targets Based on Image Vision
Detection and tracking of chickens in low-light images using YOLO network and Kalman filter
Detection and tracking of pigs in natural environments based on video analysis
Detection of aggressive behaviours in pigs using a RealSence depth sensor
Detection of changes in the circadian rhythm of cattle in relation to disease, stress, and reproductive events
Detection of cow mounting behavior using region geometry and optical flow characteristics
Detection of genomic regions associated malformations in newborn piglets: a machine-learning approach
Detection of NH3 in poultry housing based on tunable diode laser absorption spectroscopy combined with a micro circular absorption cell
Detection of porcine epidemic diarrhea virus-neutralizing antibody using high-throughput imaging cytometry
Detection of rumination in cattle using an accelerometer ear-tag: A comparison of analytical methods and individual animal and generic models
Detection of sick broilers by digital image processing and deep learning
Detection of Steer Defecation Events using an Accelerometer
Detection of the respiratory rate of standing cows by combining the Deeplab V3+semantic segmentation model with the phase-based video magnification algorithm
Detection of Volatile Organic Compounds (VOCs) in Livestock Houses Based on Electronic Nose

Detection system of dead and sick chickens in large scale farms based on artificial intelligence
Detection, identification and posture recognition of cattle with satellites, aerial photography and UAVs using deep learning techniques
Determination of Body Parts in Holstein Friesian Cows Comparing Neural Networks and k Nearest Neighbour Classification
Determination of ewe behaviour around lambing time and prediction of parturition 7 days prior to lambing by tri-axial accelerometer sensors in an extensive farming system
Determination of Non-Digestible Parts in Dairy Cattle Feces Using U-NET and F-CRN Architectures
Determine the vertical ground reaction forces and knee mechanics with different gait inclinations in the sheep model
Determining pig holding type from British movement data using analytical and machine learning approaches
Developing a new thermal comfort prediction model and web-based application for heat stress assessment in dairy cows
Developing a Simulated Online Model That Integrates GNSS, Accelerometer and Weather Data to Detect Parturition Events in Grazing Sheep: A Machine Learning Approach
Developing and Evaluating Poultry Preening Behavior Detectors via Mask Region-Based Convolutional Neural Network
Developing and evaluating threshold-based algorithms to detect drinking behavior in dairy cows using reticulorumen temperature
Development and application of an autonomous data logger to measure environmental variables in livestock farming
Development and application of an image acquisition system for characterizing sow behaviors in farrowing stalls
Development and evaluation of a method to detect broilers continuously walking around feeder as an indication of restricted feeding behaviors
DEVELOPMENT AND OPTIMIZATION OF A DEEP-LEARNING-BASED EGG-COLLECTING ROBOT
Development and performance of an automated fecal egg count system for small ruminant strongylids
Development and validation of a neural network for the automated detection of horn flies on cattle
Development and validation of a predictive model for calving time based on sensor measurements of ingestive behavior in dairy cows
DEVELOPMENT AND VALIDATION OF AN ANIMAL THERMAL ENVIRONMENT INTERACTION LABORATORY
DEVELOPMENT AND VALIDATION OF AN AUTONOMOUS RADIO-FREQUENCY IDENTIFICATION CONTROLLED SOAKING SYSTEM FOR DAIRY CATTLE
Development and Validation of an Energy Consumption Model for Animal Houses Achieving Precision Livestock Farming
Development of a computer vision system to detect inactivity in group-housed pigs

Development of a methodological framework for a robust prediction of the main behaviours of dairy cows using a combination of machine learning algorithms on accelerometer data
Development of a multi-use decision support system for scientific management and breeding of sheep
Development of a New Wearable 3D Sensor Node and Innovative Open Classification System for Dairy Cows' Behavior
Development of a recurrent neural networks-based calving prediction model using activity and behavioral data
Development of a subacute ruminal acidosis risk score and its prediction using milk mid-infrared spectra in early-lactation cows
DEVELOPMENT OF A WIRELESS MEASUREMENT SYSTEM FOR CLASSIFYING COW BEHAVIOR USING ACCELEROMETER DATA AND LOCATION DATA
Development of activity collecting system for grazing cattle in vast land
Development of an Anomaly Detection System for Cattle Using Infrared Image and Machine Learning
Development of an Artificial Neural Network for the Detection of Supporting Hindlimb Lameness: A Pilot Study in Working Dogs
Development of an Automated Body Temperature Detection Platform for Face Recognition in Cattle with YOLO V3-Tiny Deep Learning and Infrared Thermal Imaging
Development of an Automated Pain Facial Expression Detection System for Sheep (Ovis Aries)
Development of an Early Embryo Detection Methodology for Quail Eggs Using a Thermal Micro Camera and the YOLO Deep Learning Algorithm
Development of Noncontact Body Temperature Monitoring and Prediction System for Livestock Cattle
DEVELOPMENT OF SOFTWARE FOR ANALYSIS OF BEHAVIOR AND WELFARE OF BROILERS
Development of sound-based poultry health monitoring tool for automated sneeze detection
Development of Tetrapod Zinc Oxide-Based UV Sensor for Precision Livestock Farming and Productivity
Development status and trend of agricultural robot technology
Development, Validation and Preliminary Experiments of a Measuring Technique for Eggs Aging Estimation Based on Pulse Phase Thermography
Diagnosing sub-acute rumen acidosis in cows in the post-calving period with digital technologies
Diagnosis of dairy cow diseases by knowledge-driven deep learning based on the text reports of illness state
Dietary Phosphorus and Calcium Utilization in Growing Pigs: Requirements and Improvements
Differentiation of Livestock Internal Organs Using Visible and Short-Wave Infrared Hyperspectral Imaging Sensors

Diffusion of precision livestock farming technologies in dairy cattle farms
Digestibility contributes to between-animal variation in feed efficiency in beef cows
DigiPig: First Developments of an Automated Monitoring System for Body, Head and Tail Detection in Intensive Pig Farming
Digital imaging assisted geometry of chicken eggs using Hugelschaffer's model
Digital technology adoption in livestock production with a special focus on ruminant farming
Digital tools for assessing animal behaviour and facilitating rangeland grazing operations
Digital Transformation for Sustainable Future - Agriculture 4.0: A review
Digital Twins in Livestock Farming
Digital twins in smart farming
Digitization of the barn - current status and perspectives
Dimension-reduced spatiotemporal network for lameness detection in dairy cows
Direct Measurements of the Volume Flow Rate and Emissions in a Large Naturally Ventilated Building
Discovery of different metabotypes in overconditioned dairy cows by means of machine learning
Discovery of significant porcine SNPs for swine breed identification by a hybrid of information gain, genetic algorithm, and frequency feature selection technique
Discriminating pathological, reproductive or stress conditions in cows using machine learning on sensor-based activity data
Discrimination of biting and chewing behaviour in sheep using a tri-axial accelerometer
Discriminative power of acoustic features for jaw movement classification in cattle and sheep
Disentangling data dependency using cross-validation strategies to evaluate prediction quality of cattle grazing activities using machine learning algorithms and wearable sensor data
Diurnal rhythms of group-housed layer pullets with free choices between light and dim environments
Division of Pig Growth Stages According to Body Component Variation using Computer Vision
DNAzyme-Amplified Electrochemical Biosensor Coupled with pH Meter for Ca2+ Determination at Variable pH Environments
Do Walking Distance and Time Away from the Paddock Influence Daily Behaviour Patterns and Milk Yield of Grazing Dairy Cows?
Do we automatically detect health- or general welfare-related issues? A framework
Does Virtual Fencing Work for Grazing Dairy Cattle?
Doing nothing and what it looks like: inactivity in fattening cattle
Domestic hens succeed at serial reversal learning and perceptual concept generalisation using a new automated touchscreen device
Domestic pig sound classification based on TransformerCNN
Drone approach parameters leading to lower stress sheep flocking and movement: sky

shepherding
Dual attention-guided feature pyramid network for instance segmentation of group pigs
Dynamic analysis and verification of the motion of poultry eggs on supporting rollers
Dynamic Changes in Progesterone Concentration in Cows' Milk Determined by the At-Line Milk Analysis System Herd Navigator(TM)
Dynamic cooling strategy based on individual animal response mitigated heat stress in dairy cows
DYNAMIC HEAT FLUX MEASUREMENTS FROM FINISHING PIGS
Dynamics of automatically generated body condition scores during early lactation and pregnancy at first artificial insemination of Holstein cows
Dynamics of Sows' Activity Housed in Farrowing Pens with Possibility of Temporary Crating might Indicate the Time When Sows Should be Confined in a Crate before the Onset of Farrowing
Dynamics of Thermolysis and Skin Microstructure in Water Buffaloes Reared in Humid Tropical Climate-A Microscopic and Thermographic Study
Early Detection of Respiratory Diseases in Calves by Use of an Ear-Attached Accelerometer
Early life indicators of first lactation milk yield and the effect of treatment for bovine respiratory disease on survivability and risk of pregnancy in Holstein dairy cattle
Early prediction of respiratory disease in preweaning dairy calves using feeding and activity behaviors
Economic Advantages of Individual Animal Identification in Fattening Pigs
Economic Viability of Adoption of Automated Oestrus Detection Technologies on Dairy Farms: A Review
Economics of timed artificial insemination with unsorted or sexed semen in a high-producing, pasture-based dairy production system
Effect of a mechanical grooming brush on the behavior and health of recently weaned heifer calves
Effect of a targeted reproductive management program designed to prioritize insemination at detected estrus and optimize time to insemination on the reproductive performance of lactating dairy cows
Effect of an automated marking system on aggressive behavior of precision-fed broiler breeder chicks
Effect of dry-off management on milking behavior, milk yield, and somatic cell count of dairy cows milked in automated milking systems
Effect of gonadotropin-releasing hormone administered at the time of artificial insemination for cows detected in estrus by conventional estrus detection or an automated activity-monitoring system
Effect of lameness on feeding behavior of zero grazed Jersey dairy cows
Effect of low frequency oscillations during milking on udder temperature and welfare of dairy cows
Effect of Minimum Milking Interval on Traffic and Milk Production of Cows Milked by

a Pasture Based Automatic Milking System
Effect of oral calcium administration on metabolic status and uterine health of dairy cows with reduced postpartum rumination and eating time
Effect of prepartum exercise on lying behavior, labor length, and cortisol concentrations
Effect of the amount of concentrate offered in an automated milking system on dry matter intake, milk yield, milk composition, ruminal digestion, and behavior of primiparous Holstein cows fed isocaloric diets
Effect of transition to an automated milking system for a tie-stall barn on milk production and cow condition
EFFECT OF TWO COOLING FREQUENCIES ON RESPIRATION RATE IN LACTATING DAIRY COWS UNDER HOT AND HUMID CLIMATE CONDITIONS
EFFECT OF WAVELET DE-NOISING ON THE CLASSIFICATION OF PIG BEHAVIOUR
Effect of weather on activity and lying behaviour in clinically healthy grazing dairy cows during the transition period
Effects of adding an automated monitoring device to the health screening of postpartum Holstein cows on survival and productive and reproductive performances
Effects of Body-Mounted Inertial Measurement Unit (IMU) Backpacks on Space Use and Behaviors of Laying Hens in a Perchery System
Effects of Climatic Conditions on the Lying Behavior of a Group of Primiparous Dairy Cows
Effects of concentrate allowance and individual dairy cow personality traits on behavior and production of dairy cows milked in a free-traffic automated milking system
Effects of concentrate location on the behavior and production of dairy cows milked in a free-traffic automated milking system
Effects of drop height, conveyor belt speed, and acceleration on the welfare of broiler chickens in early and later life
Effects of feeding level, milking frequency, and single injection of cabergoline on feed intake, milk yield, milk leakage, and clinical udder characteristics during dry-off in dairy cows
Effects of flooring surface and a supplemental heat source on location preference, behaviour and growth rates of dairy goat kids
Effects of free-choice pasture access on lameness recovery and behavior of lame dairy cattle
Effects of GnRH and hCG administration during early luteal phase on estrous cycle length, expression of estrus and fertility in lactating dairy cows
Effects of ground robot manipulation on hen floor egg reduction, production performance, stress response, bone quality, and behavior
Effects of heat stress on rumination activity in Holstein-Gyr dry cows
Effects of Heated Drinking Water on the Growth Performance and Rumen Functionality of Fattening Charolaise Beef Cattle in Winter
Effects of herbage mass and herbage allowance on bite mass of grazing dairy cows
Effects of liveweight and incisor arcade breadth on bite mass of grazing Holstein-

Friesian dairy cows
Effects of Maternal Nutrient Restriction and Melatonin Supplementation on Cardiomyocyte Cell Development Parameters Using Machine Learning Techniques
Effects of milking machine settings and teat liners on bovine udder health
Effects of nematode parasitism on activity patterns in first-season grazing cattle
Effects of rumen-protected methionine on lactation performance and physiological variables during a heat stress challenge in lactating Holstein cows
Effects of the environment and animal behavior on nutrient requirements for gestating sows: Future improvements in precision feeding
Effects of winter housing system on hygiene, udder health, frostbite, and rumination of dairy cows
Efficacy of statistical process control procedures to identify deviations in continuously measured physiologic and behavioral variables in beef steers experimentally challenged with Mannheimia haemolytica
Efficacy of statistical process control procedures to monitor deviations in physical behavior for preclinical detection of bovine respiratory disease in feedlot cattle
Efficient Herd - Outlier Detection in Livestock Monitoring System Based on Density - Based Spatial Clustering
Eggshell deformation detection applying computer vision
Eggshell-inspired membrane-shell strategy for simultaneously improving the sensitivity and detection range of strain sensors
Electromagnetic Pigging System Based on Sandwich Differential Planar Coil
EmbeddedPigCount: Pig Counting with Video Object Detection and Tracking on an Embedded Board
EmbeddedPigDet-Fast and Accurate Pig Detection for Embedded Board Implementations
Emerging Precision Management Methods in Poultry Sector
Energy partitioning by broiler breeder hens in conventional daily-restricted feeding and precision feeding systems
Energy-aware cluster-based routing optimization for WSNs in the livestock industry
Enhanced LiteHRNet based sheep weight estimation using RGB-D images
Enhanced Mask R-CNN for herd segmentation
Enhancement of instrumented ultrasonic tracking images using deep learning
Enhancing Animal Disease Resistance, Production Efficiency, and Welfare through Precise Genome Editing
Enhancing the value of meat inspection records for broiler health and welfare surveillance: longitudinal detection of relational patterns
EnsemblePigDet: Ensemble Deep Learning for Accurate Pig Detection
Environment-, health-, performance- and welfare-related parameters in pig barns with natural and mechanical ventilation
Environmental Impacts of Pig and Poultry Production: Insights From a Systematic Review
Environmental monitoring in a poultry farm using an instrument developed with the

internet of things concept
Environmental Perception Q-Learning to Prolong the Lifetime of Poultry Farm Monitoring Networks
Environmental Risk Factors Influence the Frequency of Coughing and Sneezing Episodes in Finisher Pigs on a Farm Free of Respiratory Disease
Establishing a Smart Farm-Scale Piggery Wastewater Treatment System with the Internet of Things (IoT) Applications
ESTABLISHING MODULARIZED ENVIRONMENTAL MEASUREMENTS AND ANALYZING AND APPLYING ENVIRONMENTAL INFORMATION FOR A WINDOWLESS BROILER HOUSE IN TAIWAN
Establishment of a feed intake prediction model based on eating time, ruminating time and dietary composition
Estimates of genetic parameters for feeding behavior traits and their associations with feed efficiency in Holstein cows
Estimates of intra- and interclass correlation coefficients for rump touches and the number of steps during estrus in postpartum cows
Estimating Amino Acid Requirements in Real-Time for Precision-Fed Pigs: The Challenge of Variability among Individuals
Estimating and tuning adaptive action plans for the control of smart interconnected poultry condominiums
Estimating Body Condition Score in Dairy Cows From Depth Images Using Convolutional Neural Networks, Transfer Learning and Model Ensembling Techniques
Estimating body weight of pigs from posture analysis using a depth camera
Estimating body weight, body condition score, and type traits in dairy cows using three dimensional cameras and manual body measurements
Estimating Conformational Traits in Dairy Cattle With DeepAPS: A Two-Step Deep Learning Automated Phenotyping and Segmentation Approach
Estimating genetics of body dimensions and activity levels in pigs using automated pose estimation
Estimating milk yield for individual cows using measurements of total milk flow
Estimating pasture aboveground biomass under an integrated crop-livestock system based on spectral and texture measures derived from UAV images
Estimating risk probabilities for sickness from behavioural patterns to identify health challenges in dairy cows with multivariate cumulative sum control charts
Estimating the nonlinear association of online somatic cell count, lactate dehydrogenase, and electrical conductivity with milk yield
Estimation of beef cow body condition score: a machine learning approach using three-dimensional image data and a simple approach with heart girth measurements
Estimation of Genetic Parameters for Heifer and Cow Fertility Traits Derived from On-Farm AI Service Records of South African Holstein Cattle
Estimation of Grazing Activity of Dairy Goats Using Accelerometers and Global Positioning System
Estimation of Muscle Scores of Live Pigs Using a Kinect Camera

Estimation of particulate matter and gaseous concentrations using low-cost sensors from broiler houses
Estimation of Spatial-Temporal Distribution of Grazing Intensity Based on Sheep Trajectory Data
Estimation of the Gender Ratio of Chickens Based on Computer Vision: Dataset and Exploration
Estimation of the genetic milk yield parameters of Holstein cattle under heat stress in South Korea
Estrous characteristics and reproductive outcomes of Holstein heifers treated with 2 prostaglandin formulations and detected in estrus by an automated estrous detection or mounting device
Estrous detection by continuous measurements of vaginal temperature and conductivity with supervised machine learning in cattle
Estrous detection with an activity and rumination monitoring system in an organic grazing and a low-input conventional dairy herd
Estrus Detection in a Dairy Herd Using an Electronic Nose by Direct Sampling on the Perineal Region
Estrus detection in tie-stall housed cows through supervised machine learning using a multimodal tail-attached device
Estrus Prediction Models for Dairy Gyr Heifers
Evaluating Alternatives to Locomotion Scoring for Detecting Lameness in Pasture-Based Dairy Cattle in New Zealand: In-Parlour Scoring
Evaluating Alternatives to Locomotion Scoring for Lameness Detection in Pasture-Based Dairy Cows in New Zealand: Infra-Red Thermography
Evaluating automated infrared thermography and vulva exposure tracking as components of an estrus detection platform in a commercial dairy herd
Evaluating avoidance distance and fleeing speed of broilers exposed to aerial systems
Evaluating Convolutional Neural Networks for Cage-Free Floor Egg Detection
Evaluating the performance of machine learning methods and variable selection methods for predicting difficult-to-measure traits in Holstein dairy cattle using milk infrared spectral data
EVALUATING TWO LOW-COST GPS RECEIVERS FOR ACCURACY AND EVENTUAL USE IN PASTURED CATTLE RESEARCH
Evaluation and application potential of an accelerometer-based collar device for measuring grazing behavior of dairy cows
Evaluation and characterization of estrus alerts and behavioral parameters generated by an ear-attached accelerometer-based system for automated detection of estrus
Evaluation and prediction of salt effects on pig muscle by deep UV and machine learning
Evaluation of a Binary Classification Approach to Detect Herbage Scarcity Based on Behavioral Responses of Grazing Dairy Cows
Evaluation of a commercial intravaginal thermometer to predict calving in a Hungarian Holstein-Friesian dairy farm

Evaluation of a Novel Infrared Thermography Projection to Assess Udder Health in Primigravid Dairy Heifers
Evaluation of active dried yeast in the diets of feedlot steers. II. Effects on rumen pH and liver health of feedlot steers
Evaluation of activity meters for estrus detection: A stochastic bioeconomic modeling approach
Evaluation of an Accelerometer-Based Device for Testing the Softness of Bedding Materials Used for Livestock
Evaluation of an Active LF Tracking System and Data Processing Methods for Livestock Precision Farming in the Poultry Sector
Evaluation of an ear-attached accelerometer for detecting estrus events in indoor housed dairy cows
Evaluation of an indwelling bolus equipped with a triaxial accelerometer for the characterisation of the diurnal pattern of bovine reticuloruminal contractions
Evaluation of beef cattle temperament by eye temperature using infrared thermography technology
EVALUATION OF BEEF CATTLE TEMPERAMENT USING VIDEO TECHNOLOGY
Evaluation of biomarkers of heat stress using automatic health monitoring system in dairy cows
Evaluation of cartilage biomechanics and knee joint microenvironment after different cell-based treatments in a sheep model of early osteoarthritis
Evaluation of Deep Learning for Automatic Multi-View Face Detection in Cattle
Evaluation of environmental and comfort improvements on affective welfare in heifer calves on smallholder dairy farms
Evaluation of environmental and physiological indicators in lactating dairy cows exposed to heat stress
Evaluation of Goat Leather Quality Based on Computational Vision Techniques
Evaluation of Heat Stress Effects in Different Geographical Areas on Milk and Rumen Characteristics in Holstein Dairy Cows Using Robot Milking and Rumen Sensors: A Survey in South Korea
Evaluation of lameness detection using radar sensing in ruminants
Evaluation of pig behavior changes related to temperature, relative humidity, volatile organic compounds, and illuminance
Evaluation of Precision Livestock Technology and Human Scoring of Nursery Pigs in a Controlled Immune Challenge Experiment
Evaluation of pre-processing methods for the prediction of cattle behaviour from accelerometer data
Evaluation of proper sensor position for classification of sheep behaviour through accelerometers
Evaluation of proximal sensing technologies for mapping bovine urine patches under grazing pastures
Evaluation of remote monitoring units for estimating body weight and supplement intake of grazing cattle

Evaluation of ruminal motility in cattle by a bolus-type wireless sensor
Evaluation of ruminal motility using an indwelling 3-axis accelerometer in the reticulum in cattle
Evaluation of six machine learning classification algorithms in pig breed identification using SNPs array data
Evaluation of solar photovoltaic systems to shade cows in a pasture-based dairy herd
Evaluation of test duration for feed efficiency in growing beef cattle
Evaluation of the application opportunities of precision livestock farming (PLF) for water buffalo (Bubalus bubalis) breeding: SWOT analysis
Evaluation of the RumiWatch system as a benchmark to monitor feeding and locomotion behaviors of grazing dairy cows
Evaluation of the tri-axial accelerometer to identify and predict parturition-related activities of Debouillet ewes in an intensive setting
Evaluation of Wearable Cameras for Monitoring and Analyzing Calf Behavior: A Preliminary Study
Evaluation via Supervised Machine Learning of the Broiler Pectoralis Major and Liver Transcriptome in Association With the Muscle Myopathy Wooden Breast
Evaporative pad cooling model validation in a closed dairy cattle building
Evidence of vascular endothelial dysfunction in Wooden Breast disorder in chickens: Insights through gene expression analysis, ultra-structural evaluation and supervised machine learning methods
Exploration of machine learning models to predict the environmental and remote sensing risk factors of haemonchosis in sheep flocks of Rajasthan, India
Exploring machine learning algorithms for early prediction of clinical mastitis
Exploring Smart Glasses for Augmented Reality: A Valuable and Integrative Tool in Precision Livestock Farming
Exploring the predictive capability of machine learning models in identifying foot and mouth disease outbreak occurrences in cattle farms in an endemic setting of Thailand
Extensive Sheep and Goat Production: The Role of Novel Technologies towards Sustainability and Animal Welfare
Extraction of key regions of beef cattle based on bidirectional tomographic slice features from point cloud data
Facial Recognition of Dairy Cattle Based on Improved Convolutional Neural Network
Factors Affecting Grazing and Rumination Behaviours of Dairy Cows in a Pasture-Based System in New Zealand
Factors associated with estrous expression and subsequent fertility in lactating dairy cows using automated activity monitoring
Factors associated with milking-to-milking variability in somatic cell counts from healthy cows in an automatic milking system
Farmers' representations of the effects of precision livestock farming on human-animal relationships
Farming smarter with big data: Insights from the case of Australia's national dairy herd milk recording scheme

Farm-level factors associated with lameness prevalence, productivity, and milk quality in farms with automated milking systems
Fast and Non-Destructive Quail Egg Freshness Assessment Using a Thermal Camera and Deep Learning-Based Air Cell Detection Algorithms for the Revalidation of the Expiration Date of Eggs
Fast Pig Detection with a Top-View Camera under Various Illumination Conditions
Fatty Acid Profiles from Routine Milk Recording as a Decision Tool for Body Weight Change of Dairy Cows after Calving
Feasibility of Real-Time Conditional Sacral Neuromodulation Using Wireless Bladder Pressure Sensor
Feasibility of the use of deep learning classification of teat-end condition in Holstein cattle
Feasibility Study: Improving Floor Cleanliness by Using a Robot Scraper in Group-Housed Pregnant Sows and Their Reactions on the New Device
Feather Damage Monitoring System Using RGB-Depth-Thermal Model for Chickens
Feature detection method for hind leg segmentation of sheep carcass based on multi-scale dual attention U-Net
Feed weight estimation model for health monitoring of meat rabbits based on deep learning
Feeding behavior parameters and temporal patterns in mid-lactation Holstein cows across a range of residual feed intake values
Fetal Cardiovascular Decompensation During Labor Predicted From the Individual Heart Rate Tracing: A Machine Learning Approach in Near-Term Fetal Sheep Model
Field-Applicable Pig Anomaly Detection System Using Vocalization for Embedded Board Implementations
Flock Nocturnal Activity: Is There a Rotative Guard?
FLYOLOv3 deep learning for key parts of dairy cow body detection
Footpad Monitoring: Reliability of an Automated System to Assess Footpad Dermatitis in Turkeys (Meleagris gallopavo) During Slaughter
Forage mass estimation in a mixed pasture by machine learning, pasture management and satellite meteorological data
Foraging behaviour of gestating sows on pasture and damages to vegetation cover are influenced by restriction of concentrate feed
Forecasting chronic mastitis using automatic milking system sensor data and gradient-boosting classifiers
Forecasting Milking Efficiency of Dairy Cows Milked in an Automatic Milking System Using the Decision Tree Technique
Forecasting the milk yield of cows on farms equipped with automatic milking system with the use of decision trees
FPDP: Flexible Privacy-Preserving Data Publishing Scheme for Smart Agriculture
Free-range laying hens: using technology to show the dynamics and impact of hen movement
Frequency analysis of the sneeze caused by swine influenza virus strains: Automatic

sneeze around-the-clock detection using a support vector machine
Frequency modulated continuous wave radar-based system for monitoring dairy cow respiration rate
Frequent range visits further from the shed relate positively to free-range broiler chicken welfare
From extra to actor: Facilitating automated conditioning in animal-robot interaction
Full model selection using regression trees for numeric predictions of biomarkers for metabolic challenges in dairy cows
Full-length 16S rRNA gene sequencing and machine learning reveal the bacterial composition of inhalable particles from two different breeding stages in a piggery
Fully Automated Deep Learning-based Sex Recognition in Pigs
Fusion of RGB, optical flow and skeleton features for the detection of lameness in dairy cows
Future of dairy farming from the Dairy Brain perspective: Data integration, analytics, and applications
Fuzzy system for assessing bovine fertility according to semen characteristics
Gait Analysis in Walking and Trotting Dairy Cows on Different Flooring Types with Novel Mobile Pressure Sensors and Inertial Sensors
Galyean Appreciation Club Review: revisiting nutrition and health of newly received cattle-what have we learned in the last 15 years?
Gastrointestinal nematode infection affects overall activity in young sheep monitored with tri-axial accelerometers
Gender determination of domestic chicks based on vocalization signals
Generating action plans for poultry management using artificial neural networks
Genes and regulatory mechanisms associated with experimentally-induced bovine respiratory disease identified using supervised machine learning methodology
Genetic analyses of novel temperament and milkability traits in Norwegian Red cattle based on data from automatic milking systems
Genetic and phenotypic variation and consistency in cow preference and circadian use of robotic milking units
Genetic Parameters Estimation of Milking Traits in Polish Holstein-Friesians Based on Automatic Milking System Data
Genetic relationship among somatic cell score and some milking traits in Holstein-Friesian primiparous cows milked by an automated milking system
Genetic, farm, and lactation effects on behavior and performance of US Holsteins in automated milking systems
Genetic-Chicken Swarm Algorithm for Minimizing Energy in Wireless Sensor Network
Genome-enabled classification of stayability in Nellore cattle under a machine learning framework
Genome-enabled prediction of reproductive traits in Nellore cattle using parametric models and machine learning methods
Genome-Wide Association Studies for Methane Production in Dairy Cattle
Genome-wide association studies of beef cow terrain-use traits using Bayesian multiple-

SNP regression
Genome-Wide Associative Study of Phenotypic Parameters of the 3D Body Model of Aberdeen Angus Cattle with Multiple Depth Cameras
Genomic merit for reproductive traits. I: Estrous characteristics and fertility in Holstein heifers
GIS-based approach for mapping the density and distribution of crossbred cattle
Goats are able to adapt to virtual fencing; A field study in commercial goat herds on Norwegian farms
Good prospects: high-resolution telemetry data suggests novel brood site selection behaviour in waterfowl
Goose Surface Temperature Monitoring System Based on Deep Learning Using Visible and Infrared Thermal Image Integration
GPS Coordinates for Modelling Correlated Herd Effects in Genomic Prediction Models Applied to Hanwoo Beef Cattle
GPS FOR COWS IN WESTERN AFRICA
GPS monitoring reveals circadian rhythmicity in free-grazing sheep
GPS Tracking to Monitor the Spatiotemporal Dynamics of Cattle Behavior and Their Relationship with Feces Distribution
Graduate Student Literature Review: Evaluating the appropriate use of wearable accelerometers in research to monitor lying behaviors of dairy cows
Graph Synthesis for Pig Breed Classification From Muzzle Images
Grassland Ecological Protection Monitoring and Management Application Based on ZigBee Wireless Sensor Network
Grazing Cow Behavior's Association with Mild and Moderate Lameness
Group level and individual activity of broiler chickens hatched in 3 different systems
Group-housed pig detection in video surveillance of overhead views using multi-feature template matching
Groups and Individuals: Optical Flow Patterns of Broiler Chicken Flocks Are Correlated with the Behavior of Individual Birds
HEAT LOAD-INDUCED CHANGES IN LYING BEHAVIOR AND LYING CUBICLE OCCUPANCY OF LACTATING DAIRY COWS IN A NATURALLY VENTILATED BARN
Heat stress and feeding behaviour of dairy cows in late lactation
Heat Stress Impacts on Lactating Cows Grazing Australian Summer Pastures on an Automatic Robotic Dairy
Heat stress in a temperate climate leads to adapted sensor-based behavioral patterns of dairy cows
Heifers don't care: no evidence of negative impact on animal welfare of growing heifers when using virtual fences compared to physical fences for grazing
Heifers with positive genetic merit for fertility traits reach puberty earlier and have a greater pregnancy rate than heifers with negative genetic merit for fertility traits
Herding by caging: a formation-based motion planning framework for guiding mobile agents

Hidden Markov and Semi-Markov Models When and Why are These Models Useful for Classifying States in Time Series Data?
High Precision Classification of Resting and Eating Behaviors of Cattle by Using a Collar-Fitted Triaxial Accelerometer Sensor
High precision real-time location estimates in a real-life barn environment using a commercial ultra wideband chip
High real-time reporting of domestic and wild animal diseases following rollout of mobile phone reporting system in Kenya
High sensitivity, fast response and anti-interference crack-based reduced graphene oxide strain sensor for pig acoustic recognition
High-accuracy and energy-efficient wearable device for dairy cows' localization and activity detection using low-cost IMU/RFID sensors
High-precision scanning system for complete 3D cow body shape imaging and analysis of morphological traits
High-Resolution Gridded Livestock Projection for Western China Based on Machine Learning
Holstein Cattle Face Re-Identification Unifying Global and Part Feature Deep Network with Attention Mechanism
Horse foraging behavior detection using sound recognition techniques and artificial intelligence
Hot topic: Automated assessment of reticulo-ruminal motility in dairy cows using 3-dimensional vision
Hot topic: Detecting digital dermatitis with computer vision
How Are Information Technologies Addressing Broiler Welfare? A Systematic Review Based on the Welfare Quality (R) Assessment
How do we feed grazing livestock in the future? A case for knowledge-driven grazing systems
How should the respiration rate be counted in cattle?
How the Selection of Training Data and Modeling Approach Affects the Estimation of Ammonia Emissions from a Naturally Ventilated Dairy Barn-Classical Statistics versus Machine Learning
How to Predict Parturition in Cattle? A Literature Review of Automatic Devices and Technologies for Remote Monitoring and Calving Prediction
HRST: An Improved HRNet for Detecting Joint Points of Pigs
Hybrid Platform for Assessing Air Pollutants Released from Animal Husbandry Activities for Sustainable Livestock Agriculture
Identification and classification for sheep foraging behavior based on acoustic signal and deep learning
Identification of aflatoxin-poisoned broilers based on accelerometer and machine learning
Identification of body size characteristic points based on the Mask R-CNN and correlation with body weight in Ujumqin sheep
Identification of Buffalo Breeds Using Self-Activated-Based Improved Convolutional

Neural Networks
Identification of Changes in Rumination Behavior Registered with an Online Sensor System in Cows with Subclinical Mastitis
Identification of discriminating behavioural and movement variables in lameness scores of dairy cows at pasture from accelerometer and GPS sensors using a Partial Least Squares Discriminant Analysis
Identification of eggshell crack for hen egg and duck egg using correlation analysis based on acoustic resonance method
Identification of grass growth conditions based on sheep grazing acoustic signals
Identification of possible cow grazing behaviour indicators for restricted grass availability in a pasture-based spring calving dairy system
Identification of Predictor Genes for Feed Efficiency in Beef Cattle by Applying Machine Learning Methods to Multi-Tissue Transcriptome Data
Identification of Risk Factors for Lameness Detection with Help of Biosensors
Identification of Target Chicken Populations by Machine Learning Models Using the Minimum Number of SNPs
Identification of the Rumination in Cattle Using Support Vector Machines with Motion-Sensitive Bolus Sensors
Identifying Early Indicators of Tail Biting in Pigs by Variable Selection Using Partial Least Squares Regression
Identifying gram-negative and gram-positive clinical mastitis using daily milk component and behavioral sensor data
Identifying Health Status in Grazing Dairy Cows from Milk Mid-Infrared Spectroscopy by Using Machine Learning Methods
Identifying Images of Dead Chickens with a Chicken Removal System Integrated with a Deep Learning Algorithm
Identifying livestock behavior patterns based on accelerometer dataset
Identifying Sheep Activity from Tri-Axial Acceleration Signals Using a Moving Window Classification Model
Identifying the Mating Posture of Cattle Using Deep Learning-Based Object Detection with Networks of Various Settings
Identifying-and-counting based monitoring scheme for pigs by integrating BLE tags and WBLCX antennas
Image Analysis and Computer Vision Applications in Animal Sciences: An Overview
Image analysis for individual identification and feeding behaviour monitoring of dairy cows based on Convolutional Neural Networks (CNN)
Image Classification and Automated Machine Learning to Classify Lung Pathologies in Deceased Feedlot Cattle
Image feature extraction via local binary patterns for marbling score classification in beef cattle using tree-based algorithms
Image processing strategies for pig liveweight measurement: Updates and challenges
Image Processing Technology Based on Internet of Things in Intelligent Pig Breeding
Imaging of pipeline irregularities using a PIG system based on reflection mode

ultrasonic sensors
Immunocastrated female pigs' social and feeding behaviour
Impact Evaluation of Score Classes and Annotation Regions in Deep Learning-Based Dairy Cow Body Condition Prediction
Impact of Body-worn Sensors on Broiler Chicken Behavior and Agonistic Interactions
Impact of genomic preselection on subsequent genetic evaluations with ssGBLUP using real data from pigs
Impact of Lameness on Attributes of Feeding Registered with Noseband Sensor in Fresh Dairy Cows
Impact of shearing sheep on feeding and behaviour during the pre-embarkment feedlot phase of live export
Impacts of Divergent Moving Drives on Energy Efficiency and Performance of Various AMS in Operative Conditions
Implementation of Computer-Vision-Based Farrowing Prediction in Pens with Temporary Sow Confinement
Implementation of Inertia Sensor and Machine Learning Technologies for Analyzing the Behavior of Individual Laying Hens
Implementation of Technical and Technological Progress in Dairy Production
Importance of humidity and temperature in breech strike of Merino sheep
Improved pig behavior analysis by optimizing window sizes for individual behaviors on acceleration and angular velocity data
Improved ResNet-50 deep learning algorithm for identifying chicken gender
Improvement of conception rate on Hanwoo; The key hormones and novel estrus detector
Improvement of feed and nutrient efficiency in pig production through precision feeding
Improving genomic prediction accuracy for meat tenderness in Nellore cattle using artificial neural networks
Improving Genomic Prediction with Machine Learning Incorporating TPE for Hyperparameters Optimization
Improving Ovine Behavioral Pain Diagnosis by Implementing Statistical Weightings Based on Logistic Regression and Random Forest Algorithms
Improving predictive performance on survival in dairy cattle using an ensemble learning approach
Improving the estimation of amino acid requirements to maximize nitrogen retention in precision feeding for growing-finishing pigs
Improving the precision and accuracy of animal population estimates with aerial image object detection
Improving the prediction rate of unusual behaviors of animal in a poultry using deep learning technique
Improving the Reliability of Photovoltaic and Wind Power Storage Systems Using Least Squares Support Vector Machine Optimized by Improved Chicken Swarm Algorithm
Improving the Reliability of Scale-Free Image Morphometrics in Applications with

Minimally Restrained Livestock Using Projective Geometry and Unsupervised Machine Learning
In pursuit of a better broiler: a comparison of the inactivity, behavior, and enrichment use of fast- and slower growing broiler chickens
In vivo prediction of abdominal fat and breast muscle in broiler chicken using live body measurements based on machine learning
Inclusion of features derived from a mixture of time window sizes improved classification accuracy of machine learning algorithms for sheep grazing behaviours
Increased air velocity in the lying area improves pen hygiene and reduces ammonia emissions from houses with partly slatted pens for growing/finishing pigs
Increased Ruminoreticular Temperature and Body Activity after Foot-and-Mouth Vaccination in Pregnant Hanwoo (Bos taurus coreanae) Cows
Increasing mud levels in a feedlot influences beef cattle behaviours but not preference for feedlot or pasture environments
Individual Beef Cattle Identification Using Muzzle Images and Deep Learning Techniques
Individual dairy cow identification based on lightweight convolutional neural network
Individual dairy cow management: achievements, obstacles and prospects
Individual differences in digesta retention and their relation to chewing in cattle-A pilot investigation
Individual feed intake and performance of finishing steers on ryegrass pasture supplemented with increasing amounts of corn using an automated feeding system
Individual identification of cashmere goats via method of fusion of multiple optimization
Individual identification of dairy cows based on convolutional neural networks
Individual identification of dairy cows based on deep learning and feature fusion
Individual identification of Holstein dairy cows based on detecting and matching feature points in body images
Individual Monitoring of Activity and Lameness in Conventional and Slower-Growing Breeds of Broiler Chickens Using Accelerometers
Individual Monitoring of Behavior to Enhance Productivity and Welfare of Animals in Small-Scale Intensive Cattle Grazing Systems
Individualised automated lameness detection in dairy cows and the impact of historical window length on algorithm performance
INDOOR CLIMATE AND ENERGY MODEL CALIBRATION WITH MONITORED DATA OF A NATURALLY VENTILATED DAIRY BARN IN A COLD CLIMATE
Induction of hypocalcaemia and evaluation of reticuloruminal motility using a three-axis accelerometer
Industry 3.5 to empower smart production for poultry farming and an empirical study for broiler live weight prediction
Industry 4.0 and Precision Livestock Farming (PLF): An up to Date Overview across Animal Productions
Industry 4.0 implementations: a systematic review of approaches and main

applicabilities in the broiler meat production chain
Inferring an animal's environment through biologging: quantifying the environmental influence on animal movement
Influence of environmental factors and parity on milk yield dynamics in barn-housed dairy cattle
Influence of Housing and Management on Claw Health in Swiss Dairy Goats
Influence of precision livestock farming on the environmental performance of intensive dairy goat farms
Information perception in modern poultry farming: A review
Information Technologies for Welfare Monitoring in Pigs and Their Relation to Welfare Quality(R)
Infrared imaging a new non-invasive machine learning technology for animal husbandry
Infrared Spectrometry as a High-Throughput Phenotyping Technology to Predict Complex Traits in Livestock Systems
Infrared thermography reveals surface body temperature changes during proestrus and estrus reproductive phases in Gyr heifers (Bos taurus indicus)
Infrared Thermography-A Non-Invasive Method of Measuring Respiration Rate in Calves
In-line near-infrared analysis of milk coupled with machine learning methods for the daily prediction of blood metabolic profile in dairy cattle
Inline Reticulorumen pH as an Indicator of Cows Reproduction and Health Status
Innovative PLF Tool to Assess Growing-Finishing Pigs' Welfare
Innovative use and efficiency test of subcutaneous transponders for electronic identification of water buffaloes
In-situ classification of cattle behavior using accelerometry data
Instrumented pig gait analysis: State-of-the-art
Insulin in the saliva of pigs: Validation of an automated assay and changes at different physiological conditions
Integrated Data Augmentation for Accelerometer Time Series in Behavior Recognition: Roles of Sampling, Balancing, and Fourier Surrogates
Integrated infrared thermography and accelerometer-based behavior logger as a hoof lesion identification tool in dairy cows with various foot diseases under subtropical climates
Integrated meta-omics reveals new ruminal microbial features associated with feed efficiency in dairy cattle
INTEGRATED NAVIGATION MODELS OF A MOBILE FODDER-PUSHING ROBOT BASED ON A STANDARDIZED COW HUSBANDRY ENVIRONMENT
Integrating blockchain and the internet of things in precision agriculture: Analysis, opportunities, and challenges
Integrating diverse data sources to predict disease risk in dairy cattle-a machine learning approach
Integrating heterogeneous across-country data for proxy-based random forest

prediction of enteric methane in dairy cattle
Integrating livestock management and telemetry data to assess disease transmission risk between wildlife and livestock
Integrating the RFID identification system for Charolaise breeding bulls with 3D imaging for virtual archive creation
Integration of statistical inferences and machine learning algorithms for prediction of metritis cure in dairy cows
Integrative Systems Biology Analysis Elucidates Mastitis Disease Underlying Functional Modules in Dairy Cattle
Intelligent Grazing UAV Based on Airborne Depth Reasoning
Intelligent perception for cattle monitoring: A review for cattle identification, body condition score evaluation, and weight estimation
Intelligent Perception-Based Cattle Lameness Detection and Behaviour Recognition: A Review
Intelligent Technologies and Robotic Means in Agricultural Production
Inter- and intra-individual variability of feeding behaviour in group housed dairy goats
Interactions between Ewes and Rams during Mating Can Be Used to Predict Lambing Dates Accurately, but Not Sire
Internet of Things and Machine Learning techniques in poultry health and welfare management: A systematic literature review
Interpretable machine learning applied to on-farm biosecurity and porcine reproductive and respiratory syndrome virus
Intravaginal Devices and GNSS Collars with Satellite Communication to Detect Calving Events in Extensive Beef Production in Northern Australia
Introductory overview: Systems and control methods for operational management support in agricultural production systems
Inverted F Type Antenna Design for the Cattle Activity and Estrus Detection Sensor Module
Investigating off-grid systems for a mobile automated milking facility
Investigating perceptions, adoption, and use of digital technologies in the Canadian beef industry
Investigation of body secretions as bioindicators in cattle estrus detection
Invited review: Big Data in precision dairy farming
Invited review: Cattle lameness detection with accelerometers
Invited review: Hygienic quality, composition, and technological performance of raw milk obtained by robotic milking of cows
Invited review: Sensor technologies for real-time monitoring of the rumen environment
Invited review: The future of selection decisions and breeding programs: What are we breeding for, and who decides?
Invited review: Toward a common language in data-driven mastitis detection research
IoT device-based data acquisition system with on-board computation of variables for cow behaviour recognition
IoT for Development of Smart Dairy Farming

IoT-based measurement system for classifying cow behavior from tri-axial accelerometer
IoT-Based pH monitoring for detection of rumen acidosis
IoT-Blockchain Enabled Optimized Provenance System for Food Industry 4.0 Using Advanced Deep Learning
Is Continuous Heart Rate Monitoring of Livestock a Dream or Is It Realistic? A Review
Is Seeing Still Believing? Leveraging Deepfake Technology for Livestock Farming
Is Virtual Fencing an Effective Way of Enclosing Cattle? Personality, Herd Behaviour and Welfare
Japanese Black cattle call patterns classification using multiple acoustic features and machine learning models
Joint Models to Predict Dairy Cow Survival from Sensor Data Recorded during the First Lactation
Keel bone differences in laying hens housed in enriched colony cages
Keel impacts and associated behaviors in laying hens
Kernel density estimation analyses based on a low power-global positioning system for monitoring environmental issues of grazing cattle
Keys to innovation in animal science: genomics, big data and collaboration
Kinematic gait characteristics of straight line walk in clinically sound dairy cows
Kinetic effect of different ground conditions on the sole of the claws of standing and walking dairy cows
Lactation curve model with explicit representation of perturbations as a phenotyping tool for dairy livestock precision farming
LA-DeepLab V3+: A Novel Counting Network for Pigs
Lamb Behaviors Analysis Using a Predictive CNN Model and a Single Camera
Lambs' live weight estimation using 3D images
Lameness and lying behavior in grazing dairy cows
Lameness changes the behavior of dairy cows: daily rank order of lying and feeding behavior decreases with increasing number of lameness indicators present in cow locomotion
Lameness Detection in Cows Using Hierarchical Deep Learning and Synchrosqueezed Wavelet Transform
Lameness detection of dairy cows based on the YOLOv3 deep learning algorithm and a relative step size characteristic vector
Lameness in dairy cows: farmer perceptions and automated detection technology
LAP-MALDI MS coupled with machine learning: an ambient mass spectrometry approach for high-throughput diagnostics
Large-Scale Phenotyping of Livestock Welfare in Commercial Production Systems: A New Frontier in Animal Breeding
Large-scale PRNP genotyping of small ruminants using an automated high-throughput MALDI-TOF MS assay
Learn to Train: Improving Training Data for a Neural Network to Detect Pecking Injuries in Turkeys

Learning patterns from time-series data to discriminate predictions of tail-biting, fouling and diarrhoea in pigs
Learning, health and productivity of group-housed sows conditioned to signal-feeding under realistic husbandry conditions
Legal Complexities of Animal Welfare in Australia: Do On-Animal Sensors Offer a Future Option?
Leveraging Available Resources and Stakeholder Involvement for Improved Productivity of African Livestock in the Era of Genomic Breeding
Leveraging Fog Analytics for Context-Aware Sensing in Cooperative Wireless Sensor Networks
Leveraging latent representations for milk yield prediction and interpolation using deep learning
Leveraging sequential information from multivariate behavioral sensor data to predict the moment of calving in dairy cattle using deep learning
Lightweight individual cow identification based on Ghost combined with attention mechanism
Linking activity-sensor data and physiology to improve dairy cow fertility
Live Pig-Weight Learning and Prediction Method Based on a Multilayer RBF Network
Live Weight Prediction of Cattle Based on Deep Regression of RGB-D Images
LiveCare: An IoT-Based Healthcare Framework for Livestock in Smart Agriculture
Live-pig-airway surface imaging and whole-pig CT at the Australian Synchrotron Imaging and Medical Beamline
Livestock classification and counting in quadcopter aerial images using Mask R-CNN
Livestock data-Is it there and is it FAIR? A systematic review of livestock farming datasets in Australia
Livestock Identification Using Deep Learning for Traceability
Livestock Informatics Toolkit: A Case Study in Visually Characterizing Complex Behavioral Patterns across Multiple Sensor Platforms, Using Novel Unsupervised Machine Learning and Information Theoretic Approaches
Livestock Management With Unmanned Aerial Vehicles: A Review
Livestock unit calculation: a method based on energy needs to refine the study of livestock farming systems
Livestock vocalisation classification in farm soundscapes
Locomotion behavior of dairy cows on traditional summer mountain farms in comparison with modern cubicle housing without access to pasture
Long-term and stable detection of H2S in a pig house at low operating temperature based on Ce2O3/In2O3 hollow microspheres with a remote monitoring system
Long-Term Measurement of Piglet Activity Using Passive Infrared Detectors
Long-Term Tracking of Group-Housed Livestock Using Keypoint Detection and MAP Estimation for Individual Animal Identification
Loop-mediated isothermal amplification (LAMP) and machine learning application for early pregnancy detection using bovine vaginal mucosal membrane
LoRa Communications as an Enabler for Internet of Drones towards Large-Scale

Livestock Monitoring in Rural Farms
Lost in translation-the use of remote and on-animal sensing for extensive livestock systems
Low-Cost Fluorescence Sensor for Ammonia Measurement in Livestock Houses
Low-Cost Livestock Global Positioning System Collar from Commercial Off-the-Shelf Parts
Lung Ultrasonography and Clinical Follow-Up Evaluations in Fattening Bulls Affected by Bovine Respiratory Disease (BRD) during the Restocking Period and after Tulathromycin and Ketoprofen Treatment
Lying behavior and activity during the transition period of clinically healthy grazing dairy cows
Lying behaviour of housed and outdoor-managed pregnant sheep
Lying behaviour of lactating dairy cows in a cow-calf contact freestall system
Machine learning algorithms for lamb survival
Machine learning algorithms for predicting peak yield in buffaloes using linear traits
Machine Learning Algorithms to Classify and Quantify Multiple Behaviours in Dairy Calves Using a Sensor: Moving beyond Classification in Precision Livestock
Machine learning algorithms, bull genetic information, and imbalanced datasets used in abortion incidence prediction models for Iranian Holstein dairy cattle
Machine learning application in growth and health prediction of broiler chickens
Machine Learning Applications for Precision Agriculture: A Comprehensive Review
Machine learning applied to transcriptomic data to identify genes associated with feed efficiency in pigs
Machine learning approach to integrated endometrial transcriptomic datasets reveals biomarkers predicting uterine receptivity in cattle at seven days after estrous
Machine Learning Approach to Predict Air Temperature and Relative Humidity inside Mechanically and Naturally Ventilated Duck Houses: Application of Recurrent Neural Network
Machine learning approaches for the prediction of lameness in dairy cows
Machine learning based fog computing assisted data-driven approach for early lameness detection in dairy cattle
Machine learning based personalized promotion strategy of piglets weaned per sow per year in large-scale pig farms
Machine Learning based Prediction of Insufficient Herbage Allowance with Automated Feeding Behaviour and Activity Data
Machine learning classification methods informing the management of inconclusive reactors at bovine tuberculosis surveillance tests in England
Machine learning classification of breeding protocol descriptions from Canadian Holsteins
Machine Learning in Agriculture: A Comprehensive Updated Review
Machine learning models for predicting the use of different animal breeding services in smallholder dairy farms in Sub-Saharan Africa
Machine Learning Prediction and Experimental Validation of Antigenic Drift in H3

Influenza A Viruses in Swine
Machine learning prediction of sleep stages in dairy cows from heart rate and muscle activity measures
Machine learning to detect behavioural anomalies in dairy cows under subacute ruminal acidosis
Machine Learning to Detect Posture and Behavior in Dairy Cows: Information from an Accelerometer on the Animal's Left Flank
Machine Learning-Based Co-Expression Network Analysis Unravels Potential Fertility-Related Genes in Beef Cows
Machine learning-based farm risk management: A systematic mapping review
Machine Learning-Based Live Weight Estimation for Hanwoo Cow
Machine Learning-Based Microclimate Model for Indoor Air Temperature and Relative Humidity Prediction in a Swine Building
Machine learning-driven dynamic risk prediction for highly pathogenic avian influenza at poultry farms in Republic of Korea: Daily risk estimation for individual premises
Machine-learning methods applied to integrated transcriptomic data from bovine blastocysts and elongating conceptuses to identify genes predictive of embryonic competence
Machine-Learning Techniques Can Enhance Dairy Cow Estrus Detection Using Location and Acceleration Data
Making sense in the cloud: Farm advisory services in a smart farming future
Making tiestalls more comfortable: II. Increasing chain length to improve the ease of movement of dairy cows
Making tiestalls more comfortable: III. Providing additional lateral space to improve the resting capacity and comfort of dairy cows
Mapping Industrial Poultry Operations at Scale With Deep Learning and Aerial Imagery
Mapping Soil and Pasture Attributes for Buffalo Management through Remote Sensing and Geostatistics in Amazon Biome
Mastitis detection with recurrent neural networks in farms using automated milking systems
Measurement Duration but Not Distance, Angle, and Neighbour-Proximity Affects Precision in Enteric Methane Emissions when Using the Laser Methane Detector Technique in Lactating Dairy Cows
Measurement of the three-axis vibration, temperature, and relative humidity profiles of commercial transport trailers for pigs
Measuring Comfort Behaviours in Laying Hens Using Deep-Learning Tools
Measuring executive function in sheep (Ovis aries) using visual stimuli in a semi-automated operant system
Measuring Farm Animal Emotions-Sensor-Based Approaches
Measuring lamb activity during lactation by actigraphy
Measuring liveweight changes in lactating dairy ewes with an automated walk-over-weighing system
Metabolomics meets machine learning: Longitudinal metabolite profiling in serum of

normal versus overconditioned cows and pathway analysis
Method Applied To Animal MonitoringThrough VANT Images
Methodological guidelines: Cow milk mid-infrared spectra to predict reference enteric methane data collected by an automated head-chamber system
Methodologies for Assessing Disease Tolerance in Pigs
Microdialysis and CO2 sensors detect pancreatic ischemia in a porcine model
Microfluidic E-tongue to diagnose bovine mastitis with milk samples using Machine learning with Decision Tree models
Milk losses and dynamics during perturbations in dairy cows differ with parity and lactation stage
Milk losses linked to mastitis treatments at dairy farms with automatic milking systems
Mind the Queue: A Case Study in Visualizing Heterogeneous Behavioral Patterns in Livestock Sensor Data Using Unsupervised Machine Learning Techniques
Miniaturized Electrochemical Sensors to Monitor Fetal Hypoxia and Acidosis in a Pregnant Sheep Model
Model research of the pig's microbiome based on "One Health" concept in the light of the shared human and animal health
Model selection for 24/7 pig position and posture detection by 2D camera imaging and deep learning
Modeling cow somatic cell count using sensor data as input to generalized additive models
Modeling gait score of broiler chicken via production and behavioral data
Modeling of Ambient Environment and Thermal Status Relationship of Pig's Body in a Pig Barn
Modeling of energy and emissions from animal manure using machine learning methods: the case of the Western Mediterranean Region, Turkey
Modeling reticular and ventral ruminal pH of lactating dairy cows using ingestion and rumination behavior
Modeling the influence of livestock grazing pressure on grassland bird distributions
Modeling variability of the lactation curves of cows in automated milking systems
Modelling and Validation of Computer Vision Techniques to Assess Heart Rate, Eye Temperature, Ear-Base Temperature and Respiration Rate in Cattle
Modelling methane emissions from pig manure using statistical and machine learning methods
Modelling of heat stress in a robotic dairy farm. Part 2: Identifying the specific thresholds with production factors
Modelling the domestic poultry population in the United States: A novel approach leveraging remote sensing and synthetic data methods
Modelling the shape of the pig scapula
Modern livestock farming under tropical conditions using sensors in grazing systems
Modern Virtual Fencing Application: Monitoring and Controlling Behavior of Goats Using GPS Collars and Warning Signals
Monitoring Behaviors of Broiler Chickens at Different Ages with Deep Learning

Monitoring body temperature of cattle using an innovative infrared photodiode thermometer
Monitoring estrous activity in pasture-based dairy cows
Monitoring of Cow Location in a Barn by an Open-Source, Low-Cost, Low-Energy Bluetooth Tag System
Monitoring of the core body temperature of cows using implantable wireless thermometers
MONITORING POLLUTION LEVEL AND MICROCLIMATE CONDITIONS IN A NATURALLY VENTILATED LIVESTOCK BUILDING USING OPEN-SOURCE DEVICE
Monitoring predictive and informative indicators of the energy status of dairy cows during early lactation in the context of monthly milk recordings using mid-infrared spectroscopy
Monitoring serologic response to single in ovo vaccination with an immune complex vaccine against infectious bursal disease in broilers
MOOnitor: An IoT based multi-sensory intelligent device for cattle activity monitoring
Mortality prediction of laying hens due to heat waves
Motivations and attitudes of Brazilian dairy farmers regarding the use of automated behaviour recording and analysis systems
Mounting Behaviour Recognition for Pigs Based on Deep Learning
Moving mean-based algorithm for dairy cow's oestrus detection from uniaxial-accelerometer data acquired in a free-stall barn
Multi-breed genome-wide association studies across countries for electronically recorded behavior traits in local dual-purpose cows
Multi-Center Agent Loss for Visual Identification of Chinese Simmental in the Wild
Multi-country metabolic signature discovery for chicken health classification
Multicow pose estimation based on keypoint extraction
Multi-Pig Part Detection and Association with a Fully-Convolutional Network
Multiple Country Approach to Improve the Test-Day Prediction of Dairy Cows' Dry Matter Intake
Multiview Eye Localisation to Measure Cattle Body Temperature Based on Automated Thermal Image Processing and Computer Vision
Multi-view real-time acquisition and 3D reconstruction of point clouds for beef cattle
Natural versus induced estrus indicators of Holstein heifers determined by an automated activity monitoring system
Network Architecture for Intelligent Identification of Faults in Rabbit Farm Environment Monitoring Based on a Biological Neural Network Model
NEURO-FUZZY MODELING OF EYEBALL AND CREST TEMPERATURES IN EGG-LAYING HENS
Neutron activation analysis and data mining techniques to discriminate between beef cattle diets
New insights in improving sustainability in meat production: opportunities and challenges

New Insights into the Hourly Manure Coverage Proportion on the Manure Belt in a Typical Layer House for Accurate Ammonia Emission Modeling
New method to automatically evaluate the sexual activity of the ram based on accelerometer records
New opportunities in animal breeding due to increasing digitization
Nitrogen variability assessment of pasture fields under an integrated crop-livestock system using UAV, PlanetScope, and Sentinel-2 data
No-contact microchip measurements of body temperature and behavioural changes prior to foaling
Nocturnal chats of farmed animals: non-rutting vocalisations of male Iberian red deer, Cervus elaphus hispanicus
Non-Coding Transcriptome Provides Novel Insights into the Escherichia coli F17 Susceptibility of Sheep Lamb
Non-Destructive Detection of Male and Female Information of Early Duck Embryos Based on Visible/Near Infrared Spectroscopy and Deep Learning
Nondestructive Detection of Microcracks in Poultry Eggs Based on the Electrical Characteristics Model
Noninferiority study evaluating the efficacy of a teat disinfectant containing copper and zinc for prevention of naturally occurring intramammary infections in an automatic milking system
Noninvasive cardiac output monitoring in a porcine model using the inspired sinewave technique: a proof-of-concept study
Non-Invasive Cattle Body Temperature Measurement Using Infrared Thermography and Auxiliary Sensors
Non-invasive measure of heat stress in sheep using machine learning techniques and infrared thermography
Non-Invasive Methods of Quantifying Heat Stress Response in Farm Animals with Special Reference to Dairy Cattle
Non-Invasive Sheep Biometrics Obtained by Computer Vision Algorithms and Machine Learning Modeling Using Integrated Visible/Infrared Thermal Cameras
Noseband sensor validation and behavioural indicators for assessing beef cattle grazing on extensive pastures
Novel method for real-time detection and tracking of pig body and its different parts
Novel prediction models for hyperketonemia using bovine milk Fourier-transform infrared spectroscopy
Novel ways to use sensor data to improve mastitis management
Nuclear Magnetic Resonance (NMR)-Based Quantification on Flavor-Active and Bioactive Compounds and Application for Distinguishment of Chicken Breeds
Object detection and tracking using a high-performance artificial intelligence-based 3D depth camera: towards early detection of African swine fever
Objective scoring of footpad dermatitis in broiler chickens using image segmentation and a deep learning approach: camera-based scoring system
Occlusion-Based Coordination Protocol Design for Autonomous Robotic Shepherding

Tasks
Occupational Safety and Health with Technological Developments in Livestock Farms: A Literature Review
Occurrence and greater intensity of estrus in recipient lactating dairy cows improve pregnancy per embryo transfer
Omics technologies in poultry health and productivity - part 2: future applications in the poultry industry
On farm automatic sheep breed classification using deep learning
On the Development of a Wearable Animal Monitor
On the use of on-cow accelerometers for the classification of behaviours in dairy barns
On-Barn Forecasting Beef Cattle Production Based on Automated Non-Contact Body Measurement System
One-Shot Learning with Pseudo-Labeling for Cattle Video Segmentation in Smart Livestock Farming
On-farm detection of claw lesions in dairy cows based on acoustic analyses and machine learning
On-farm use of disease alerts generated by precision dairy technology
On-farm welfare monitoring system for goats based on Internet of Things and machine learning
Online estimating weight of white Pekin duck carcass by computer vision
Online forecasting of daily feed intake in lactating sows supported by offline time-series clustering, for precision livestock farming
Online milk composition analysis with an on-farm near-infrared sensor
On-line monitoring of egg freshness using a portable NIR spectrometer in tandem with machine learning
On-line weight estimation of broiler carcass and cuts by a computer vision system
Ontology-Based IoT Middleware Approach for Smart Livestock Farming toward Agriculture 4.0: A Case Study for Controlling Thermal Environment in a Pig Facility
Opportunities to Apply Precision Livestock Management on Rangelands
Opportunities to Harness High-Throughput and Novel Sensing Phenotypes to Improve Feed Efficiency in Dairy Cattle
Opportunities to Improve Resilience in Animal Breeding Programs
Optical flow, behaviour and broiler chicken welfare in the UK and Switzerland
Optimal Placement of Laying Hen House Temperature Sensors Based on Genetic Algorithm
Optimising profitability and productivity of pasture-based dairy farms with automatic milking systems
Optimization and comparison of models for core temperature prediction of mother rabbits using infrared thermography
Optimization of Pig Positioning Method Based on Uniaxial Magnetoresistive Sensor
Optimization on multi-object tracking and segmentation in pigs' weight measurement
Optimized Deep-Learning-Based Method for Cattle Udder Traits Classification
Optimizing management of dairy goat farms through individual animal data

interpretation: A case study of smart farming in Spain
Oral hydration therapy with water and bovine respiratory disease incidence affects rumination behavior, rumen pH, and rumen temperature in high-risk, newly received beef calves
Outdoor animal tracking combining neural network and time-lapse cameras
Outlier detection in animal multivariate trajectories
Over 20 Years of Machine Learning Applications on Dairy Farms: A Comprehensive Mapping Study
Pain Management in Farm Animals: Focus on Cattle, Sheep and Pigs
Panoptic Segmentation of Individual Pigs for Posture Recognition
Paper-Based Progesterone Sensor Using an Allosteric Transcription Factor
Parasitic mites alter chicken behaviour and negatively impact animal welfare
Passive radio frequency identification and video tracking for the determination of location and movement of broilers
Pasture Access Affects Behavioral Indicators of Wellbeing in Dairy Cows
Pecking activity detection in group-housed turkeys using acoustic data and a deep learning technique
Perception of laying hen farmers, poultry veterinarians, and poultry experts regarding sensor-based continuous monitoring of laying hen health and welfare
Performance and optimization of an ear tag automated activity monitor for estrus prediction in dairy heifers
Performance and Usability of Smartglasses for Augmented Reality in Precision Livestock Farming Operations
Performance evaluation of leg and ear numbers in radio frequency identification systems (RFID) in sensitive livestock products in goat breeding
Performance of Milking Machine at Different Vacuum Levels in Crossbred Dairy Cows Milked in Automated Herringbone Parlour
Performance of Online Somatic Cell Count Estimation in Automatic Milking Systems
Peri-estrus activity and rumination time and its application to estrus prediction: Evidence from dairy herds under organic grazing and low-input conventional production
Personality and predictability in farmed calves using movement and space-use behaviours quantified by ultra-wideband sensors
Perspectives of digital agriculture in diverse types of livestock supply chain systems. Making sense of uses and benefits
PERTINENCE CURVES IN FUZZY MODELING OF THE PRODUCTIVE RESPONSES OF BROILERS
Phenomics for sustainable production in the South African dairy and beef cattle industry
Physical and economic comparison of pasture-based automatic and conventional milking systems
Pig Face Recognition Based on Metric Learning by Combining a Residual Network and Attention Mechanism
PIG FACE RECOGNITION MODEL BASED ON A CASCADED NETWORK

Pig Weight and Body Size Estimation Using a Multiple Output Regression Convolutional Neural Network: A Fast and Fully Automatic Method
PigLeg: prediction of swine phenotype using machine learning
Pig-Posture Recognition Based on Computer Vision: Dataset and Exploration
PigTalk: An AI-Based IoT Platform for Piglet Crushing Mitigation
Placental oxygen transfer reduces hypoxia-reoxygenation swings in fetal blood in a sheep model of gestational sleep apnea
Plasma concentrations of progesterone in the preceding estrous cycle are associated with the intensity of estrus and fertility of Holstein cows
Playing Behavior Classification of Group-Housed Pigs Using a Deep CNN-LSTM Network
Plumage damage in laying hens - automated detection in practical test -technical note
Potential for autonomous detection of lambing using global navigation satellite system technology
Practical Aspects of Weight Measurement Using Image Processing Methods in Waterfowl Production
Practical challenges and potential approaches to predicting low-incidence diseases on farm using individual cow data: A clinical mastitis example
Practical Experiences of a Smart Livestock Location Monitoring System Leveraging GNSS, LoRaWAN and Cloud Services
Practical Monitoring of Undergrown Pigs for IoT-Based Large-Scale Smart Farm
Practices and Applications of Convolutional Neural Network-Based Computer Vision Systems in Animal Farming: A Review
Prandial Correlations and Structure of the Ingestive Behavior of Pigs in Precision Feeding Programs
PreciseEdge raster RGB image segmentation algorithm reduces user input for livestock digital body measurements highly correlated to real-world measurements
Precision Agriculture for Crop and Livestock Farming-Brief Review
Precision Agriculture Technologies for Crop and Livestock Production in the Czech Republic
Precision dairy farming: Opportunities and challenges for India
Precision Detection of Real-Time Conditions of Dairy Cows Using an Advanced Artificial Intelligence Hub
Precision Feeding in Ecological Pig-Raising Systems with Maize Silage
Precision feeding of gestating sows: considering health, welfare and environmental conditions
Precision livestock agriculture and productive efficiency: The case of milk recording in Ireland
Precision livestock farming : new information useful for decision-making ?
Precision Livestock Farming and animal welfare: is the numerical revolution of agriculture able to take into account animals' and farmers' needs?
Precision Livestock Farming Applications (PLF) for Grazing Animals
Precision Livestock Farming in Swine Welfare: A Review for Swine Practitioners

Precision livestock farming technologies: Novel direction of information flow
Precision livestock farming: potential use in water buffalo (Bubalus bubalis) operations
Precision livestock farming: real-time estimation of daily protein deposition in growing-finishing pigs
Precision livestock feeding: matching nutrient supply with nutrient requirements of individual animals
Precision pig feeding: a breakthrough toward sustainability
Precision technologies for dairy calves and management applications
Precision Technologies to Address Dairy Cattle Welfare: Focus on Lameness, Mastitis and Body Condition
PreCowKetosis: A Shiny web application for predicting the risk of ketosis in dairy cows using prenatal indicators
Predicting body weight in growing pigs from feeding behavior data using machine learning algorithms
Predicting bovine tuberculosis status of dairy cows from mid-infrared spectral data of milk using deep learning
Predicting carcass cut yields in cattle from digital images using artificial intelligence
Predicting Cattle Grazing Behavior on Rangeland using Accelerometers
Predicting dairy cattle heat stress using machine learning techniques
Predicting dry matter intake in Canadian Holstein dairy cattle using milk mid-infrared reflectance spectroscopy and other commonly available predictors via artificial neural networks
Predicting dry matter intake in mid-lactation Holstein cows using point-in-time data streams available on dairy farms
Predicting feed intake using modelling based on feeding behaviour in finishing beef steers
Predicting first test day milk yield of dairy heifers
Predicting Growth and Carcass Traits in Swine Using Microbiome Data and Machine Learning Algorithms
Predicting hatchability of layer breeders and identifying effects of animal related and environmental factors
Predicting livestock behaviour using accelerometers: A systematic review of processing techniques for ruminant behaviour prediction from raw accelerometer data
Predicting methane emission in Canadian Holstein dairy cattle using milk mid-infrared reflectance spectroscopy and other commonly available predictors via artificial neural networks
Predicting pen fouling in fattening pigs from pig position
Predicting pregnancy status from mid-infrared spectroscopy in dairy cow milk using deep learning
Predicting ribeye area and circularity in live calves through 3D image analyses of body surface
Predicting Subclinical Ketosis in Dairy Cows Using Machine Learning Techniques
Predicting the 305-Day Milk Yield of Holstein-Friesian Cows Depending on the

Conformation Traits and Farm Using Simplified Selective Ensembles
Predicting the body weight of Balochi sheep using a machine learning approach
Predicting the current and future risk of ticks on livestock farms in Britain using random forest models
Predicting the Feed Intake of Cattle Based on Jaw Movement Using a Triaxial Accelerometer
Predicting the milk yield curve of dairy cows in the subsequent lactation period using deep learning
Predicting use of resources by dairy cows using time series
Prediction for Global Peste des Petits Ruminants Outbreaks Based on a Combination of Random Forest Algorithms and Meteorological Data
Prediction Model of Carbon Dioxide Concentration in Pig House Based on Deep Learning
Prediction models, assessment methodologies and biotechnological tools to quantify heat stress response in ruminant livestock
Prediction of 24-h and 6-h Periods before Calving Using a Multimodal Tail-Attached Device Equipped with a Thermistor and 3-Axis Accelerometer through Supervised Machine Learning
Prediction of Ammonia Concentration in a Pig House Based on Machine Learning Models and Environmental Parameters
Prediction of average daily gain of swine based on machine learning
Prediction of bite number and herbage intake by an accelerometer-based system in dairy sheep exposed to different forages during short-term grazing tests
Prediction of Complex Odor from Pig Barn Using Machine Learning and Identifying the Influence of Variables Using Explainable Artificial Intelligence
Prediction of Cow Calving in Extensive Livestock Using a New Neck-Mounted Sensorized Wearable Device: A Pilot Study
Prediction of deleterious mutations in coding regions of mammals with transfer learning
Prediction of first test day milk yield using historical records in dairy cows
Prediction of Girolando cattle weight by means of body measurements extracted from images
Prediction of growth in grower-finisher pigs using recurrent neural networks
Prediction of Hanwoo Cattle Phenotypes from Genotypes Using Machine Learning Methods
Prediction of Lactational Milk Yield of Cows Based on Data Recorded by AMS during the Periparturient Period
Prediction of lameness using automatically recorded activity, behavior and production data in post-parturient Irish dairy cows
Prediction of laying hen house odor concentrations using machine learning models based on small sample data
Prediction of litter performance in lactating sows using machine learning, for precision livestock farming

Prediction of metabolic status of dairy cows in early lactation with on-farm cow data and machine learning algorithms
PREDICTION OF OPTIMUM SUPPLEMENTAL HEAT FOR PIGLETS
Prediction of Polish Holstein?s economical index and calving interval using machine learning
Prediction of quarter level subclinical mastitis by combining in-line and on-animal sensor data
Prediction of Reproductive Success in Multiparous First Service Dairy Cows by Parameters from In-Line Sensors
Prediction of sheep carcass traits from early-life records using machine learning
Prediction of Tail Biting Events in Finisher Pigs from Automatically Recorded Sensor Data
Prediction of the diet energy digestion using kernel extreme learning machine: A case study with Holstein dry cows
Predictive Models of Dairy Cow Thermal State: A Review from a Technological Perspective
Predictive models to identify Holstein cows at risk of metritis and clinical cure and reproductive/productive failure following antimicrobial treatment
Predictive potential of activity and reticulo-rumen temperature variation for calving in Gyr heifers (Bos taurus indicus)
Pregnancy status predicted using milk mid-infrared spectra from dairy cattle
Pre-insemination prediction of dystocia in dairy cattle
Preliminary evaluation of a novel, fully automated, Telenostic device for rapid field-diagnosis of cattle parasites
Preliminary Experiment Using Sensors for Cow Health Monitoring after Surgical Treatment for the Left Displacement of the Abomasum
Preliminary study on the application of robotic herding to manipulation of grazing distribution: Behavioral response of cattle to herding by an unmanned vehicle and its manipulation performance
Prepartum behavior changes in dry Holstein cows at risk of postpartum diseases
Prepartum Fat Mobilization in Dairy Cows with Equal Body Condition and Its Impact on Health, Behavior, Milk Production and Fertility during Lactation
Pressure measurement in the reticulum to detect different behaviors of healthy cows
Pre-Weaned Calf Rearing on Northern Irish Dairy Farms: Part 1. A Description of Calf Management and Housing Design
Production planning decisions in the broiler chicken supply chain with growth uncertainty
Productive life span and resilience rank can be predicted from on-farm first-parity sensor time series but riot using a common equation across farms
PRODUCTIVE RESPONSES FROM BROILER CHICKENS RAISED IN DIFFERENT COMMERCIAL PRODUCTION SYSTEM - PART II: IMPACT OF CLIMATE CHANGE
Profiles of dairy cows with different productive lifespan emerge from multiple traits assessed at first lactation: the case of a grassland-based dairy system

Profiling Detection and Classification of Lameness Methods in British Dairy Cattle Research: A Systematic Review and Meta-Analysis
Progress and opportunities through use of genomics in animal production
Progression of different udder inflammation indicators and their episode length after onset of inflammation using automatic milking system sensor data
Progressive trends on the application of artificial neural networks in animal sciences-A review
Prospect and scope of artificial neural network in livestock farming: a review
Protein Supplementation and Grazing Behavior for Cows on Differing Late-Season Rangeland Grazing Systems
Proximal sensors for monitoring seasonal changes of feeding sites selected by grazing ewes
Proximity between horses in large groups in an open stable system - Analysis of spatial and temporal proximity definitions
Proximity Interactions in a Permanently Housed Dairy Herd: Network Structure, Consistency, and Individual Differences
Proximity sensors fitted to ewes and rams during joining can indicate the birth date of lambs
Proximity sensors provide an accurate alternative for measuring maternal pedigree of lambs in Australian sheep flocks under commercial conditions
Pullet Rearing Affects Collisions and Perch Use in Enriched Colony Cage Layer Housing
Quantifiable and feasible estrus detection using the ultrasonic sensor array and digital infrared thermography
Quantification of behavioural variation among sheep grazing on pasture using accelerometer sensors
Quantification of protein and phosphorus in livestock feed using mobile NMR sensor technology
Quantifying defence cascade responses as indicators of pig affect and welfare using computer vision methods
Quantifying the frequency and volume of urine deposition by grazing sheep using tri-axial accelerometers
Quantile regression forests-based modeling and environmental indicators for decision support in broiler farming
Quantitative measurement of udder oedema in dairy cows using ultrasound to monitor the effectiveness of diuretic treatment with furosemide
Quarter milking parameters by lactation in dairy cows
Radiated temperature from thermal imaging is related to feed consumption, growth rate and feed efficiency in grower pigs
Random Forest Modelling of Milk Yield of Dairy Cows under Heat Stress Conditions
Ranking of environmental heat stressors for dairy cows using machine learning algorithms
Rapid and Non-Destructive Monitoring of Moisture Content in Livestock Feed Using a Global Hyperspectral Model

Rapid turnover of sensor data to genetic evaluation for dairy cows in the cloud
Real-Time Behavioral Recognition in Dairy Cows Based on Geomagnetism and Acceleration Information
Real-Time Cattle Action Recognition for Estrus Detection
Real-Time Extensive Livestock Monitoring Using LPWAN Smart Wearable and Infrastructure
Real-Time Livestock Tracking System with Integration of Sensors and Beacon Navigation
Real-Time Measurement of the Daily Total Locomotor Behavior in Calves Reared in an Intensive Management System for the Possible Application in Precision Livestock Farming
Real-time modelling of individual weight response to feed supply for fattening pigs
Real-time modelling of indoor particulate matter concentration in poultry houses using broiler activity and ventilation rate
Real-Time Monitoring of Environmental Parameters in a Commercial Gestating Sow House Using a ZigBee-Based Wireless Sensor Network
Real-Time Monitoring of Self-Fed Supplement Intake, Feeding Behaviour, and Growth Rate as Affected by Forage Quantity and Quality of Rotationally Grazed Beef Cattle
Real-time sow behavior detection based on deep learning
Real-Time Standard Analysis of Disease Investigation (SADI)-A Toolbox Approach to Inform Disease Outbreak Response
Real-time temperature monitoring for the early detection of mastitis in dairy cattle: Methods and case researches
Real-time web-based microclimate monitoring of broiler chicken trucks on different shifts
Recent advances and future directions for uterine diseases diagnosis, pathogenesis, and management in dairy cows
Recent Advances on Early Detection of Heat Strain in Dairy Cows Using Animal-Based Indicators: A Review
Recent developments on rapid detection of main constituents in milk: a review
Recent progress in electrochemical biosensors as point of care diagnostics in livestock health
Recognising Cattle Behaviour with Deep Residual Bidirectional LSTM Model Using a Wearable Movement Monitoring Collar
Recognition and Classification of Broiler Droppings Based on Deep Convolutional Neural Network
Recognition and segmentation of individual pigs based on Swin Transformer
Recognition of aggressive behavior of group-housed pigs based on CNN-GRU hybrid model with spatio-temporal attention mechanism
Recognition of aggressive episodes of pigs based on convolutional neural network and long short-term memory
Recognition of Cattle's Feeding Behaviors Using Noseband Pressure Sensor With Machine Learning

Recognition of feeding behaviour of pigs and determination of feeding time of each pig by a video-based deep learning method
Recognition of Pantaneira cattle breed using computer vision and convolutional neural networks
Recognition of Pig Eating and Drinking Behavior Based on Visible Spectrum and YOLOv2
Recognizing lameness in dairy cattle herds Literature review
Record linkage for farm-level data analytics: Comparison of deterministic, stochastic and machine learning methods
Recording behaviour of indoor-housed farm animals automatically using machine vision technology: A systematic review
Recording cattle maternal behaviour using proximity loggers and tri-axial accelerometers
Recording the heart beat of cattle using a gradiometer system of optically pumped magnetometers
Reducing GPS Error for Smart Collars Based on Animal's Behavior
Reduction of Energy Intensity in Broiler Facilities: Methodology and Strategies
Reflected Light Spectrometry and AI-Based Data Analysis for Detection of Rapid Chicken Eggshell Change Caused by Mycoplasma Synoviae
Regional Assessment of Stocking Rate Effects on Uruguayan Rangelands: A Ranch-Level Study
Regularly fluctuating somatic cell count pattern in dairy herds
Relating Lying Behavior With Climate, Body Condition Score, and Milk Production in Dairy Cows
Relation of Automated Body Condition Scoring System and Inline Biomarkers (Milk Yield, beta-Hydroxybutyrate, Lactate Dehydrogenase and Progesterone in Milk) with Cow's Pregnancy Success
Relation of Subclinical Ketosis of Dairy Cows with Locomotion Behaviour and Ambient Temperature
Relationship between Circle of Willis Variations and Cerebral or Cervical Arteries Stenosis Investigated by Computer Tomography Angiography and Multitask Convolutional Neural Network
Relationship between metabolic status and behavior in dairy cows in week 4 of lactation
Relationship between Range Use and Fearfulness in Free-Range Hens from Different Rearing Enrichments
Relationship between Temperate Grass Sward Characteristics and the Grazing Behavior of Dairy Heifers
Relationship between the timing of insemination based on estrus detected by the automatic activity monitoring system and conception rates using sex-sorted semen in Holstein dairy cattle
Relationships among lying and standing behaviour, body condition score and milk production in primiparous cows
Relationships between Dairy Cows' Chewing Behavior with Forage Quality, Progress of

Lactation and Efficiency Estimates under Zero-Concentrate Feeding Systems
Relationships between Selected Physiological Factors and Milking Parameters for Cows Using a Milking Robot
Relationships between the incidence and degree of medullation with the diameter of alpaca fibers evaluated using a novel device based on artificial intelligence
Relationships of morphological and phototextural attributes of presumptive ovine zygotes and early embryos to their developmental competence in vitro: a preliminary assessment using time-lapse imaging
Remote monitoring system for livestock environmental information based on LoRa wireless ad hoc network technology
Remotely Sensed Imagery for Early Detection of Respiratory Disease in Pigs: A Pilot Study
Repeatability and Predictability of Calf Feeding Behaviors-Quantifying Between- and Within-Individual Variation for Precision Livestock Farming
Research and Development of Automatic Monitoring System for Livestock Farms
Research Note: Nondestructive detection of super grade chick embryos or hatchlings using near-infrared spectroscopy
Research on a Low-Cost, Open-Source, and Remote Monitoring Data Collector to Predict Livestock's Habits Based on Location and Auditory Information: A Case Study from Vietnam
Research on Chengdu Ma Goat Recognition Based on Computer Vison
Research on Innovative Business Plan. Smart Cattle Farming Using Artificial Intelligent Robotic Process Automation
Research on the lying pattern of grouped pigs using unsupervised clustering and deep learning
Research on Tracking and Identification of Typical Protective Behavior of Cows Based on DeepLabCut
Research Progress in the Early Warning of Chicken Diseases by Monitoring Clinical Symptoms
Response to dietary methionine supply of growing pigs fed daily tailored diets or fed according to a conventional phase feeding system
Resting behaviour of dairy cows under freestall or continuous grazed pasture conditions
Retinal Temperature Determination Based on Photopic Porcine Electroretinogram
Revealing the diversity in cattle behavioural response to high environmental heat using accelerometer-based ear tag sensors
Revealing the structure of the associations between housing system, facilities, management and welfare of commercial laying hens using Additive Bayesian Networks
Review of Sensor Technologies in Animal Breeding: Phenotyping Behaviors of Laying Hens to Select Against Feather Pecking
Review on implementation of IoT for environmental condition monitoring in the agriculture sector
Review: Anticipating alternative trajectories for responsible Agriculture 4.0 innovation in livestock systems

Review: Application and Prospective Discussion of Machine Learning for the Management of Dairy Farms
Review: Assessment of dairy cow welfare at pasture: measures available, gaps to address, and pathways to development of ad-hoc protocols
Review: Automated techniques for monitoring the behaviour and welfare of broilers and laying hens: towards the goal of precision livestock farming
Review: Environmental impact of livestock farming and Precision Livestock Farming as a mitigation strategy
Review: Precision Livestock Farming technologies in pasture-based livestock systems
Review: Precision livestock farming, automats and new technologies: possible applications in extensive dairy sheep farming
Review: Precision livestock farming: building 'digital representations' to bring the animals closer to the farmer
Review: Rumen sensors: data and interpretation for key rumen metabolic processes
Review: Sensor techniques in ruminants: more than fitness trackers
Review: Smart agri-systems for the pig industry
Review: Synergy between mechanistic modelling and data-driven models for modern animal production systems in the era of big data
RGB-D video-based individual identification of dairy cows using gait and texture analyses
Risk factors for delayed milk ejection in Holstein dairy cows milked 3 times per day
Road hogs: Implications from GPS collared feral swine in pastureland habitat on the general utility of road-based observation techniques for assessing abundance
Robot milking and relationship with culling rate in dairy cows
Robot utilisation of pasture-based dairy cows with varying levels of milking frequency
Robotic milking of dairy cows: a review
Rule Discovery in Milk Content towards Mastitis Diagnosis: Dealing with Farm Heterogeneity over Multiple Years through Classification Based on Associations
Rumen Fermentation Parameters Prediction Model for Dairy Cows Using a Stacking Ensemble Learning Method
Rumen temperature is a reliable proxy of core body temperature in sheep (Ovis aries)
Rumen-Reticular Temperature During Estrus and Ovulation Using Automated Activity Monitors in Dairy Cows
Rumination time around dry-off relative to the development of diseases in early-lactation cows
Salivary biomarkers to monitor stress due to aggression after weaning in piglets
Screening Discriminating SNPs for Chinese Indigenous Pig Breeds Identification Using a Random Forests Algorithm
Secure and Sustainable Framework for Cattle Recognition Using Wireless Multimedia Networks and Machine Learning Techniques
Secure LoRa Firmware Update with Adaptive Data Rate Techniques
Security Architecture for Swarms of Autonomous Vehicles in Smart Farming
Segmentation of body parts of cows in RGB-depth images based on template matching

Selection of Brucella abortus mimetic epitopes for fast diagnostic purposes in cattle
Selective Campylobacter detection and quantification in poultry: A sensor tool for detecting the cause of a common zoonosis at its source
Selective detection of ammonia by rGO decorated nanostructured ZnO for poultry and farm field applications
Selective sweep sites and SNP dense regions differentiate Mycobacterium bovis isolates across scales
SEMI-AUTOMATIC PIG WEIGHT ESTIMATION USING DIGITAL IMAGE ANALYSIS
Semi-Supervised Knowledge Distillation for Cross-Modal Hashing
Sensing solutions for improving the performance, health and wellbeing of small ruminants
Sensitivity and Specificity for the Detection of Clinical Mastitis by Automatic Milking Systems in Bavarian Dairy Herds
Sensitivity and specificity of a tail-activity measuring device for calving prediction in dairy cattle
Sensor and Video: Two Complementary Approaches for Evaluation of Dairy Cow Behavior after Calving Sensor Attachment
Sensor based disease detection: A case study using accelerometers to recognize symptoms of Bovine Ephemeral Fever
Sensor based eating time variables of dairy cows in the transition period related to the time to first service
Sensor based time budgets in commercial Dutch dairy herds vary over lactation cycles and within 24 hours
Sensor technology to support herd health monitoring: Using rumination duration and activity measures as unspecific variables for the early detection of dairy cows with health deviations
Sensor-based detection of parturition in beef cattle grazing in an extensive landscape: a case study using a commercial GNSS collar
Sensor-Based Detection of Predator Influence on Livestock: A Case Study Exploring the Impacts of Wild Dogs (Canis familiaris) on Rangeland Sheep
Separable Confident Transductive Learning for Dairy Cows Teat-End Condition Classification
Separate weighing of male and female broiler breeders by electronic platform weigher using camera technologies
Sequential air pollution emission estimation using a hybrid deep learning model and health-related ventilation control in a pig building
Serial Ultrasonographic and Real-Time Elastosonographic Assessment of the Ovine Common Calcaneal Tendon, after an Experimentally Induced Tendinopathy
Setup, Test and Validation of a UHF RFID System for Monitoring Feeding Behaviour of Dairy Cows
Sex Detection of Chicks Based on Audio Technology and Deep Learning Methods
Shank length and circumference measurement algorithm of breeder chickens based on extraction of regional key points

Sheep Counting Method Based on Multiscale Module Deep Neural Network
Sheep Identification Using a Hybrid Deep Learning and Bayesian Optimization Approach
Sheep lameness detection from individual hoof load
SHEEPFEARNET: Sheep fear test behaviors classification approach from video data based on optical flow and convolutional neural networks
Sheep's coping style can be identified by unsupervised machine learning from unlabeled data
Shepherding algorithm for heterogeneous flock with model-based discrimination
Shi-Tomasi corner detector for cattle identification from muzzle print image pattern
Short communication: Detection of mastication speed during rumination in cattle using 3-axis, neck-mounted accelerometers and fast Fourier transfer algorithm
Short communication: Effects of mammary biopsy in the dry period on activity and feeding behavior of dairy cows
Short communication: Evaluation of an eating time sensor for use in pasture-based dairy systems
Short communication: Greater intensity of estrous expression is associated with improved embryo viability from superovulated Holstein heifers
Short communication: Investigation of the temporal relationships between milk mid-infrared predicted biomarkers and lameness events in later lactation
Short Communication: The effect of age on young sheep biometric identification
Short-Term Adaptation of Dairy Cattle Production Parameters to Individualized Changes in Dietary Top Dress
Short-term feeding behaviour sound classification method for sheep using LSTM networks
Short-term fibre intake estimation in goats using surface electromyography of the masseter muscle
Short-term liveweight changes of dairy cows measured by stationary and walk-over weighing scales
SIFT-CNN Pipeline in Livestock Management: A Drone Image Stitching Algorithm
Signal level performance variation of radio frequency identification tags used in cow body
Simulation model of quarter milk flowrates to estimate quarter and cow milking duration and automated milking system's box duration
Simultaneous measurements of estrus behavior and plasma concentrations of estradiol during estrus in lactating and nonlactating dairy cows
Single-Channel sEMG Dictionary Learning Classification of Ingestive Behavior on Cows
Smart Animal Agriculture: Application of Real-Time Sensors to Improve Animal Well-Being and Production
Smart Animal Detection and Counting Framework for Monitoring Livestock in an Autonomous Unmanned Ground Vehicle Using Restricted Supervised Learning and Image Fusion
Smart farming in mountain areas: Investigating livestock farmers' technophobia and

technophilia and their perception of innovation
Smart Farming: An Enhanced Pursuit of Sustainable Remote Livestock Tracking and Geofencing Using IoT and GPRS
Smart Feeding Unit for Measuring the Pecking Force in Farmed Broilers
Smart Grazing in Tibetan Plateau: Development of a Ground-Air-Space Integrated Low-Cost Internet of Things System for Yak Monitoring
Smart Livestock Farming - An inventory
Smart poultry management: Smart sensors, big data, and the internet of things
Smart Technologies Lead to Smart Answers? On the Claim of Smart Sensing Technologies to Tackle Animal Related Societal Concerns in Europe Over Current Pig Husbandry Systems
SmartHerd management: A microservices-based fog computing-assisted IoT platform towards data-driven smart dairy farming
Social behaviour and transmission of lameness in a flock of ewes and lambs
Social density detection for suckling piglets based on convolutional neural network combined with local outlier factor algorithm
Social influence on the effectiveness of virtual fencing in sheep
SocialCattle: IoT-Based Mastitis Detection and Control Through Social Cattle Behavior Sensing in Smart Farms
SOLARIA-SensOr-driven resiLient and adaptive monitoRIng of farm Animals
Some challenges and opportunities for grazing dairy cows on temperate pastures
Sow Farrowing Early Warning and Supervision for Embedded Board Implementations
Spatial Distribution of Grazing Sites and Dung of Beef Cows in a Sloping Pasture
Spatial modeling of pigs' drinking patterns as an alarm reducing method I. Developing a multivariate dynamic linear model
Spatial modeling of pigs' drinking patterns as an alarm reducing method II. Application of a multivariate dynamic linear model
Spatiotemporal graph convolutional network for automated detection and analysis of social behaviours among pre-weaning piglets
Spatio-Temporal Semantic Data Model for Precision Agriculture IoT Networks
Spectral entropy of early-life distress calls as an iceberg indicator of chicken welfare
Stability Evaluation of Reference and Indicating Electrodes of pH Sensor during Monitoring of Cow's Rumen
StaticPigDet: Accuracy Improvement of Static Camera-Based Pig Monitoring Using Background and Facility Information
Statistical modeling of ruminal pH parameters from dairy cows based on a meta-analysis
Storing, combining and analysing turkey experimental data in the Big Data era
Strategy to Predict High and Low Frequency Behaviors Using Triaxial Accelerometers in Grazing of Beef Cattle
STRESS-FREE DETECTION TECHNOLOGIES FOR PIG GROWTH BASED ON WELFARE FARMING: A REVIEW
Structural impact of digitalisation in agriculture

Study of a QueryPNet Model for Accurate Detection and Segmentation of Goose Body Edge Contours
Study on Body Size Measurement Method of Goat and Cattle under Different Background Based on Deep Learning
Study on Poultry Pose Estimation Based on Multi-Parts Detection
Study on the Discrimination of Possible Error Sources That Might Affect the Quality of Volatile Organic Compounds Signature in Dairy Cattle Using an Electronic Nose
Subclinical nematode parasitism affects activity and rumination patterns in first-season grazing cattle
Suitability of milk lactate dehydrogenase and serum albumin for pathogen-specific mastitis detection in automatic milking systems
Suitability of rumination time during the first week after calving for detecting metabolic status and lactation performance in simmental dairy cows: a cluster-analytic approach
Suitability of somatic cell count, electrical conductivity, and lactate dehydrogenase activity in foremilk before versus after alveolar milk ejection for mastitis detection
Superhydrophobic Paper-Based Microfluidic Field-Effect Transistor Biosensor Functionalized with Semiconducting Single-Walled Carbon Nanotube and DNAzyme for Hypocalcemia Diagnosis
Supervised learning techniques for dairy cattle body weight prediction from 3D digital images
Supervised Machine Learning to Assess Methane Emissions of a Dairy Building with Natural Ventilation
Supplementing a blend of magnesium oxide to feedlot cattle: effects on ruminal, physiological, and productive responses
SUSTAINABLE APPROACH TO ENERGY EFFICIENCY: MITIGATION OF INDOOR MICROCLIMATE AIR PARAMETERS IN A NATURALLY VENTILATED LIVESTOCK BUILDING
Symposium review: Big data, big predictions: Utilizing milk Fourier-transform infrared and genomics to improve hyperketonemia management
Symposium review: Challenges and opportunities for evaluating and using the genetic potential of dairy cattle in the new era of sensor data from automation
Symposium review: Considerations for the future of dairy cattle housing: An animal welfare perspective
Symposium review: Dairy Brain-Informing decisions on dairy farms using data analytics
Symposium review: Real-time continuous decision making using big data on dairy farms
Symposium review: The most important factors affecting adoption of precision dairy monitoring technologies
Symposium review: Use of multiple biological, management, and performance data for the design of targeted reproductive management strategies for dairy cows
Systematic Literature Review of Models Used in the Epidemiological Analysis of Bovine Infectious Diseases
Systematic Review and Meta-Analysis: Identification of Factors Influencing Milking

Frequency of Cows in Automatic Milking Systems Combined with Grazing
Systems for evaluation of welfare on dairy farms
Taking the steps toward sustainable livestock: our multidisciplinary global farm platform journey
Teat detection algorithm: YOLO vs. Haar-cascade
Teat detection of dairy cows based on deep learning neural network FS-YOLOv4 model
Technical note: a nose ring sensor system to monitor dairy cow cardiovascular and respiratory metrics
Technical note: A novel approach to estimate dry matter intake of lactating dairy cows through multiple on-cow accelerometers
Technical note: Accelerometer-based recording of heavy breathing in lactating and dry cows as an automated measure of heat load
Technical note: Automatic evaluation of infrared thermal images by computerized active shape modeling of bovine udders challenged with Escherichia coli
Technical note: Calving prediction in dairy cattle based on continuous measurements of ventral tail base skin temperature using supervised machine learning
Technical note: Development of a noninvasive respiration rate sensor for cattle
Technical note: Interchangeability and comparison of methane measurements in dairy cows with 2 noninvasive infrared systems
Technical Note: Method to Streamline Processing of Livestock Global Positioning System Collar Data
Technical note: Random forests prediction of daily eating time of dairy cows from 3-dimensional accelerometer and radiofrequency identification
Technical note: using an automated head chamber system to administer an external marker to estimate fecal output by grazing beef cattle
Technical note: Validation of a behavior-monitoring collar's precision and accuracy to measure rumination, feeding, and resting time of lactating dairy cows
Technical note: validation of a system for monitoring individual behavior in beef heifers
Technical note: Validation of a system or monitoring individual behavior of Holstein cows
Technical note: Validation of an automated in-line milk progesterone analysis system to diagnose pregnancy in dairy cattle
Technical note: Validation of an ear-tag accelerometer to identify feeding and activity behaviors of tiestall-housed dairy cattle
Technological Tools for the Early Detection of Bovine Respiratory Disease in Farms
Technologies for the automated collection of heat stress data in sheep
Technologies monitoring and improving biosecurity compliance in barn anterooms
Technologies used at advanced dairy farms for optimizing the performance of dairy animals: A review
Technology adoption on farms: Using Normalisation Process Theory to understand sheep farmers' attitudes and behaviours in relation to using precision technology in flock management
Technology and responsibility: a discussion of underexamined risks and concerns in

Precision Livestock Farming
Temperature distribution in a finisher pig building with hybrid ventilation
Temporal aggregation network using micromotion features for early lameness recognition in dairy cows
Temporal Changes in Association Patterns of Cattle Grazing at Two Stocking Densities in a Central Arizona Rangeland
Temporary Exclusion of Cattle from a Riparian Zone Using Virtual Fencing Technology
Testing two NIRs instruments to predict chicken breast meat quality and exploiting machine learning approaches to discriminate among genotypes and presence of myopathies
The AMS Integrated Management Model: A decision-support system for automatic milking systems
The Application of Cameras in Precision Pig Farming: An Overview for Swine-Keeping Professionals
The application of micro-CT in egg-laying hen bone analysis: introducing an automated bone separation algorithm
The application of virtual fencing technology effectively herds cattle and sheep
The behavior of dairy cattle in late gestation: Effects of parity and dystocia
The behavior of dairy cattle in the transition period: Effects of blood calcium status
The behaviour of commercial broilers in response to a mobile robot
The complex adoption pathways of digital technology in Australian livestock supply chains systems
The costs of chronic mastitis: A simulation study of an automatic milking system farm
The detection of intramammary infections using online somatic cell counts
The development of sleep-like posture expression with age in female Holstein calves
The domestication triangle: How humans, animals and technology shape each other- The case of automated milking systems
The Early Prediction of Common Disorders in Dairy Cows Monitored by Automatic Systems with Machine Learning Algorithms
The economics of sensor-based management of dairy cow suboptimal mobility
The effect of age, environment and management on social contact patterns in sheep
The Effect of Behaviour and Diet on the Rumen Temperature of Holstein Bulls
The Effect of Grazing Intensity and Sward Heterogeneity on the Movement Behavior of Suckler Cows on Semi-natural Grassland
The effect of pasture quantity temporal variation on milking robot utilization
The Effect of Placement and Group Size on the Use of an Automated Brush by Groups of Lactating Dairy Cattle
The effect of stocking density and a blind on the behavior of Holstein dairy cows in group maternity pens. Part II: Labor length, lying behavior, and social behavior
The effect of temporal variation in feed quality and quantity on the diurnal feeding behaviour of dairy cows
The effects of a training program using a phantom to accustom heifers to the automatic milking system

The effects of cow introductions on milk production and behaviour of the herd measured with sensors
The estimation and interpretation of ordered logit models for assessing the factors connected with the productivity of Holstein-Friesian dairy cows in Egypt
The estimation of broiler respiration rate based on the semantic segmentation and video amplification
The Estimation of the Long-Term Agricultural Output with a Robust Machine Learning Prediction Model
The future of phenomics in dairy cattle breeding
The grades and freshness assessment of eggs based on density detection using machine vision and weighing sensor
The impact of alternative feeding strategies on total factor productivity growth of pig farming: Empirical evidence from EU countries
The Importance of Low Daily Risk for the Prediction of Treatment Events of Individual Dairy Cows with Sensor Systems
The influence of personality and weaning method on early feeding behavior and growth of Norwegian Red calves
The Internet of Things enhancing animal welfare and farm operational efficiency
The Interplay between the Internet of Things and agriculture: A bibliometric analysis and research agenda
The intraruminal redox potential is stabilised by opposing influences during fermentation
The livestock farming digital transformation: implementation of new and emerging technologies using artificial intelligence
The Na-x (SCN7A) channel: an atypical regulator of tissue homeostasis and disease
The need for national livestock surveillance in Pakistan
The new design of cows' behavior classifier based on acceleration data and proposed feature set
The optimal level of factors for high daily milk yield in automatic milking system
The political robot - The structural consequences of automated milking systems (AMS) in Norway
The potential of active and passive remote sensing to detect frequent harvesting of alfalfa
The potential of feeding patterns to assess generic welfare in growing-finishing pigs
The potential of volatile organic compound analysis in cervicovaginal mucus to predict estrus and ovulation in estrus-synchronized heifers
The Potentialities of Machine Learning for Cow-Specific Milking: Automatically Setting Variables in Milking Machines
The regulatory effect of herd structure on pig production under the environmental regulation
The relationship between dry period length and milk production of Holstein dairy cows in tropical climate: a machine learning approach
The relationship between gait and automated recordings of individual broiler activity

levels
The relationship between milk oestradiol concentrations and oestrus activity in lactating Holstein-Friesian cows
The relationships between udder-quarter somatic-cell counts and milk and milking parameters in cows managed with an automatic milking system
The Research Progress of Vision-Based Artificial Intelligence in Smart Pig Farming
The role of interoperable data standards in precision livestock farming in extensive livestock systems: A review
The role of precision agriculture in food security
The role of Precision Livestock Farming technologies in animal welfare monitoring: a review
The Segmented Colour Feature Extreme Learning Machine: Applications in Agricultural Robotics
The Sheep as a Comprehensive Animal Model to Investigate Interdependent Physiological Pressure Propagation and Multiparameter Influence on Cerebrospinal Fluid Dynamics
The use of accelerometers for the remote detection of mounting in rams and testosterone-treated wethers
The Use of Animal's Body, Scrotal Temperature and Motion Monitoring in Evaluating Boar Semen Production Capacity
The Use of Artificial Intelligence in Assessing Affective States in Livestock
The use of artificial neural networks for modelling rumen fill
The Use of Computer Records: A Tool to Increase Productivity in Dairy Herds
The Use of Infrared Thermography for the Monitoring of Udder Teat Stress Caused by Milking Machines
The use of integrated data collection system to evaluate milking performance, microclimatic condition and cows' behaviour
The Use of Multilayer Perceptron Artificial Neural Networks to Detect Dairy Cows at Risk of Ketosis
Thermal comfort and behavior of beef cattle in pasture-based systems monitored by visual observation and electronic device
Thermal comfort monitoring in aviaries by a real-time data acquisition system
Thermal Environment and Behavior Analysis of Confined Cows in a Compost Barn
Thermal environment of masonry-walled poultry house in the initial life stage of broilers
Thermal images to predict the thermal comfort index for Girolando heifers in the Brazilian semiarid region
Thermal imaging combined with predictive machine learning based model for the development of thermal stress level classifiers
Thermodynamics of standing and lying behavior in lactating dairy cows in freestall and parlor holding pens during conditions of heat stress
Three-Dimensional Simulation of the Temperature Distribution in a Commercial Broiler House

Threshold values to discriminate grazing activity of dairy cows by an uni-axial accelerometer as affected by grazing season and herbage mass
Timing of artificial insemination using fresh or frozen semen after automated activity monitoring of estrus in lactating dairy cows
Timing of eating during transition impacts feedlot cattle diet and liveweight gain
TinyCowNet: Memory- and Power-Minimized RNNs Implementable on Tiny Edge Devices for Lifelong Cow Behavior Distribution Estimation
T-LEAP: Occlusion-robust pose estimation of walking cows using temporal information
Toward the automated detection of behavioral changes associated with the post-weaning transition in pigs
Towards a Framework for High-Performance Simulation of Livestock Disease Outbreak: A Case Study of Spread of African Swine Fever in Vietnam
Towards a Revolutionized Agricultural Extension System for the Sustainability of Smallholder Livestock Production in Developing Countries: The Potential Role of ICTs
Towards a Vectorial Approach to Predict Beef Farm Performance
Towards an integrated animal health surveillance system in Tanzania: making better use of existing and potential data sources for early warning surveillance
Towards combining data prediction and internet of things to manage milk production on dairy cows
Towards Facial Expression Recognition for On-Farm Welfare Assessment in Pigs
Towards Machine Recognition of Facial Expressions of Pain in Horses
Towards modelling beef cattle management with Genetic Programming
Towards on-site automatic detection of noxious events in dairy cows
Towards re-identification for long-term tracking of group housed pigs
Towards the Estimation of Body Weight in Sheep Using Metaheuristic Algorithms from Biometric Parameters in Microsystems
Tracking and analysing social interactions in dairy cattle with real-time locating system and machine learning
Tracking and sensor-based detection of livestock water system failure: A case study simulation
Tracking group housed sows with an ultra-wideband indoor positioning system: A feasibility study
Tracking pecking behaviors and damages of cage-free laying hens with machine vision technologies
Tracking performance in poultry is affected by data cleaning method and housing system
Training and Validating a Machine Learning Model for the Sensor-Based Monitoring of Lying Behavior in Dairy Cows on Pasture and in the Barn
Transformer neural network to predict and interpret pregnancy loss from activity data in Holstein dairy cows
Transforming the Adaptation Physiology of Farm Animals through Sensors
Translactational associations of dry off management, milking activity, and somatic cell count in herds with automated milking systems

Trend and Representativeness of Acoustic Features of Broiler Chicken Vocalisations Related to CO2
Trends in Changes of Automatic Milking System Biomarkers and Their Relations with Blood Biochemical Parameters in Fresh Dairy Cows
Tumor localization using radio-frequency identification clip marker: experimental results of an ex vivo porcine model
Twelve Threats of Precision Livestock Farming (PLF) for Animal Welfare
Two-stage method based on triplet margin loss for pig face recognition
UBER: UAV-Based Energy-Efficient Reconfigurable Routing Scheme for Smart Wireless Livestock Sensor Network
Understanding the public attitudinal acceptance of digital farming technologies: a nationwide survey in Germany
Unraveling the Relationship between Milk Yield and Quality at the Test Day with Rumination Time Recorded by a PLF Technology
Unrest index for estimating thermal comfort of poultry birds (Gallus gallus domesticus) using computer vision techniques
Unsupervised automated monitoring of dairy cows' behavior based on Inertial Measurement Unit attached to their back
Use of a Global Positioning System (GPS) to Manage Extensive Sheep Farming and Pasture Land
Use of an ear-tag accelerometer and a radio-frequency identification (RFID) system for monitoring the licking behaviour in grazing cattle
Use of commercial somatic cell counters to quantify somatic cells in non-lactating bovine mammary gland secretions
Use of discriminant statistical procedures for an early detection of persistent lactations in dairy cows
Use of Mechanistic Nutrition Models to Identify Sustainable Food Animal Production
Use of Medria (R) monitoring collars to facilitate and optimize grazing management of dairy cows
Use of Predicted Behavior from Accelerometer Data Combined with GPS Data to Explore the Relationship between Dairy Cow Behavior and Pasture Characteristics
Use of radio frequency identification (RFID) technology to record grazing beef cattle water point use
Use of radio-frequency identification technology to assess the frequency of cattle visits to mineral feeders
Use of the Rumination Profile Through Collar Sensors for Mastitis Diagnosis in Dairy Cows
Use of validated objective methods of locomotion characteristics and weight distribution for evaluating the efficacy of ketoprofen for alleviating pain in cows with limb pathologies
Using 3D Imaging and Machine Learning to Predict Liveweight and Carcass Characteristics of Live Finishing Beef Cattle
Using a CNN-LSTM for basic behaviors detection of a single dairy cow in a complex

environment
Using an artificial neural network to predict the probability of oviposition events of precision-fed broiler breeder hens
Using animal-mounted sensor technology and machine learning to predict time-to-calving in beef and dairy cows
Using automated in-paddock weighing to evaluate the impact of intervals between liveweight measures on growth rate calculations in grazing beef cattle
Using behavior as an early predictor of sickness in veal calves
Using dorsal surface for individual identification of dairy calves through 3D deep learning algorithms
Using farm management practices to predict Campylobacter prevalence in pastured poultry farms
Using Farm Practice Variables as Predictors of Listeria spp. Prevalence in Pastured Poultry Farms
USING GAUSSIAN PROCESSES TO STUDY TRAUMATIC BRAIN INJURY IN SHEEP
Using GPS Collars and Sensors to Investigate the Grazing Behavior and Energy Balance of Goats Browsing in a Mediterranean Forest Rangeland
Using imagery and computer vision as remote monitoring methods for early detection of respiratory disease in pigs
Using implantable biosensors and wearable scanners to monitor dairy cattle's core body temperature in real-time
Using infrared thermography to detect subclinical mastitis in dairy cows in compost barn systems
Using low-fix rate GPS telemetry to expand estimates of ungulate reproductive success
Using machine learning improves predictions of herd-level bovine tuberculosis breakdowns in Great Britain
Using Machine Learning to Estimate the Heterogeneous Effects of Livestock TransfersJEL codes
Using machine learning to improve the accuracy of genomic prediction of reproduction traits in pigs
Using machine-learning technique for estrus onset detection in dairy cows from acceleration and location data acquired by a neck-tag
Using object-based image analysis to map commercial poultry operations from high resolution imagery to support animal health outbreaks and events
Using Passive Infrared Detectors to Record Group Activity and Activity in Certain Focus Areas in Fattening Pigs
Using phenotypic distribution models to predict livestock performance
Using Pruning-Based YOLOv3 Deep Learning Algorithm for Accurate Detection of Sheep Face
Using real-time ultrasound for in vivo assessment of carcass and internal adipose depots of dairy sheep
Using rumination and activity data for early detection of anaplasmosis disease in dairy heifer calves

Using Sensor Data to Detect Lameness and Mastitis Treatment Events in Dairy Cows: A Comparison of Classification Models
Using state space models to monitor and estimate the effects of interventions on treatment risk and milk yield in dairy farms
Using the Footfall Sound of Dairy Cows for Detecting Claw Lesions
Using the XGBoost algorithm to classify neck and leg activity sensor data using on-farm health recordings for locomotor-associated diseases
Using ultrasound to predict fat deposition in growing lambs of different South African sheep breed types
Using virtual fences to manage grazing: example for heifers and ewes
Utilization of Optical Flow Algorithms to Monitor Development of Tail Biting Outbreaks in Pigs
Utilizing the Microbiota and Machine Learning Algorithms To Assess Risk of Salmonella Contamination in Poultry Rinsate
Vaginal birthing sensors as a tool to monitor calving on large scale applications
Vaginal temperature before calving assessed with wireless vaginal temperature sensor in dairy and beef cattle
Validating the Use of Bovine Buccal Sampling as a Proxy for the Rumen Microbiota by Using a Time Course and Random Forest Classification Approach
Validation and optimisation of an automatic blood sampler for preclinical positron emission tomography research in domestic pigs
Validation of a Commercial Automated Body Condition Scoring System on a Commercial Dairy Farm
Validation of a Commercial Collar-Based Sensor for Monitoring Eating and Ruminating Behaviour of Dairy Cows
Validation of a deep learning-based image analysis system to diagnose subclinical endometritis in dairy cows
Validation of a noseband pressure sensor algorithm as a tool for evaluation of feeding behaviour in dairy Mediterranean buffalo (Bubalus Bubalis)
Validation of a novel milk progesterone-based tool to monitor luteolysis in dairy cows: Timing of the alerts and robustness against missing values
Validation of a real-time location system for zone assignment and neighbor detection in dairy cow groups
Validation of accelerometers to automatically record postures and number of steps in growing lambs
Validation of AfiTagII, a device for automatic measuring of lying behaviour in Holstein and Jersey cows on two different bedding materials
Validation of alternative behavioral observation methods in young broiler chickens
Validation of an Accelerometer Sensor-Based Collar for Monitoring Grazing and Rumination Behaviours in Grazing Dairy Cows
Validation of an automated assay for measurement of bovine plasma ceruloplasmin
Validation of an Automated Body Condition Scoring System Using 3D Imaging
Validation of an automatic scoring system for the assessment of hock burn in broiler

Validation of an ear tag-based accelerometer system for detecting grazing behavior of dairy cows
Validation of an Ultra-Wideband Tracking System for Recording Individual Levels of Activity in Broilers
Validation of automatic systems for monitoring the licking behaviour in Angus and Brahman cattle
Validation of Dairy Cow Bodyweight Prediction Using Traits Easily Recorded by Dairy Herd Improvement Organizations and Its Potential Improvement Using Feature Selection Algorithms
Validation of indwelling vaginal sensor to monitor body temperature in ewes
Validation of NEDAP Monitoring Technology for Measurements of Feeding, Rumination, Lying, and Standing Behaviors, and Comparison with Visual Observation and Video Recording in Buffaloes
Validation of non-invasive brain temperature estimation models during swine therapeutic hypothermia
Validation of Real-Time Kinematic (RTK) Devices on Sheep to Detect Grazing Movement Leaders and Social Networks in Merino Ewes
Validation of the Lifecorder Plus device for accurate recording of the grazing time of dairy goats
Validation of the RumiWatch Converter V0.7.4.5 classification accuracy for the automatic monitoring of behavioural characteristics in dairy cows
Valve-Actuator-Integrated Reference Electrode for an Ultra-Long-Life Rumen pH Sensor
Variable segmentation and ensemble classifiers for predicting dairy cow behaviour
Variable selection for monitoring sickness behavior in lactating dairy cattle with the application of control charts
Variations in the body surface temperature of sows during the post weaning period and its relation to subsequent reproductive performance
Veterinary Care, Milking Routine and Animal Welfare - Present and Future in the Israeli Dairy Sector
Viable smart sensors and their application in data driven agriculture
Video analytic system for detecting cow structure
Virtual Fence Responses Are Socially Facilitated in Beef Cattle
Virtual Fencing Effectively Excludes Cattle from Burned Sagebrush Steppe
Virtual Fencing Is Comparable to Electric Tape Fencing for Cattle Behavior and Welfare
Virtual Fencing Technology Excludes Beef Cattle from an Environmentally Sensitive Area
Virtual Fencing Technology for Cattle Management in the Pasture Feeding System-A Review
Virtual fencing technology to intensively graze lactating dairy cattle. I: Technology efficacy and pasture utilization
Virtual fencing technology to intensively graze lactating dairy cattle. II: Effects on cow welfare and behavior

Virtual fencing without visual cues: Design, difficulties of implementation, and associated dairy cow behaviour
Vision-Based Module for Herding with a Sheepdog Robot
Visual detection on posture transformation characteristics of sows in late gestation based on Libra R-CNN
Visual Guidance and Egg Collection Scheme for a Smart Poultry Robot for Free-Range Farms
Visual identification of individual Holstein-Friesian cattle via deep metric learning
Visual Sensor Placement Optimization with 3D Animation for Cattle Health Monitoring in a Confined Operation
Visual SLAM for Indoor Livestock and Farming Using a Small Drone with a Monocular Camera: A Feasibility Study
Visualization and application of disease diagnosis codes for population health management using porcine diseases as a model
Vocal cues based Decision Support System for estrus detection in water buffaloes (Bubalus bubalis)
Volatile basic nitrogen measurement in digesta using a Berthelot reaction in automated Skalar instrumentation
Volatile compounds monitoring as indicative of female cattle fertile period using electronic nose
Volume and surface area of Holstein dairy cows calculated from complete 3D shapes acquired using a high-precision scanning system: Interest for body weight estimation
VTag: a semi-supervised pipeline for tracking pig activity with a single top-view camera
Waterfowl breeding environment humidity prediction based on the SRU-based sequence to sequence model
Wearable Inertial Sensor-Based Limb Lameness Detection and Pose Estimation for Horses
Wearable Internet of Things enabled precision livestock farming in smart farms: A review of technical solutions for precise perception, biocompatibility, and sustainability monitoring
Wearable multi-sensor enabled decision support system for environmental comfort evaluation of mutton sheep farming
Wearable Wireless Biosensor Technology for Monitoring Cattle: A Review
Weight and volume estimation of poultry and products based on computer vision systems: a review
Weighted Summation: Feature Extraction of Farm Pigsty Data for Electronic Nose
Welfare Assessment on Pasture: A Review on Animal-Based Measures for Ruminants
Welfare Health and Productivity in Commercial Pig Herds
Welfare Quality(R)for dairy cows: towards a sensor-based assessment
What Are Sheep Doing? Tri-Axial Accelerometer Sensor Data Identify the Diel Activity Pattern of Ewe Lambs on Pasture
What type of loafing areas do housed dairy cattle prefer?
When Mobilenetv2 Meets Transformer: A Balanced Sheep Face Recognition Model

Whole genome sequencing and gene sharing network analysis powered by machine learning identifies antibiotic resistance sharing between animals, humans and environment in livestock farming
WSMS: Wearable Stress Monitoring System Based on IoT Multi-Sensor Platform for Living Sheep Transportation
Y-BGD: Broiler counting based on multi-object tracking
"HerdGPS-Preprocessor"-A Tool to Preprocess Herd Animal GPS Data; Applied to Evaluate Contact Structures in Loose-Housing Horses
"Tailception': using neural networks for assessing tail lesions on pictures of pig carcasses
2-D/3-D fusion-based robust pose normalisation of 3-D livestock from multiple RGB-D cameras
